# Impact of frailty and older age on weaning from invasive ventilation: a secondary analysis of the WEAN SAFE study

**DOI:** 10.1186/s13613-025-01435-1

**Published:** 2025-01-20

**Authors:** Caoimhe M. Laffey, Rionach Sheerin, Omid Khazaei, Bairbre A. McNicholas, Tài Pham, Leo Heunks, Giacomo Bellani, Laurent Brochard, Dana Tomescu, Andrew J. Simpkin, John G. Laffey, on behalf of the WEAN SAFE Investigators

**Affiliations:** 1 Anaesthesia and Intensive Care Medicine, School of Medicine, University of Galway, Galway, Ireland; 2 School of Mathematical and Statistical Sciences, University of Galway, Galway, Ireland; 3 Department of Anaesthesia and Intensive Care Medicine, Galway University Hospital, Saolta University Healthcare Group, Galway, Ireland; 4 Department of Anaesthesia and Intensive Care Medicine, Galway University Hospital, Saolta Hospital Group, Galway, Ireland; 5 Service de médecine intensive-réanimation, AP-HP, Hôpital de Bicêtre, DMU CORREVE, FHU SEPSIS, Groupe de recherche CARMAS, Hôpitaux Universitaires Paris-Saclay, Le Kremlin-Bicêtre, France; 6 Université Paris-Saclay, UVSQ, Univ. Paris-Sud, Inserm U1018, Equipe d’Epidémiologie respiratoire intégrative, CESP, Villejuif, 94807 France; 7 Department of Intensive Care, Radboud University Medical Center, Nijmegen, The Netherlands; 8 School of Medicine and Surgery, University of Trento, Trento, Italy; 9 Interdepartmental Division of Critical Care Medicine, University of Toronto, Toronto, Canada; 10 Keenan Research Centre for Biomedical Science, Li Ka Shing Knowledge Institute, St Michael’s Hospital, Unity Health Toronto, Toronto, Canada; 11 Department of Anesthesia and Intensive Care, “Carol Davila” University of Medicine and Pharmacy, Bucharest, Romania; 12 Department of Anesthesiology and Intensive Care, Fundeni Clinical Institute, Sos Fundeni 258 sect 2 zip, Bucharest, 22328 Romania; 13 Department of Anaesthesia and Intensive Care Medicine, School of medicine, Clinical Sciences Institute, University of Galway, Galway, H91 YR71 Ireland

**Keywords:** Clinical frailty scale, Older age, Elderly, Frailty, Ventilator weaning, Ventilator liberation, Invasive mechanical ventilation

## Abstract

**Objective:**

To understand the impact of both frailty and chronologic age on outcomes of weaning from invasive mechanical ventilation (MV).

**Methods:**

The study population consisted of patients enrolled in the ‘WorldwidE. AssessmeNt of Separation of pAtients From ventilatory assistancE (WEAN SAFE) study. We defined 4 non-overlapping groups, namely: ‘frail’ (clinical frailty scale [CFS] score > 4; age < 80 years); ‘elderly’ (CFS ≤ 4; age ≥ 80y), ‘frail \elderly’ (CFS > 4; age ≥ 80 years), and a ‘not frail or elderly’ population. The primary outcome was the impact of frailty and older age on delayed weaning and failed weaning from invasive MV. Secondary outcomes included the impact of frailty and age on ICU and hospital survival.

**Results:**

In the study population, 760 (17%) were frail, while 360 (8%) were elderly, 197 (4%) were frail and elderly, while 3,176 (70%) were not frail or elderly. The frail and elderly cohorts were more likely to be female, had hypoxemic/hypercapnic respiratory failure or sepsis, and had more comorbidities. The proportion of delayed weaning and of failed weaning from invasive MV was significantly higher in the frail (28 and 23%), the elderly (25 and 19%), and the frail and elderly groups (22% and 25%), compared to the not frail or elderly population (12% and 13%, *P* < 0.01). ICU and hospital mortality was higher in the frail (21 and 33%), the elderly (19 and 31%), and the frail and elderly groups (26 and 46%), compared to the not frail or elderly population (12% and 18%, *P* < 0.001). In multivariate analyses, there was an independent association between frailty and delayed weaning initiation and weaning failure. Old age was independently associated with risk of weaning failure.

**Conclusions:**

Frailty status had a more consistent impact than older age on weaning outcomes. However, overall outcomes in these cohorts are encouraging once separation attempts have been initiated.

**Supplementary Information:**

The online version contains supplementary material available at 10.1186/s13613-025-01435-1.

## Introduction

Delayed and failed weaning of patients from invasive mechanical ventilation (MV) worsens patient outcomes, increases the risk of dying and increases length of intensive care unit (ICU) and hospital stay [1–3]. The impact of frailty and older age (i.e. age ≥ 80 years) on the duration and outcomes of weaning from invasive MV is poorly understood and deserves further investigation, especially given that critical care is increasingly required for this subpopulation of patients [4–6].

Older age has been demonstrated in multiple studies to be associated with poorer outcomes in the critically ill [7], including those admitted for acute respiratory distress syndrome (ARDS) [8, 9], acute hypoxaemic respiratory failure [10], sepsis [11] and all-cause critical Illness [12–14]. Frailty is a multidimensional syndrome characterized by diminished physiologic reserve and an increased risk of adverse outcomes after a homeostatic challenge [15]. Frailty is increasingly being studied as a useful construct for identifying adults in various clinical settings who are at high risk of poor outcomes [15]. Frail survivors of critical illness experienced greater impairment in health-related quality of life, functional dependence, and disability compared with those not frail [15–19].

Although frailty and older age are inter-related and frequently co-exist in the critically ill [4, 20], the relative contribution of each to the duration of the weaning process from invasive MV and outcomes of the weaning process are potentially distinct, and remain to be fully understood.

Our primary aim was to determine the impact of frailty and of older age on delayed and failed weaning following invasive MV. Our secondary aims included determination of the impact of frailty and older age on survival, and on end-of-life care, and the risk factors for delayed and failed weaning these patients.

## Materials and methods

This is a pre-defined sub-study of the WEAN-SAFE study, an international, multicentre, prospective cohort study of patients undergoing invasive or non-invasive ventilation, conducted during 4 consecutive weeks in the between October 2017 and June 2018 in a convenience sample of 481 Intensive Care Units (ICUs) from 50 countries, across 5 continents, that recruited 5,859 patients that required at least 2 days of invasive MV [2]. The study, jointly supported by the European Society of Intensive Care Medicine (ESICM) and the European Respiratory Society (ERS), was endorsed by multiple national societies/networks (Appendix 1). National coordinators and site investigators (Appendix 1) were responsible for obtaining ethics committee approval and for ensuring data integrity and validity.

### Patients, study design and data collection

All patients admitted to a participating ICU aged > 16 years and receiving invasive MV two calendar days after intubation were included in the study. Exclusion criteria were age < 16 years or inability to obtain informed consent (where required), and requirement for invasive MV of less than 2 calendar days. Patients transferred to other facilities before successful weaning were deemed lost to follow-up and their ICU and hospital outcomes were not collected. All data were recorded for each patient at the same time each day within participating ICUs, normally as close as possible to 10am each day.

### Data definitions

Our data definitions have been previously reported [2]. Briefly, initiation of weaning from invasive MV is defined as the time the first attempt to separate a patient from the ventilator was performed [21]. This ‘separation attempt’ (SA) included spontaneous breathing trials (SBT), i.e. a short period of decreased or absent ventilator support to predict extubation success, or a direct extubation without SBT. For tracheostomized patients, a SA was defined as a short period of either T-tube trial, low respiratory support, a short period of trach mask oxygenation, or a SBT as declared by the investigator.

As previously reported [2], we used a modified version of the WIND classification [21] to define 5 weaning outcomes:

- “no SA” group: patients who never had a separation attempt in the participating ICU (died or were transferred to another ICU before first SA).
- “short wean” group: patients were successfully weaned < 1 day after the first SA.
- “intermediate wean” group: patients successfully weaned > 1 day but < 7 days following first SA.
- “prolonged wean” group: patients successfully weaned > 7 days after the first SA.
- “failed wean” group: ongoing requirement for invasive ventilatory support at day 90 or at transfer out of the ICU (if sooner), or death (without successful weaning) in patients who underwent a SA.

Delayed weaning initiation is defined as a delay of > 1 day from meeting weaning eligibility criteria [2]. Delayed weaning is defined as requiring > 1 day to wean from invasive MV following the first SA. Successful weaning is defined as no reintubation within 7 days of extubation. Duration of invasive MV was calculated as the number of days between the date of intubation and the date of extubation in ICU (or death, if the patient died while receiving invasive MV). Survival was evaluated at ICU discharge or at hospital discharge up to a 90-day follow-up. Data about limitation of life sustaining measures was reported.

The degree of frailty prior to ICU admission was assessed at the time of study enrollment using the clinical frailty scale (CFS) [16]. This is a global judgment-based approach to frailty identification, which quantifies frailty on a numeric scale matched to descriptors of fitness, comorbidities, vulnerabilities, disability, and life expectancy. The CFS has been previously validated as a useful frailty assessment tool in the critically ill [15–19]. The CFS score was determined by means of patient chart review and discussion with patient surrogates where available.

In the analysis of the weaning process and of ICU and hospital outcomes, 4 non-overlapping patient groups were defined, namely: ‘frail’ (CFS > 4; age < 80 years); ‘elderly’ (CFS ≤ 4; age ≥ 80 years), ‘frail and elderly’ (CFS > 4; age ≥ 80 years), and a comparator ‘not frail or elderly’ group that was neither frail nor elderly (i.e. CFS ≤ 4; age < 80 years).

### Outcomes

The primary outcome of the study was the respective impact of frailty (CFS score > 4), and older age (≥ 80 years), and the combination of frailty and older age, on delayed and failed weaning from invasive MV. Secondary outcomes included determination of the relationship between frailty and older age on ICU and hospital survival, and end-of-life care, and the risk factors for delayed and failed weaning in these subgroups.

### Data management and statistical analyses

Descriptive statistics included proportions for categorical and mean (standard deviation) or median (interquartile range) for continuous variables. The key exposures in our analysis were age (age 80 + and age < 80) and frailty. To investigate the association of age and frailty with weaning duration we used an ordinal logistic regression model, while weaning success was modelled using a logistic regression model. In each model, we adjusted for admission due to cardiac arrest, trauma or non-traumatic neurological event. We further adjusted for weaning factors, such as whether a decision was made to limit life sustaining interventions, P/F ratio, dynamic driving pressure, respiratory rate, PEEP, sedation levels, and SOFA score on the day of first SA. We adjusted for comorbidities of Respiratory, Cardiovascular, Liver, Kidney, Neuromuscular, Immune Compromised, Diabetes and whether the patient received paralyzing medication before the first weaning attempt. These variables were chosen based on previous research on weaning duration in ICU.

We report the Odds Ratios (OR) for the ordinal models along with 95% confidence intervals and p-values. All analyses were carried out in R software, version 4.4. (R Project for Statistical Computing, http://www.R-project.org).

**Fig. 1:**
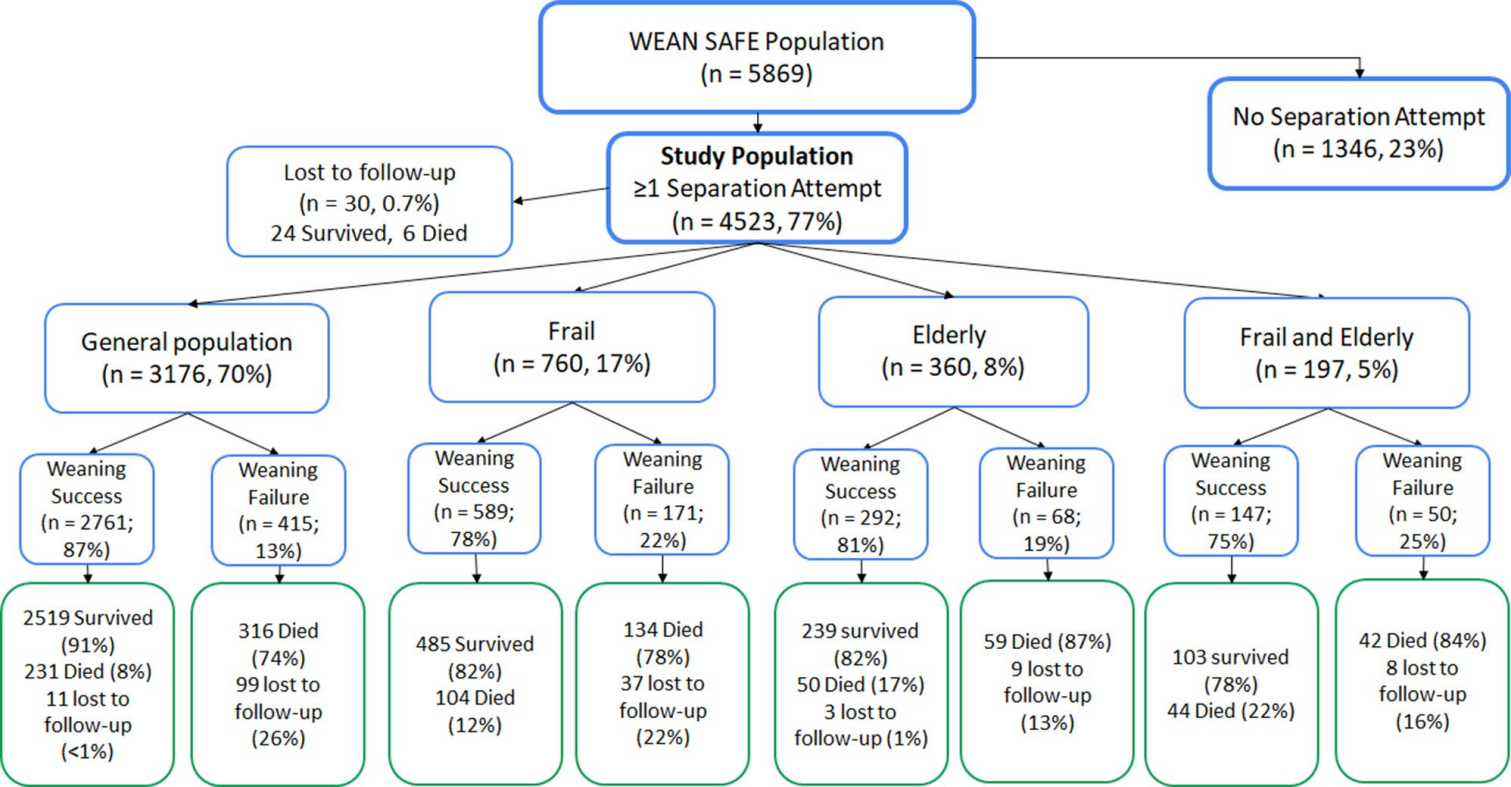
Flow chart for effect of old age and of frailty on the weaning process and outcomes. Of note, patients ‘transferred’ were transferred from the study ICU to another facility while receiving invasive MV, and hence were lost to subsequent followup

**Fig. 2:**
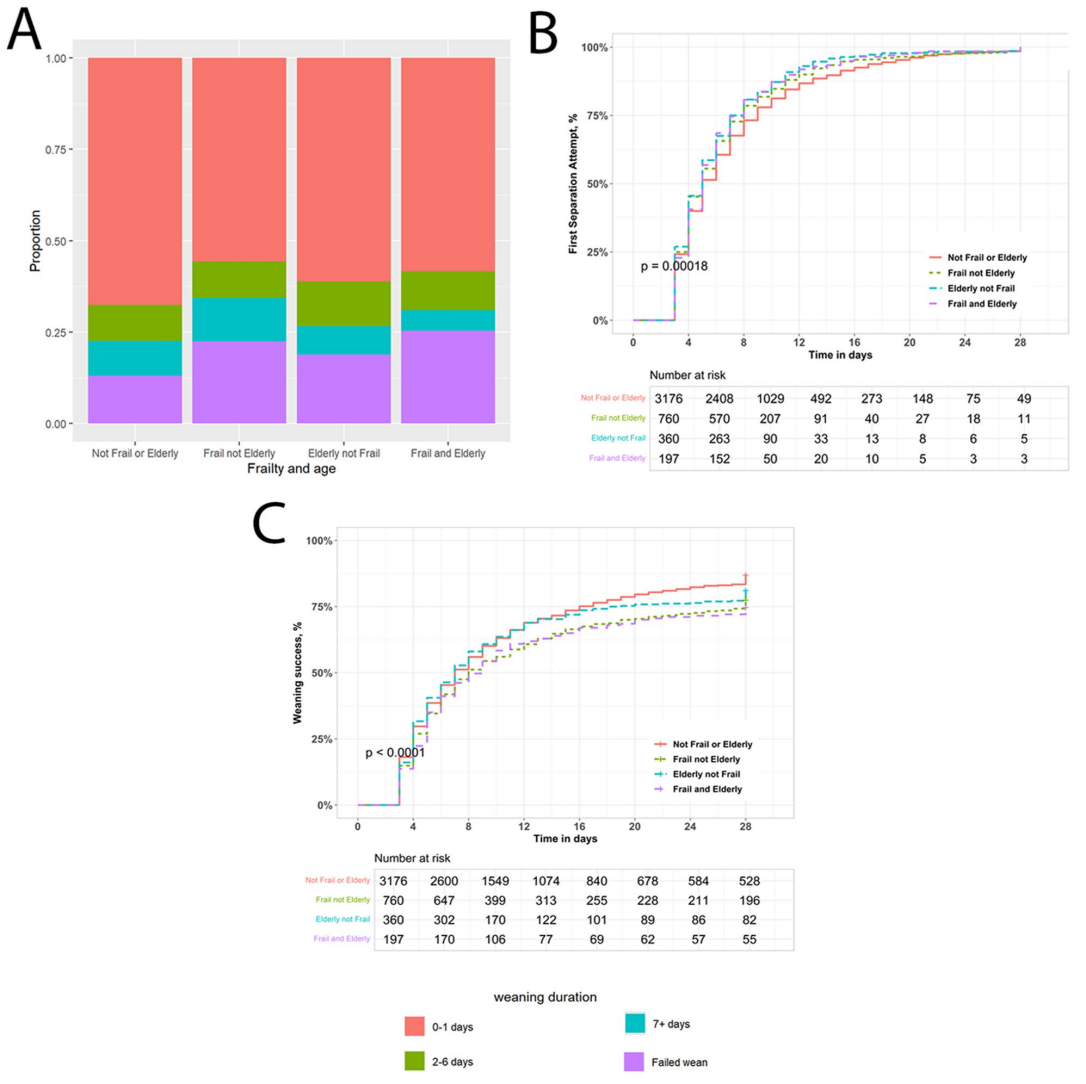
Weaning outcomes in elderly/frail patients weaning from invasive ventilation. Stacked bar chart of impact of older age and of frailty status on weaning outcomes in the study population (**Panel A**). Kaplan-Meier analysis of impact of age/frailty group on likelihood of entering the weaning process to Day 28 (**Panel B**). Kaplan-Meier analysis of weaning success probability over time to Day 28 (**Panel C**)

## Results

Of the 5,869 patients admitted to the participating ICUs from 481 centres across 50 countries worldwide that were still receiving invasive ventilation two calendar days after intubation, 4523 (80%) patients entered the weaning process (i.e. underwent a SA), and constitute the study population (Fig. 1). Of these, 760 (17%) were frail, while 360 (8%) were elderly, 197 (4%) were elderly and frail, while 3,176 (70%) were neither elderly nor frail. The elderly and frail cohorts were more likely to be female, and to be admitted for hypoxemic or hypercapnic respiratory failure or sepsis, and had more comorbidities compared to the not frail or elderly population (Table 1).

**Table 1:** Demographics and outcome data in the elderly and frail subgroups

	General Population (*n* = 3176; 70%)	Frail (*n* = 760; 17%)	Elderly (*n* = 360; 8%)	Frail and Elderly (*n* = 197; 5%)	Missing data *N* (%)	*p*-value*
Sex: Female	1,184 (37%)	295 (39%)	165 (46%)	102 (52%)	30 (0.7)	< 0.001
Age	56 ± 16	63 ± 13	84 ± 3	85 ± 5	30 (0.7)	< 0.001
Body Mass Index	27 ± 7	27 ± 9	26 ± 6	26 ± 6	164 (3.6)	0.009
ICU admission category						
Medical	2023 (63)	600 (79%)	226 (63%)	158 (80%)	30 (0.7)	< 0.001
Planned Surgery	300 (9%)	49 (6%)	23 (6%)	5 (3%)	30 (0.7)	
Trauma	351 (11%)	15 (2.0%)	28 (7.8%)	6 (3%)	30 (0.7)	
Urgent Surgery	502 (16%)	96 (13%)	83 (23%)	28 (14%)	30 (0.7)	
**Cause(s) for ICU admission**						
Hypoxemic respiratory failure	980 (31%)	312 (41%)	124 (34%)	81 (41%)	30 (0.7)	< 0.001
Sepsis	654 (21%)	215 (28%)	77 (21%)	55 (28%)	30 (0.7)	< 0.001
Hypercapnic respiratory failure	379 (12%)	175 (23%)	58 (16%)	48 (24%)	30 (0.7)	< 0.001
Non-traumatic neurologic event	470 (15%)	97 (13%)	56 (16%)	32 (16%)	30 (0.7)	0.4
Emergency surgery	468 (15%)	87 (11%)	64 (18%)	22 (11%)	30 (0.7)	0.014
Airway protection	406 (13%)	88 (12%)	36 (10%)	32 (16%)	30 (0.7)	0.14
Cardiac arrest	253 (8.0%)	65 (8.6%)	26 (7.2%)	19 (9.6%)	30 (0.7)	0.7
**Comorbidities and risk factors**						
Respiratory	576 (18%)	262 (34%)	71 (20%)	65 (33%)	30 (0.7)	< 0.001
Cardiovascular	242 (7.6%)	152 (20%)	49 (14%)	57 (29%)	30 (0.7)	< 0.001
Liver	135 (4.3%)	60 (7.9%)	3 (0.8%)	1 (0.5%)	30 (0.7)	< 0.001
Kidney	241 (7.6%)	131 (17%)	49 (14%)	47 (24%)	30 (0.7)	< 0.001
Neuromuscular	528 (17%)	345 (45%)	65 (18%)	106 (54%)	31 (0.7)	< 0.001
Immune Dysfunction	411 (13%)	167 (22%)	26 (7.2%)	33 (17%)	30 (0.7)	< 0.001
Diabetes	609 (19%)	213 (28%)	94 (26%)	52 (26%)	30 (0.7)	< 0.001
**Weaning Milestones**						
**Weaning Outcomes**						
Failed Weaned	415 (13%)	171 (23%)	68 (19%)	50 (25%)	2 (0.04)	< 0.001
Successfully Weaned	2,761 (87%)	589 (78%)	292 (81%)	147 (75%)	28 (0.62)	
**Weaning Duration (successfully weaned)**						
Short Wean (≤ 1 day)	2,144 (78%)	423 (72%)	220 (75%)	115 (78%)	25 (0.5)	0.007
Intermediate Wean (2-7d)	314 (11%)	76 (13%)	44 (15%)	21 (14%)	2 (0.04)	
Prolonged Wean (> 7 days)	303 (11%)	90 (15%)	28 (10%)	11 (8%)	1 (0.02)	
**Delayed initiation of weaning**	1,506 (47%)	351 (46%)	162 (45%)	106 (54%)	30 (0.7)	0.2
**Outcomes**						
Total duration of invasive mechanical ventilation, days	7 (4, 12)	7 (4, 13)	6 (4, 11)	7 (4, 11)	183 (4.0)	0.3
Length of ICU stay, days	11 (7, 18)	11 (7, 18)	10 (6, 16)	10 (7, 17)	184 (4.1)^*$*§^	0.2
Length of hospital stay, days	23 (14, 40)	24 (14, 40)	21 (12, 36)	20 (12, 32)	243 (5.4)^*$*§^	0.027
Limitation of life sustaining interventions	386 (12%)	203 (27%)	90 (25%)	68 (35%)	30 (0.7)	< 0.001
ICU mortality, n (%)	366 (12%)	149 (21%)	66 (19%)	50 (26%)	183 (4.0)^$^	< 0.001
Hospital mortality, n (%)	547 (18%)	238 (33%)	109 (31%)	86 (46%)	197(4.4)^$§^	< 0.001

* p value comparison between the four groups. Kruskal-Wallis for continuous and F-test for categorical

$: For patients transferred to other institutions still receiving invasive mechanical ventilation (*n* = 390), follow-up stopped at transfer from participating ICU and mortality beyond this point was not collected

§: Among patients discharged alive from the participating ICU, 1 has missing data for ICU length of stay, 62 for hospital length of stay and 14 for hospital mortality

**Table 2:** Univariate (left, *n* = 4523) and multivariable (right, *n* = 3594) logistic regression models of delayed initiation of weaning

	Unadjusted Odds Ratio	95% CI	*p*	Adjusted Odds Ratio	95% CI	*p*	
Age 80+	1.03	0.86, 1.22	0.78	1.08	0.87, 1.35	0.46	
Frail	1.02	0.89, 1.18	0.75	1.29	1.07, 1.56	**0.01**	
**Comorbidities**							
Respiratory	0.87	0.76, 1.01	0.07	1.03	0.86, 1.23	0.73	
Cardiovascular	0.60	0.5, 0.73	**< 0.01**	0.62	0.48, 0.78	**< 0.01**	
Liver	0.98	0.74, 1.3	0.89	1.02	0.71, 1.46	0.91	
Kidney	0.94	0.78, 1.14	0.53	0.95	0.75, 1.22	0.70	
Neuromuscular	1.15	1, 1.32	**0.05**	1.10	0.92, 1.31	0.31	
Immune Compromised	0.92	0.78, 1.09	0.35	1.11	0.91, 1.37	0.30	
Diabetes	1.04	0.9, 1.2	0.59	1.13	0.95, 1.35	0.16	
**Reason for ICU Admission**							
Cardiac Arrest	0.86	0.69, 1.06	0.16	0.74	0.57, 0.96	**0.02**	
Trauma	1.81	1.44, 2.27	**< 0.01**	1.81	1.37, 2.4	**< 0.01**	
Neurologic (non-trauma)	1.39	1.18, 1.64	**< 0.01**	1.27	1.03, 1.57	**0.03**	
**Lung Injury Indices**							
P/F ratio	1.00	1, 1	**< 0.01**	1.00	1, 1	**< 0.01**	
Respiratory Rate	1.03	1.02, 1.04	**< 0.01**	1.05	1.04, 1.06	**< 0.01**	
PEEP	0.88	0.85, 0.91	**< 0.01**	0.86	0.83, 0.9	**< 0.01**	
Driving Pressure	0.97	0.96, 0.98	**< 0.01**	0.96	0.95, 0.97	**< 0.01**	
SOFA score (non-neuro)	0.98	0.96, 0.99	**< 0.01**	1.00	0.98, 1.02	0.97	
Use of paralyzing medications	1.51	1.21, 1.89	**< 0.01**	1.80	1.37, 2.36	**< 0.01**	
**Sedation on the first day fulfilling WEC (Reference: awake)**							
Moderately sedated	1.87	1.6, 2.19	**< 0.01**	1.95	1.62, 2.34	**< 0.01**	
Deeply sedated	4.15	3.51, 4.92	**< 0.01**	4.16	3.41, 5.08	**< 0.01**	

**Table 3:** Univariate (left, *n* = 3817) and multivariable (right, *n* = 3022) ordinal logistic regression models of likelihood of intermediate or prolonged weaning duration

	Unadjusted Odds Ratio	95% CI	*p*	Adjusted Odds Ratio	95% CI	*p*
Age 80+	0.97	0.77, 1.22	0.79	0.97	0.74, 1.26	0.81
Frail	1.27	1.06, 1.52	**0.01**	1.25	0.99, 1.57	0.06
**Comorbidities**						
Respiratory	1.13	0.95, 1.35	0.17	1.10	0.89, 1.36	0.38
Cardiovascular	0.81	0.63, 1.03	0.09	0.80	0.59, 1.06	0.13
Liver	1.04	0.71, 1.48	0.85	0.97	0.61, 1.49	0.88
Kidney	1.07	0.83, 1.36	0.61	1.15	0.85, 1.55	0.36
Neuromuscular	1.23	1.04, 1.46	**0.02**	1.19	0.96, 1.47	0.10
Immune Compromised	0.93	0.74, 1.16	0.53	0.98	0.75, 1.27	0.87
Diabetes	0.83	0.69, 1.00	**0.05**	0.83	0.66, 1.03	0.09
**Reason for ICU Admission**						
Cardiac Arrest	1.06	0.78, 1.42	0.69	1.17	0.83, 1.62	0.37
Trauma	1.31	1, 1.69	0.04	1.47	1.08, 1.99	**0.01**
Neurologic (non-trauma)	1.38	1.13, 1.69	**< 0.01**	1.61	1.26, 2.05	**< 0.01**
**Lung Injury Indices on the day of first SA**						
P/F ratio	1.00	1, 1	**0.01**	1.00	1, 1	0.36
Respiratory Rate	1.06	1.05, 1.07	**< 0.01**	1.06	1.05, 1.08	**< 0.01**
PEEP	1.09	1.05, 1.13	**< 0.01**	1.10	1.05, 1.15	**< 0.01**
Driving Pressure	0.99	0.98, 1.01	0.36	0.99	0.98, 1.01	0.26
SOFA score (non-neuro)	1.02	1, 1.04	**0.02**	1.00	0.98, 1.03	0.74
Use of paralyzing medications	1.25	0.95, 1.64	0.10	1.12	0.82, 1.53	0.46
**Sedation on the first day fulfilling WEC (Reference: awake)**						
Moderately sedated	1.25	1.03, 1.51	**0.02**	1.39	1.11, 1.74	**< 0.01**
Deeply sedated	1.43	1.16, 1.76	**< 0.01**	1.64	1.29, 2.09	**< 0.01**

**Table 4:** Univariable (left, *n* = 4523) and multivariable (right, *n* = 3594) and logistic regression models of wean failure

	Unadjusted Odds Ratio	95% CI	*p*	Adjusted Odds Ratio	95% CI	*p*
Age 80+	1.54	1.23, 1.91	**< 0.01**	1.60	1.22, 2.09	**< 0.01**
Frail	1.90	1.59, 2.27	**< 0.01**	1.74	1.37, 2.2	**< 0.01**
**Comorbidities**						
Respiratory	1.40	1.16, 1.68	**< 0.01**	1.28	1.02, 1.6	**0.03**
Cardiovascular	0.88	0.67, 1.14	0.35	0.65	0.47, 0.9	**0.01**
Liver	1.38	0.96, 1.95	0.08	1.59	1.01, 2.44	**0.04**
Kidney	1.54	1.21, 1.95	**< 0.01**	1.44	1.06, 1.93	**0.02**
Neuromuscular	1.16	0.96, 1.4	0.11	0.97	0.77, 1.23	0.81
Immune Compromised	1.69	1.37, 2.07	**< 0.01**	1.91	1.48, 2.45	**< 0.01**
Diabetes	1.16	0.96, 1.4	0.12	1.08	0.86, 1.36	0.49
**Reason for ICU Admission**						
Cardiac Arrest	2.81	2.21, 3.55	**< 0.01**	3.14	2.36, 4.17	**< 0.01**
Trauma	0.71	0.5, 0.98	**0.05**	1.26	0.84, 1.85	0.24
Neurologic (non-trauma)	1.22	0.98, 1.52	0.07	1.43	1.07, 1.88	**0.01**
**Lung Injury Indices**						
P/F ratio	1.00	1, 1	**< 0.01**	1.00	1, 1	0.47
Respiratory Rate	1.06	1.04, 1.07	**< 0.01**	1.05	1.04, 1.07	**< 0.01**
PEEP	1.10	1.06, 1.15	**< 0.01**	1.08	1.03, 1.14	**< 0.01**
Driving Pressure	1.04	1.03, 1.05	**< 0.01**	1.03	1.02, 1.05	**< 0.01**
SOFA score (non-neuro)	1.05	1.03, 1.07	**< 0.01**	1.03	1.01, 1.06	**0.01**
Use of paralyzing medications	1.40	1.05, 1.83	**0.02**	1.15	0.82, 1.59	0.42
**Sedation on the first day fulfilling WEC**						
Moderately sedated	1.00	0.81, 1.25	0.97	1.04	0.8, 1.35	0.77
Deeply sedated	1.91	1.54, 2.37	**< 0.01**	1.88	1.45, 2.44	**< 0.01**

### Weaning milestones

The proportion of patients with delayed weaning from invasive MV was significantly higher in the frail (28%), the elderly (25%), and the frail and elderly groups (22%), compared to the not frail or elderly population (12%, *P* < 0.01) (Fig. 2A, Figure e1a). The proportion of patients with failed weaning from invasive MV was significantly higher in the frail (23%), the elderly (19%), and the frail and elderly groups (25%), compared to the not frail or elderly population (13%, *P* < 0.001) (Fig. 2B-C, Figure e1b). There was a progressive increase in the proportion of patients experiencing prolonged weaning and failed weaning with increasing CFS score (Figure e2A), and with each decade of increasing age over 60 years (Figure e2B). There was no effect of age or frailty status on the frequency of delayed initiation of weaning (Table 1) or on the likelihood of entering the weaning process (Fig. 2B).

### Clinical outcomes

**Fig. 3:**
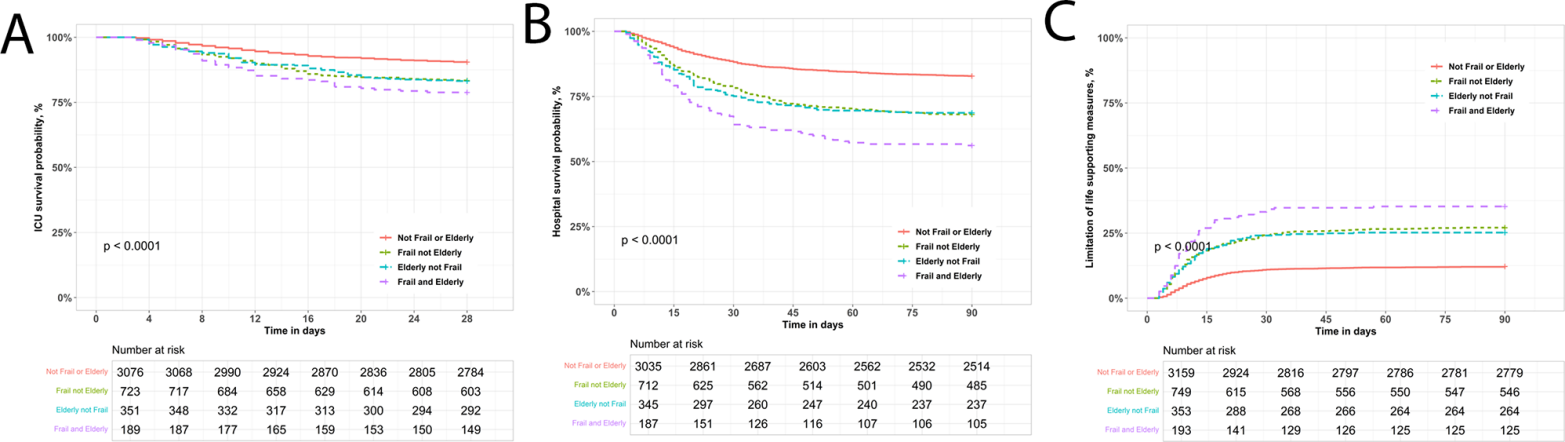
Clincial outcomes in elderly/frail patients weaning from invasive ventilation. Kaplan-Meier analysis of ICU survival probability over time to Day 28 (**Panel A**). Kaplan-Meier analysis of hospital survival probability over time to Day 90 (**Panel B**). Kaplan-Meier plot of impact of age/frailty on probability of limitation of life supporting measures (**Panel C**)

ICU survival rates were significantly lower in the elderly, the frail and the frail and elderly groups, compared to the not frail or elderly population (Table 1; Fig. 3A). The frail and elderly group had the highest ICU mortality, with the frail and the elderly categories intermediate, compared to the not frail or elderly population (Fig. 3A).

Hospital survival rates were also significantly lower in the elderly, the frail and the frail and elderly groups, compared to the not frail or elderly population (Table 1; Fig. 3B). The frail and elderly had the highest hospital mortality at 44%, with the frail and elderly categories intermediate (33% and 31% respectively) compared to the not frail or elderly population (Table 1; Fig. 3B). Limitation of life supporting measures was highest in the frail and elderly group (35%), and intermediate in the frail (21%) and the elderly (19%), compared to the not frail or elderly population (12%) (Table 1; Fig. 3C).

**Fig. 4:**
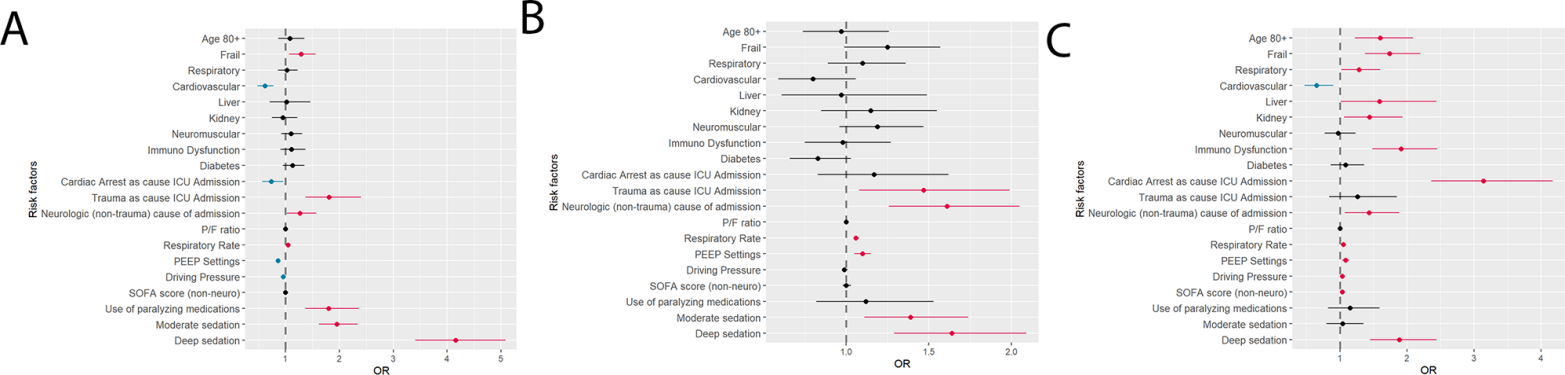
Multivariate analyses of factors associated with weaning process. Multivariate analysis risk factors for delayed initiation of weaning (**Panel A**), longer duration of weaning (**Panel B**), and failed weaning (**Panel C**), in patients that entered the weaning process

### Relationship between Frailty and older age and weaning

In multivariate analyses, there was an independent association between frailty status, but not with older age, and risk of delayed initiation of weaning (Table 2, Fig. 4*A*). Potentially reversible factors associated with delayed weaning initiation included use of paralyzing medications, and the presence of moderate or deep sedation at the time of fulfilling weaning eligibility criteria (Table 2, Fig. 4*A*). Neither frailty status nor older age were associated with risk of prolonged weaning from invasive MV (Table 3, Fig. 4*B*). Both frailty status and older age were associated with risk of failed weaning from invasive MV (Table 4, Fig. 4*C*). Potentially reversible factors associated with failed weaning included higher respiratory rate, higher PEEP, higher driving pressure, and the presence of deep sedation at the time of fulfilling weaning eligibility criteria.

In multivariate analyses of the elderly and frailty cohorts, there was an association between deep sedation at the time when weaning criteria were fulfilled and the risk of weaning failure in the frail cohort (Table e1) but not in the elderly cohort (Table e2).

## Discussion

While older age, frailty are inter-related conditions, the contribution of each to the process and outcomes of the weaning process are potentially distinct [20]. We report that outcomes in patients requiring invasive MV for 2 or more days are poorer in both the elderly and the frail cohorts, and generally substantially worse in the cohort that were both elderly and frail. The elderly and frail cohorts were more likely to be female, which contrasts with the fact that more males are admitted to ICU overall. The elderly and frail cohort was also more likely to be admitted for hypoxemic or hypercapnic respiratory failure or sepsis, and had more comorbidities compared to the not frail or elderly population.

In regard to the relative contributions of older age and frailty, frailty per se appeared to have a more consistent impact on overall outcomes and on weaning duration and success rates, with a progressively worsening outcome with increasing frailty category. However, it should be emphasised that outcomes in these cohorts were encouraging overall, with hospital survival rates over 50% in the elderly and frail patients that commenced the weaning process.

### Impact of older age

The cohort of elderly patients presenting for critical care is increasing, a natural consequence arising from global increases in life expectancy [20]. The WHO predict that there will be a tripling of the number of elderly patients aged 80 years or older between 2019 and 2050 [22]. This will generate an increased demand for critical care in our very elderly populations [7]. Recent studies from Australia [12], Finland [13], Denmark [23], Germany [24], South Korea [25], France [26], the Netherlands [27], Canada [28] and a large scale study across 21 European countries [20], have shown encouraging outcomes even in the very elderly. Previous assumptions regarding poorer outcomes and limited survival time gain for the very elderly admitted to ICU are increasingly questioned [29], and an increasing proportion of very elderly patients receive high intensity critical care, including invasive MV [30]. A quarter of patients aged 80 or older make a full recovery post critical illness, returning to baseline physical function at 1 year [31].

Most of studies of outcomes in the very elderly only include a minority of patients that require invasive MV. Consequently, outcomes in this patient cohort receiving invasive MV, particularly when required for a period of 2 or more days, indicating these patients are at high risk for poorer outcomes, have yet to be clearly defined. Our study demonstrates that the impact of age on outcomes from weaning from invasive MV, while present, was relatively limited. In patients aged over 80 that were not frail, and who entered the weaning process, weaning durations were longer, but 81% successfully weaned, while 67% survived to hospital discharge. In multivariate analyses, old age was independently associated with risk of weaning failure but not with delayed initiation of weaning or with increased weaning duration category. Taken together, these data suggest that older age per se is not a useful criterion for making decisions regarding provision of critical care.

### Impact of Frailty

The adverse impact of frailty, a condition of diminished physiologic reserve, is increasingly appreciated in patients with critical illnesses. Frail survivors of critical illness experienced greater impairment in health-related quality of life, functional dependence, and disability compared with those not frail [15–20]. Longer term outcomes in survivors of critical illness are worse in patients with pre-existing frailty, with higher rates of hospital readmission and death than patients without frailty [32]. The impact of frailty on outcomes in critically ill patients receiving invasive MV, particularly when required for significant time periods has yet to be clearly defined.

The proportion of frail patients in our study population was high, comprising over one fifth of the whole cohort. The impact of frailty on outcomes from weaning from invasive ventilation was substantial and consistent, with a progressive worsening of outcomes with greater degrees of frailty. Outcomes in the most severely frail category were particularly poor, with only one third weaning from ventilation. In multivariate analyses, frailty was independently associated with both delayed weaning initiation and failed weaning. Of interest, there was an independent association between deep sedation at time when weaning criteria are fulfilled and the risk of weaning failure in the frail cohort, a finding not seen in the elderly cohort.

Taken together, these data suggest that it is important to consider the presence and degree of frailty when making decisions in patients weaning from invasive MV. Particular attention to depth of sedation at the timepoint where frail patients are ready to wean may be important [2]. Our study also supports prior studies demonstrating that routine large scale population screening for frailty degree in patients with critical illness is possible and is prognostically useful, facilitating critical care planning for this important and growing patient cohort [33].

## Strengths and limitations

Our study examines the impact of chronological age and frailty status, in patients weaning from invasive MV, performed in a large, and globally diverse, patient cohort. Nevertheless, there are important limitations to consider. While all raw data was entered directly into the electronic case report form, the interpretation of source data was performed by on-site clinicians, which potentially increased variability. To ensure data quality, we instituted a robust data quality control program as previously described [2]. Participating hospitals were representative of different levels of care and geography but despite enrolling a large number of ICUs from around the world, our convenience sample may be prone to selection biases. Our assumption that patients discharged from the hospital before day 90 were alive at that time point is a further limitation. Lastly, a small proportion of patients (4%) were lost to follow-up because they were transferred prior to the first separation attempt.

## Conclusions

Our study examines the impact of chronological age versus physiologic reserve, as assessed by frailty status, in patients weaning from invasive MV. Patient frailty status appeared to have a more consistent impact than older age on overall outcomes and on weaning duration and success rates. However, it should be emphasised that overall outcomes in these cohorts were encouraging, with survival rates over 50% in the elderly and frail patients that commenced the weaning process. Sedation status at time of weaning commencement may be an important, and potentially modifiable factor in frail patients, that if addressed could reduce weaning delays and weaning failure rates.

## Electronic supplementary material

Below is the link to the electronic supplementary material.

Supplementary Material 1

Supplementary Material 2

Supplementary Material 3

Supplementary Material 4

Supplementary Material 5

## Data Availability

The data in this manuscript are owned by the individual contributing institutions of the WEAN SAFE investigators. Requests for data should be made to the WEAN SAFE Executive Committee, by way of email to the corresponding author. Any data provided will consist of de-identified participant with data dictionary, be restricted to the data presented in this paper, and be subject to a data sharing agreement.
