# Correction: Impact of frailty and older age on weaning from invasive ventilation: a secondary analysis of the WEAN SAFE study

**DOI:** 10.1186/s13613-025-01467-7

**Published:** 2025-04-28

**Authors:** Caoimhe M. Laffey, Rionach Sheerin, Omid Khazaei, Bairbre A. McNicholas, Tài Pham, Leo Heunks, Giacomo Bellani, Laurent Brochard, Dana Tomescu, Andrew J. Simpkin, John G. Laffey, on behalf of the WEAN SAFE Investigators

**Affiliations:** 1 Anaesthesia and Intensive Care Medicine, School of Medicine, University of Galway, Galway, Ireland; 2 School of Mathematical and Statistical Sciences, University of Galway, Galway, Ireland; 3 Department of Anaesthesia and Intensive Care Medicine, Galway University Hospital, Saolta University Healthcare Group, Galway, Ireland; 4 Department of Anaesthesia and Intensive Care Medicine, Galway University Hospital, Saolta Hospital Group, Galway, Ireland; 5 Service de médecine intensive-réanimation, AP-HP, Hôpital de Bicêtre, DMU CORREVE, FHU SEPSIS, Groupe de recherche CARMAS, Hôpitaux Universitaires Paris-Saclay, Le Kremlin-Bicêtre, France; 6 Université Paris-Saclay, UVSQ, Univ. Paris-Sud, Inserm U1018, Equipe d’Epidémiologie respiratoire intégrative, CESP, Villejuif, 94807 France; 7 Department of Intensive Care, Radboud University Medical Center, Nijmegen, The Netherlands; 8 School of Medicine and Surgery, University of Trento, Trento, Italy; 9 Interdepartmental Division of Critical Care Medicine, University of Toronto, Toronto, Canada; 10 Keenan Research Centre for Biomedical Science, Li Ka Shing Knowledge Institute, St Michael’s Hospital, Unity Health Toronto, Toronto, Canada; 11 Department of Anesthesia and Intensive Care, “Carol Davila” University of Medicine and Pharmacy, Bucharest, Romania; 12 Department of Anesthesiology and Intensive Care, Fundeni Clinical Institute, Sos Fundeni 258 sect 2 zip, Bucharest, 22328 Romania; 13 Department of Anaesthesia and Intensive Care Medicine, School of medicine, Clinical Sciences Institute, University of Galway, Galway, H91 YR71 Ireland

**Correction: Annals of Intensive Care (2025) 15:13** 10.1186/s13613-025-01435-1

The Supplementary [collaborating authors from WEAN SAFE] was missing from this article and has now been uploaded.

The original article has been corrected.

## Supplementary Information

Supplementary Material 1.

Supplementary Material 2.

