# Supplementary material for: Impact of frailty and older age on weaning from invasive ventilation: a secondary analysis of the WEAN SAFE study
Source: Ann Intensive Care. 2025 Jan 20;15:13. doi: 10.1186/s13613-025-01435-1 (PMC11743409; doi:10.1186/s13613-025-01435-1)
Supplement: Supplementary file 1 — Supplementary Material 1 [file 13613_2025_1435_MOESM1_ESM.docx]

**Electronic Supplemental Material - Appendices**

***Title***: Impact of frailty and older age on weaning from invasive ventilation: A secondary analysis of the WEAN SAFE study.

1. **Appendix 1: List of WEAN SAFE National Collaborators**
2. **Appendix 2: List WEAN SAFE Collaborating Authors**
3. **Appendix 3: List Participating ICUs**
4. **Appendix 4: List endorsing Societies**

**Appendix 1: WEAN SAFE National Coordinators**

| **First name** | **Surname** |
| --- | --- |
| Fekri | Abrough |
| Subhash P | Acharya |
| Pravin | Amin |
| Yaseen | Arabi |
| Irene | Aragao |
| Philippe | Bauer |
| Gaëtan | Beduneau |
| Jeremy | Beitler |
| Johan | Berkius |
| Guillermo | Bugedo |
| Luigi | Camporota |
| Vladimir | Cerny |
| Young-Jae | Cho |
| Kevin | Clarkson |
| Elisa | Estenssoro |
| Ewan | Goligher |
| Giacomo | Grasselli |
| Alexey | Gritsan |
| Seyed Mohammadreza | Hashemian |
| Greet | Hermans |
| Leo M | Heunks |
| Bojan | Jovanovic |
| Kiyoyasu | Kurahashi |
| Jon Henrik | Laake |
| Dimitrios | Matamis |
| Onnen | Moerer |
| Zsolt | Molnar |
| Ezgi | Ozylmaz |
| Bernardo | Panka |
| Alfred | Papali |
| Óscar | Peñuelas |
| Sébastien | Perbet |
| Lise | Piquilloud |
| Haibo | Qiu |
| Assem Abdel | Razek |
| Nuttapol | Rittayamai |
| Rollin | Roldan |
| Ary | Serpa Neto |
| Konstanty | Szuldrzynski |
| Daniel | Talmor |
| Dana | Tomescu |
| Frank | Van Haren |
| Asisclo | Villagomez |
| Amine Ali | Zeggwagh |

**Appendix 2: List WEAN SAFE collaborating Authors**

| **First name** | **Surname** |
| --- | --- |
| Toshikazu | Abe |
| Abdelrhman | Aboshady |
| Melanie | Acampo-de Jong |
| Subhash | Acharya |
| Jane | Adderley |
| Nalan | Adiguzel |
| Vijay Kumar | Agrawal |
| Gerardo | Aguilar |
| Gaston | Aguirre |
| Hernan | Aguirre-Bermeo |
| Björn | Ahlström |
| Türkay | Akbas |
| Mustafa | Akker |
| Ghamdan | Al Sadeh |
| Sultan | Alamri |
| Angela | Algaba |
| Muneeb | Ali |
| Anna | Aliberti |
| Jose Manuel | Allegue |
| Diana | Alvarez |
| Joaquin | Amador |
| Finn H | Andersen |
| Sharique | Ansari |
| Yutthana | Apichatbutr |
| Olympia | Apostolopoulou |
| Yaseen | Arabi |
| Daniel | Arellano |
| Mestanza | Arica |
| Huseyin | Arikan |
| Koichi | Arinaga |
| Jean-Michel | Arnal |
| Kengo | Asano |
| Marta | Asín-Corrochano |
| Jesus Milagrito | Avalos Cabrera |
| Silvia | Avila Fuentes |
| Semih | Aydemir |
| Gulbin | Aygencel |
| Luciano | Azevedo |
| Feza | Bacakoglu |
| Julio | Badie |
| Elias | Baedorf Kassis |
| Gabriela | Bai |
| Govindan | Balaraj |
| Bruno | Ballico |
| Valerie | Banner-Goodspeed |
| Preveen | Banwarie |
| Rosella | Barbieri |
| Arvind | Baronia |
| Jonathan | Barrett |
| Loïc | Barrot |
| Jesus Emilio | Barrueco-Francioni |
| Jeffrey | Barry |
| Philippe | Bauer |
| Harshal | Bawangade |
| Sarah | Beavis |
| Eduardo | Beck |
| Nina | Beehre |
| Alberto | Belenguer Muncharaz |
| Giacomo | Bellani |
| Mirko | Belliato |
| Agrippino | Bellissima |
| Rodrigo | Beltramelli |
| Asma | Ben Souissi |
| Adela | Benitez-Cano |
| Mohamed | Benlamin |
| Abdellatif | Benslama |
| Luis | Bento |
| Daniela | Benvenuti |
| Johan | Berkius |
| Laura | Bernabe |
| Andrew | Bersten |
| Giacomo | Berta |
| Pietro | Bertini |
| Elliot | Bertram-Ralph |
| Mohamed | Besbes |
| Lisandro Roberto | Bettini |
| Pascal | Beuret |
| Jeremy | Bewley |
| Marco | Bezzi |
| Lakshay | Bhakhtiani |
| Rakesh | Bhandary |
| Kaushik | Bhowmick |
| Shailesh | Bihari |
| Bernie | Bissett |
| David | Blythe |
| Simon | Bocher |
| Narain | Boedjawan |
| Christine M | Bojanowski |
| Elisa | Boni |
| Sabrina | Boraso |
| Massimo | Borelli |
| Silvina | Borello |
| Margarita | Borislavova |
| Karen J | Bosma |
| Maurizio | Bottiroli |
| Owen | Boyd |
| Suha | Bozbay |
| Arturo | Briva |
| Laurent | Brochard |
| Cédric | Bruel |
| Andrea | Bruni |
| Ulrike | Buehner |
| Guillermo | Bugedo |
| Pierre | Bulpa |
| Karen | Burt |
| Mathieu | Buscot |
| Stefania | Buttera |
| Jorge | Cabrera |
| Roberta | Caccese |
| Pietro | Caironi |
| Ivan | Canchos Gutierrez |
| Nancy | Canedo |
| Alma | Cani |
| Iacopo | Cappellini |
| Jesus | Carazo |
| Luis Pablo | Cardonnet |
| David | Carpio |
| Demetrio | Carriedo |
| Ramón | Carrillo |
| João | Carvalho |
| Eliana | Caser |
| Antonio | Castelli |
| Manuel | Castillo Quintero |
| Heloisa | Castro |
| Nuno | Catorze |
| Melike | Cengiz |
| Enrique | Cereijo |
| Helga | Ceunen |
| Christos | Chaintoutis |
| Youjin | Chang |
| Gustavogcha | Chaparro |
| Carmel | Chapman |
| Simon | Chau |
| Cecilia Eugenia | Chavez |
| Cosimo | Chelazzi |
| Jonathan | Chelly |
| Frank | Chemouni |
| Kai | Chen |
| Ariel | Chena |
| Paolo | Chiarandini |
| Phil | Chilton |
| Davide | Chiumello |
| Young-Jae | Cho |
| Yvette | Chou-Lie |
| Nicolas | Chudeau |
| Ismail | Cinel |
| Gilda | Cinnella |
| Michele | Clark |
| Thomas | Clark |
| Kevin | Clarkson |
| Stefano | Clementi |
| Luis | Coaguila |
| Alexis Jaspe | Codecido |
| Amy | Collins |
| Riccardo | Colombo |
| Juan | Conde |
| Guglielmo | Consales |
| Tim | Cook |
| Andrea | Coppadoro |
| Rodrigo | Cornejo |
| Andrea | Cortegiani |
| Cristina | Coxo |
| Andrea Neville | Cracchiolo |
| Mónica | Crespo Ramirez |
| Philippe | Crova |
| José | Cruz |
| Lucia | Cubattoli |
| Zafer | Çukurova |
| Francesco | Curto |
| Piotr | Czempik |
| Rocco | D’Andrea |
| Fernando | da Silva Ramos |
| Laurence | Dangers |
| Marc | Danguy des Déserts |
| Pierre-Eric | Danin |
| Fabianne | Dantas |
| Cédric | Daubin |
| Wu | Dawei |
| Candelaria | de Haro |
| Felipe | de Jesus Montelongo |
| Diego | De Mendoza |
| Raúl | de Pablo |
| Gennaro | De Pascale |
| Silvia | De Rosa |
| Maxens | Decavèle |
| Pierre-Louis | Declercq |
| Alberto | Deicas |
| María | del Carmen Campos Moreno |
| Jean | Dellamonica |
| Benjamin | Delmas |
| Oktay | Demirkiran |
| Hilmi | Demirkiran |
| Tarek | Dendane |
| Rossella | di Mussi |
| Chrysi | Diakaki |
| Anatilde | Diaz |
| Willy | Diaz |
| Yalim | Dikmen |
| Aikaterini | Dimoula |
| Patricia | Doble |
| Nagwa | Doha |
| Guilherme | Domingos |
| Martin | Dres |
| David | Dries |
| Abhijit | Duggal |
| Graeme | Duke |
| Pavel | Dunts |
| Knut | Dybwik |
| Maksym | Dykyy |
| Philippe | Eckert |
| Serdar | Efe |
| Souheil | Elatrous |
| Gülseren | Elay |
| Abubaker S | Elmaryul |
| Mohamed | Elsaadany |
| Hany | Elsayed |
| Samar | Elsayed |
| Malo | Emery |
| Sébastien | Ena |
| Kevin | Eng |
| Joshua A | Englert |
| Elif | Erdogan |
| Perihan | Ergin Ozcan |
| Ege | Eroglu |
| Miguel | Escobar |
| Figen | Esen |
| Arzu | Esen Tekeli |
| Alejandro | Esquivel |
| Helbert | Esquivel Gallegos |
| Hanane | Ezzouine |
| Alberto | Facchini |
| Mohammad | Faheem |
| Vito | Fanelli |
| Maria Fernanda | Farina |
| Muriel | Fartoukh |
| Lutz | Fehrle |
| Feng | Feng |
| Yufeng | Feng |
| Irene | Fernandez |
| Borja | Fernandez |
| Maria Lorena | Fernandez-Rodriguez |
| Carlos | Ferrando |
| Maria João | Ferreira da Silva |
| Mireia | Ferreruela |
| Janet | Ferrier |
| Matias Jesús | Flamm Zamorano |
| Laura | Flood |
| Leda | Floris |
| Martin | Fluckiger |
| Catalina | Forteza |
| Antonella | Fortunato |
| Eric | Frans |
| Antonella | Frattari |
| Sebastian | Fredes |
| Tim | Frenzel |
| Roberto | Fumagalli |
| Mariano Andres | Furche |
| Maurizio | Fusari |
| Edward | Fysh |
| Juan Luis | Galeas-Lopez |
| Louis-Marie | Galerneau |
| Analía | Garcia |
| María Fernanda | Garcia |
| Elisabet | Garcia |
| Pablo | Garcia Olivares |
| Jaroslaw | Garlicki |
| Aude | Garnero |
| Eugenio | Garofalo |
| Prabha | Gautam |
| Andrey | Gazenkampf |
| Stéphanie | Gelinotte |
| Domenico | Gelormini |
| Etienne | Ghrenassia |
| Angelo | Giacomucci |
| Robert | Giannoni |
| Andrea | Gigante |
| Nancy | Glober |
| Paolo | Gnesin |
| Yari | Gollo |
| Dina | Gomaa |
| Rosita | Gomero Paredes |
| Rui | Gomes |
| Raúl Alejandro | Gomez |
| Oscar | Gomez |
| Aroa | Gomez |
| Louise | Gondim |
| Manuel | Gonzalez |
| Isabel | Gonzalez |
| Alejandro | Gonzalez-Castro |
| Orlando | Gordillo Romero |
| Federico | Gordo |
| Philippe | Gouin |
| Jerónimo | Graf Santos |
| Rooney | Grainne |
| Matilde | Grando |
| Sanja | Granov Grabovica |
| Giacomo | Grasselli |
| Salvatore | Grasso |
| Rinaldo | Grasso |
| Lisa | Grimmer |
| Colin | Grissom |
| Alexey | Gritsan |
| Qing | Gu |
| Xiang-Dong | Guan |
| Fabio | Guarracino |
| Neus | Guasch |
| Luca | Guatteri |
| Renaud | Gueret |
| Claude | Guérin |
| Emmanuel | Guerot |
| Pierre-Gildas | Guitard |
| Fethi | Gül |
| Ayca | Gumus |
| Mohan | Gurjar |
| Patricia | Gutierrez |
| Abdelhamid | Hachimi |
| Adi | Hadzibegovic |
| Samantha | Hagan |
| Clare | Hammel |
| Joo | Han Song |
| Gabrielle | Hanlon |
| Seyed Mohammadreza | Hashemian |
| Serge | Heines |
| Johanna | Henriksson |
| Jean-Etienne | Herbrecht |
| Gabriel Omar | Heredia Orbegoso |
| Greet | Hermans |
| Andrew | Hermon |
| Rosana | Hernandez |
| Carmen | Hernandez |
| Luis | Herrera |
| Manuel | Herrera-Gutierrez |
| Leo | Heunks |
| Juan | Hidalgo |
| Dianne | Hill |
| Dagmar | Holmquist |
| Marcela | Homez |
| Xia | Hongtao |
| Anil | Hormis |
| Daniel | Horner |
| M Carmen | Hornos |
| Meihong | Hou |
| Stacy | House |
| Brahim | Housni |
| Keith | Hugill |
| Sally | Humphreys |
| Louis | Humbert |
| Stephanie | Hunter |
| Lee | Hwa Young |
| Nicolas | Iezzi |
| Santiago | Ilutovich |
| Volkan | Inal |
| Richard | Innes |
| Panagiotis | Ioannides |
| Giorgio Antonio | Iotti |
| Mariachiara | Ippolito |
| Hiromasa | Irie |
| Hiroki | Iriyama |
| Taiga | Itagaki |
| Javier | Izura |
| Santiago | Izza |
| Rakhshanda | Jabeen |
| Hamidreza | Jamaati |
| Sunil | Jamadarkhana |
| Amira | Jamoussi |
| Milosz | Jankowski |
| Luis Alberto | Jaramillo |
| Kyeongman | Jeon |
| Seok | Jeong Lee |
| Deepak | Jeswani |
| Simant | Jha |
| Liangyan | Jiang |
| Chen | Jing |
| Sébastien | Jochmans |
| Bror Anders | Johnstad |
| Lee | Jongmin |
| Aurélie | Joret |
| Bojan | Jovanovic |
| Detajin | Junhasavasdikul |
| Maria Teresa | Jurado |
| Elisa | Kam |
| Hidenobu | Kamohara |
| Caroline | Kane |
| Iskender | Kara |
| Sait | Karakurt |
| Cherdkiat | Karnjanarachata |
| Jun | Kataoka |
| Shinshu | Katayama |
| Shuchi | Kaushik |
| Nermin | Kelebek Girgin |
| Kathryn | Kerr |
| Ian | Kerslake |
| Prakash | Khairnar |
| Abidi | Khalid |
| Akram | Khan |
| Ashish K | Khanna |
| Reza | Khorasanee |
| Dieneke | Kienhorst |
| Cenk | Kirakli |
| Rihard | Knafelj |
| Mark Kol | Kol |
| Napplika | Kongpolprom |
| Csaba | Kopitko |
| Pervin | Korkmaz Ekren |
| Agnieszka | Kubisz-Pudelko |
| Zoltan | Kulcsar |
| Junji | Kumasawa |
| Kiyoyasu | Kurahashi |
| Akira | Kuriyama |
| Fernanda | Kutchak |
| Jon Henrik | Laake |
| Eduardo | Labarca |
| Françoise | Labat |
| César | Laborda |
| Manuel Alberto | Laca Barrera |
| Laurie | Lagache |
| Antonio | Landaverde Lopez |
| Michael | Lanspa |
| Valeria | Lascari |
| Matthieu | Le Meur |
| Su Hwan | Lee |
| Young Ju | Lee |
| Jinwoo | Lee |
| Won-Yeon | Lee |
| Jarone | Lee |
| Terje | Legernaes |
| Tamaas | Leiner |
| Virginie | Lemiale |
| Tiago | Leonor |
| Philipp M | Lepper |
| Dahuan | Li |
| Hongbin | Li |
| Oleg | Li |
| Ana Raquel | Lima |
| Dan | Lind |
| Edward | Litton |
| Ning | Liu |
| Ling | Liu |
| Jialin | Liu |
| Jean-François | Llitjos |
| Beatriz | Llorente |
| Rodolfo | Lopez |
| Claudia Elizabeth | Lopez |
| Claudia | Lopez Nava |
| Pablo | Lovazzano |
| Min | Lu |
| Francesca | Lucchese |
| Manuela | Lugano |
| Gustavo | Lugo Goytia |
| Hua | Luo |
| Ceri | Lynch |
| Sebastiano | Macheda |
| Victor Hugo | Madrigal Robles |
| Salvatore Maurizio | Maggiore |
| Mònica | Magret Iglesias |
| Peter | Malaga |
| Harish | Mallapura Maheswarappa |
| Guillermo | Malpartida |
| Andrey | Malyarchikov |
| Helena | Mansson |
| Anaid | Manzano |
| Ismael | Marey |
| Nathalie | Marin |
| Maria del Carmen | Marin |
| Eliana | Markman |
| Felix | Martin |
| Alex | Martin |
| Cristina | Martin Dal Gesso |
| Felipe | Martinez |
| Conchita | Martínez-Fidalgo |
| Ignacio | Martin-Loeches |
| Arantxa | Mas |
| Sakuraya | Masaaki |
| Emilio | Maseda |
| Eleni | Massa |
| Anna | Mattsson |
| Jessica | Maugeri |
| Victoria | McCredie |
| James | McCullough |
| Shay | McGuinness |
| Andrew | McKown |
| László | Medve |
| Chengqing | Mei |
| Ricard | Mellado Artigas |
| Vitor | Mendes |
| Mohamed Khalaf Ebraheim | Mervat |
| Isabelle | Michaux |
| Michael | Mikhaeil |
| Olga | Milagros |
| Igor | Milet |
| Maria Teresa | Millan |
| Zhang | Minwei |
| Lucia | Mirabella |
| Sanghamitra | Mishra |
| Giovanni | Mistraletti |
| Katsunori | Mochizuki |
| Onnen | Moerer |
| Arif | Moghal |
| Francesco | Mojoli |
| Alexandre | Molin |
| Zsolt | Molnar |
| Raquel | Montiel |
| Luca | Montini |
| Gianmario | Monza |
| Maria | Mora Aznar |
| Sunthiti | Morakul |
| Maria | Morales |
| Daniel | Moreno Torres |
| Diego Rolando | Morocho Tutillo |
| Catherine | Motherway |
| Doumiri | Mouhssine |
| Eleni | Mouloudi |
| Tapia | Muñoz |
| Carlos | Munoz de Cabo |
| Mohamed | Mustafa |
| Radhakrishnan | Muthuchellappan |
| Muraleekrishnan | Muthukrishnan |
| Stefano | Muttini |
| Isao | Nagata |
| Dick | Nahar |
| Misuzu | Nakanishi |
| Izumi | Nakayama |
| Silvio Antonio | Namendys-Silva |
| Rahul | Nanchal |
| Sivakumar | Nandakumar |
| Alessandra | Nasi |
| Kamal | Nasir |
| Paolo | Navalesi |
| Tayyba | Naz Aslam |
| Thuy | Nga Phan |
| Alistair | Nichol |
| Shuhei | Niiyama |
| Sofia | Nikolakopoulou |
| Elena | Nikolic |
| Kenichi | Nitta |
| Marko | Noc |
| Stephanie | Nonas |
| Saad | Nseir |
| Ayşe | Nur Soyturk |
| Yukako | Obata |
| Richard | Oeckler |
| Moe | Oguchi |
| Shinichiro | Ohshimo |
| Marina | Oikonomou |
| Agueda | Ojados |
| Maria Teresa | Oliveira |
| Wilson | Oliveira Filho |
| Carlo | Oliveri |
| Aitor | Olmos |
| Kazuya | Omura |
| Maria Cristina | Orlandi |
| Francesca | Orsenigo |
| Laura | Ortiz-Ruiz De Gordoa |
| Kei | Ota |
| Rainier | Ovalle Olmos |
| Nándo | Öveges |
| Peter | Oziemski |
| Ozlem | Ozkan Kuscu |
| Ezgi | Özyılmaz |
| Fernando | Pachas Alvarado |
| Gonzalo | Pagella |
| Vijayanand | Palaniswamy |
| Eugenio Luis | Palazon Sanchez |
| Salvatore | Palmese |
| Guojun | Pan |
| Wensen | Pan |
| Bernardo | Panka |
| Metaxia | Papanikolaou |
| Theonymfi | Papavasilopoulou |
| Ameet | Parekh |
| Rachael | Parke |
| Francisco J | Parrilla |
| Dácil | Parrilla |
| Taha | Pasha |
| Laura | Pasin |
| Luis | Patão |
| Mayur | Patel |
| Grisma | Patel |
| Basanta Kumar | Pati |
| Jayaprakash | Patil |
| Saroj | Pattnaik |
| Daniel | Paul |
| Maurizio | Pavesi |
| Vanesa Alejandra | Pavlotsky |
| Graciela | Paz |
| Enrique | Paz |
| Elisabetta | Pecci |
| Carlos | Pellegrini |
| Andrea Gabriela | Peña Padilla |
| Gaetano | Perchiazzi |
| Tiago | Pereira |
| Vera | Pereira |
| Manuel | Perez |
| Cesar | Perez Calvo |
| Meisy | Perez Cheng |
| Ronald | Perez Maita |
| Rodrigo | Pérez-Araos |
| Purificación | Perez-Teran |
| David | Perez-Torres |
| Gavin | Perkins |
| Paolo | Persona |
| Tananchai | Petnak |
| Marina | Petrova |
| Tai | Pham |
| François | Philippart |
| Edoardo | Picetti |
| Elisabetta | Pierucci |
| Edoardo | Piervincenzi |
| Riccardo | Pinciroli |
| Maria-Consuelo | Pintado |
| Lise | Piquilloud |
| Thomas | Piraino |
| Stephanie | Piras |
| Claudio | Piras |
| Pattarin | Pirompanich |
| Luigi | Pisani |
| Enrique | Platas |
| Gustavo | Plotnikow |
| Willy | Porras |
| Virginia | Porta |
| Mariana | Portilla |
| José | Portugal |
| Pedro | Povoa |
| Gwenael | Prat |
| Romina | Pratto |
| Gabriel | Preda |
| Isidro | Prieto |
| Estefania | Prol-Silva |
| Richard | Pugh |
| Yupeng | Qi |
| Chuanyun | Qian |
| Tiehe | Qin |
| Haibo | Qiu |
| Hongping | Qu |
| Teobaldo | Quintana |
| Rosari | Quispe Sierra |
| Rocio | Quispe Soto |
| Raihan | Rabbani |
| Mohamed | Rabee |
| Ahmed | Rabie |
| Maria Augusta | Rahe Pereira |
| Ashish | Rai |
| Sundar | Raj Ashok |
| Mostafa | Rajab |
| Navin | Ramdhani |
| Elizabeth | Ramey |
| Marco | Ranieri |
| Darshana | Rathod |
| Banambar | Ray |
| Shihan Mahmud | Redwanul Huq |
| Adrian | Regli |
| Rosa | Reina |
| Natalia | Resano Sarmiento |
| Faustine | Reynaud |
| Gemma | Rialp |
| Pilar | Ricart |
| Todd | Rice |
| Angus | Richardson |
| Marcelo | Rieder |
| Martin | Rinket |
| Fernando | Rios |
| Fernando | Rios |
| Alejandro | Risso Vazquez |
| Nuttapol | Rittayamai |
| Ivano | Riva |
| Monaly | Rivette |
| Oriol | Roca |
| Ferran | Roche-Campo |
| Covadonga | Rodriguez |
| Gabriel | Rodriguez |
| Daniel | Rodriguez Gonzalez |
| Xandra Yanina | Rodriguez Tucto |
| Angela | Rogers |
| María Elena | Romano |
| Linda | Rørtveit |
| Alastair | Rose |
| Damien | Roux |
| Anahita | Rouze |
| Paolo Nahuel | Rubatto Birri |
| Wang | Ruilan |
| Aldana | Ruiz Robledo |
| Antonio Luis | Ruiz-Aguilar |
| Tomohito | Sadahiro |
| Ignacio | Saez |
| Judith | Sagardia |
| Rajnish | Saha |
| Rohit | Saha |
| Narongkorn | Saiphoklang |
| Shigeki | Saito |
| Maie | Salem |
| Gabriele | Sales |
| Patricia | Salgado |
| Srinivas | Samavedam |
| Mhamed | Sami Mebazaa |
| Line | Samuelsson |
| Nandyelly | San Juan Roman |
| Patricia | Sanchez |
| Jesus | Sanchez-Ballesteros |
| Yazcitk | Sandoval |
| Emanuele | Sani |
| Martin | Santos |
| Carla | Santos |
| Masamitsu | Sanui |
| Lakshmikanthcharan | Saravanabavan |
| Sema | Sari |
| Agnes | Sarkany |
| Bertrand | Sauneuf |
| Monica | Savioli |
| Hilal | Sazak |
| Riccardo | Scano |
| Francis | Schneider |
| Frédérique | Schortgen |
| Marcus J | Schultz |
| Gabriele Leonie | Schwarz |
| Faruk | Seçkin Yücesoy |
| Andrew | Seely |
| Frederik | Seiler |
| Yasemin | Seker Tekdos |
| Kim | Seok Chan |
| Luca | Serano |
| Wojciech | Serednicki |
| Ary | Serpa Neto |
| Mariano | Setten |
| Asim | Shah |
| Bhagyesh | Shah |
| You | Shang |
| Pradeep | Shanmugasundaram |
| Konstantin | Shapovalov |
| Eman | Shebl |
| Takuya | Shiga |
| Nobuaki | Shime |
| Phil | Shin |
| Jack | Short |
| Chen | Shuhua |
| Sughrat | Siddiqui |
| Juan Ignacio | Silesky Jimenez |
| Daniel | Silva |
| Betania | Silva Sales |
| Koen | Simons |
| Brit Ågot | Sjøbø |
| David | Slessor |
| Jakub | Smiechowicz |
| Nathan | Smischney |
| Paul | Smith |
| Tim | Smith |
| Mark | Smith |
| Sarah | Snape |
| Lindi | Snyman |
| Filiep | Soetens |
| Kyung | Sook Hong |
| Miguel Ángel | Sosa Medellin |
| Giovanna | Soto |
| Xavier | Souloy |
| Elsa | Sousa |
| Stefania | Sovatzis |
| Didem | Sozutek |
| Savino | Spadaro |
| Marco | Spagnoli |
| Martin | Spångfors |
| Nick | Spittle |
| Mike | Spivey |
| Andrew | Stapleton |
| Branislava | Stefanovic |
| Lorraine | Stephenson |
| Elizabeth | Stevenson |
| Kristian | Strand |
| Maria Teresa | Strano |
| Slavenka | Straus |
| Chenliang | Sun |
| Rongqing | Sun |
| Venkat | Sundaram |
| Tai | SunPark |
| Elisabeth | Surlemont |
| Yuda | Sutherasan |
| Zsuzsanna | Szabo |
| Konstanty | Szuldrzynski |
| Christopher | Tainter |
| Akihiro | Takaba |
| Mandy | Tallott |
| Tamasato | Tamasato |
| Zhanhong | Tang |
| Viratch | Tangsujaritvijit |
| Leandro | Taniguchi |
| Daisuke | Taniguchi |
| Fabio | Tarantino |
| Krittika | Teerapuncharoen |
| Susana | Temprano |
| Pierpaolo | Terragni |
| Nicolas | Terzi |
| Anand | Thakur |
| Pongdhep | Theerawit |
| Arnaud W | Thille |
| Matt | Thomas |
| Poungrat | Thungtitigul |
| Martial | Thyrault |
| Nejla | Tilouch |
| Karina | Timenetsky |
| Juna | Tirapu |
| Manuel | Todeschini |
| Roser | Tomas |
| Christian | Tomaszewski |
| Tommaso | Tonetti |
| Alexandre | Tonnelier |
| John | Trinder |
| Konlawij | Trongtrakul |
| Jonathon | Truwit |
| Betty | Tsuei |
| Aiman | Tulaimat |
| Sema | Turan |
| Melda | Turkoglu |
| Sanjeev | Tyagi |
| Alejandro | Ubeda |
| Federica | Vagginelli |
| María Florencia | Valenti |
| Imma | Vallverdu |
| Alisha | Van Axel |
| Ingrid | van den Hul |
| Hans | van der Hoeven |
| Nardo | Van Der Meer |
| Frank | Van Haren |
| Marc | Vanhoof |
| Mónica | Vargas-Ordoñez |
| Rosanna | Vaschetto |
| Ettore | Vascotto |
| Maria | Vatsik |
| Ana | Vaz |
| Antonia | Vazquez-Sanchez |
| Sara | Ventura |
| Jan Wytze | Vermeijden |
| Anxela | Vidal |
| Jocyelle | Vieira |
| Bruno | Vilela Costa Pinto |
| Asisclo | Villagomez |
| Ana | Villagra |
| Cristina | Villegas Succar |
| Ole Georg | Vinorum |
| Giovanni | Vitale |
| Ramesh | Vj |
| Ana | Vochin |
| Guillaume | Voiriot |
| Carlo Alberto | Volta |
| Magnus | von Seth |
| Maazouzi | Wajdi |
| Don | Walsh |
| Shouhong | Wang |
| Gabriel | Wardi |
| Nils Christian | Ween-Velken |
| Bi-Lin | Wei |
| Dolf | Weller |
| Deborah | Welsh |
| Ingeborg | Welters |
| Michael | Wert |
| Simon | Whiteley |
| Elizabeth | Wilby |
| Erin | Williams |
| Karen | Williams |
| Antoinette | Wilson |
| Jadwiga | Wojtas |
| Jin | Won Huh |
| David | Wrathall |
| Christopher | Wright |
| Jian-Feng | Wu |
| Guo | Xi |
| Zheng-Jiang | Xing |
| Hongyang | Xu |
| Kotaro | Yamamoto |
| Jie | Yan |
| Julio | Yáñez |
| Xiaobo | Yang |
| Elliot | Yates |
| Ozlem | Yazicioglu Mocin |
| Zhenglong | Ye |
| Fatma | Yildirim |
| Norifumi | Yoshida |
| Hector Higo Leon | Yoshido |
| Bo | Young Lee |
| Rongguo | Yu |
| Gong | Yu |
| Tao | Yu |
| Boyun | Yuan |
| Nadwipa | Yuangtrakul |
| Tetsuya | Yumoto |
| Xie | Yun |
| Graciela | Zakalik |
| Ahmad | Zaki |
| Begoña | Zalba-Etayo |
| Massimo | Zambon |
| Bin | Zang |
| Gianluca | Zani |
| Jonathan | Zarka |
| Simone Maria | Zerbi |
| Avşar | Zerman |
| Harald | Zetterquist |
| Jiuzhi | Zhang |
| Hongwen | Zhang |
| Wei | Zhang |
| Guoxiu | Zhang |
| Weixin | Zhang |
| Hongsheng | Zhao |
| Jia | Zheng |
| Bin | Zhu |
| Ronald | Zumaran |

**Appendix 3: List Participating ICUs**

| 1. Unidad de Cuidados Intensivos, Sanatorio La Trinidad Mitre, Buenos Aires, Argentina |
| --- |
| 1. Unidad De Terapia Intensiva, Hospital D.F Santojanni, Buenos Aires, Argentina |
| 1. Unidad de Cuidados Intensivos, Sanatorio Anchorena, Buenos Aires, Argentina |
| 1. Unidad De Terapia Intensiva, Instituto De Investigaciones Medicas Alfredo Lanari, Buenos Aires, Argentina" |
| 1. Intensive Care Service, Otamendi Health Center, Buenos Aires, Argentina |
| 1. Intensive Care Unit, CEMIC, Buenos Aires, Argentina |
| 1. Intensive Care Unit, Bazterrica, Buenos Aires, Argentina |
| 1. Unidad de Terapia Intensiva de Adultos, Hospital Misericordia, Cordoba, Argentina |
| 1. Terapia Intensiva, Higa San Martin La Plata, La Plata, Argentina |
| 1. Terapia Intensiva, Instituto Medico Platense , La Plata, Argentina |
| 1. Terapia Intensiva Hospital Lagomaggiore, Hospital Luis Carlos Lagomaggiore, Mendoza, Argentina |
| 1. Terapia Intensiva Adultos, Hospital Nacional Profesor Alejandro Posadas, Moron, Argentina |
| 1. Unidad de Terapia Intensiva, Clinica San Agustín, Neuquén, Argentina |
| 1. Unidad de Terapia Intensiva, Hospital Provincial del Centenario, Rosario, Argentina |
| 1. Terapia Intensiva, Jose Maria Cullen, Santa Fe, Argentina |
| 1. Unidad de Cuidados Intensivos, Sanatorio Guemes, Buenos Aires, Argentina |
| 1. Unidad de cuidados intensivos, Clínica Santa Isabel, Buenos Aires, Argentina |
| 1. Terapia intensiva de quemados, Hospital Luis Carlos Lagomaggiore , Mendoza, Argentina |
| 1. Intensive Care, Hospital Central, Mendoza, Argentina |
| 1. UTI, Francisco Lopez Lima Hospital, General Roca, Argentina |
| 1. uti, Clinica y maternidad suizo argentina , Buenos Aires, Argentina |
| 1. Unidad de Terapia Intensiva, Sanatorio de Los Arcos, Buenos Aires, Argentina |
| 1. Intensive and Critical Care Unit, Flinders Medical Centre, Adelaide, Australia |
| 1. Critical Care Unit, Ballarat Health Services, Ballarat, Australia |
| 1. ICU, Canberra Hospital, Canberra, Australia |
| 1. Concord Hospital Intensive Care Unit, Concord Hospital, Concord, NSW, Australia |
| 1. Midland Intensive Care Unit, St John of God Midland Public and Private Hospitals, Perth, Australia |
| 1. Intensive Care Unit, Armadale Health Service, Perth, Australia |
| 1. ICU, Epworth Richmond, Richmond, Australia |
| 1. Intensive Care Unit, St John of God Subiaco, Perth, Australia |
| 1. Intensive Care Unit, John Hunter Hospital, Newcastle , Australia |
| 1. Intensive Care Unit, SJG Murdoch Hospital, WA Murdoch Perth, Australia |
| 1. Intensive Care, Gold Coast University Hospital, Southport, QLD, Australia |
| 1. Maroondah Intensive Care Unit, Eastern Health, Maroondah Campus, East Ringwood, Australia |
| 1. Intensive Care Unit, Cairns and Hinterland Hospital, Cairns City, Australia |
| 1. Medical Intensive Care Unite, UZLeuven, Leuven, Belgium |
| 1. ICU, AZ Turnhout, Turnhout, Belgium |
| 1. ICU, CHU UcL Namur site Godinne (Godinne University Hospital), Yvoir, Belgium |
| 1. Intensieve zorgen , Imelda Ziekenhuis, Bonheiden, Belgium |
| 1. Unidad de Terapia Intensiva, Hospital Universitario Japonés, Santa Cruz, Bolivia |
| 1. Department for cardioanesthesia , Clinic for cardiosurgery, University Clinical Center Kosevo, Sarajevo, Bosnia and Herzegovina |
| 1. centro de terapia intensiva da Santa Casa de Campo Grande, Associação beneficiente de Campo Grande MS, Campo Grande - Mato Grosso do Sul - MS, Brazil |
| 1. UTI, HPS 28 de agosto, Manaus, Brazil |
| 1. UTI do PSM, Hospital das Clínicas da FMUSP, Sao Paulo, Brazil |
| 1. ICU Hospital BP Mirante, BP Mirante, Sao Paulo, Brazil |
| 1. Critical Care Medicine, Hospital Israelita Albert Einstein, Sao Paulo, Brazil |
| 1. UTI Geral, Vitoria Apart Hospital, Vitoria, Brazil |
| 1. Unidade de terapia intensiva geral, Hospital \|Unimed Vitória, Vitoria, Brazil |
| 1. Unidade de Terapia Intensiva, Hospital Cristo Redentor , Porto Alegre, Brazil |
| 1. Medical Surgical intensive care, St Michael's hospital, Toronto, Canada |
| 1. ICU, The Ottawa Hospital, Ottawa, Canada |
| 1. Critical Care Unit, North York General Hospital, Toronto, Canada |
| 1. Medical Surgical Intensive Care Unit, London Health Sciences Centre - University Hospital, London, Canada |
| 1. MSNICU, Toronto Western Hospital, Division of University Health Network, Toronto, Canada |
| 1. The Allan T. Lambert Trauma and Neurosurgery Intensive Care Unit (TNICU), St. Michael's Hospital, Toronto, Canada |
| 1. Unidad de Cuidados Intensivos, Hospital Clinico Universidad de Chile, Santiago, Chile |
| 1. Unidad de Cuidados Intensivos Adulto, Hospital Felix Bulnes Cerda, Santiago, Chile |
| 1. Unidad de Cuidados Intensivos Generales, Hospital Naval Almirante Nef, Viña del Mar, Chile |
| 1. Unidad de Paciente Crítico , Hospital Clínico Pontificia Universidad Católica de Chile, Santiago, Chile |
| 1. DEPARTAMENTO DE PACIENTE CRITICO, CLINICA ALEMANA DE SANTIAGO, Santiago, Chile |
| 1. Department of Critical Care Medicine, BinZhu, ChangZhou, China |
| 1. ICU, the Fourth people`s hospital chang'zhou, Changzhou, China |
| 1. Department of Intensive Care Medicine, 1st affiliated Hospital of Dalian Medical university , Dalian, China |
| 1. Intensive Care Unit, Fujian Provincial Hospital, Fuzhou, China |
| 1. Intensive Care Unit, Guangdong General Hospital, Guangzhou, China |
| 1. Department of Surgical Intensive Care Unit, The First Affiliated Hospital, Sun Yat-sen University, Guangzhou, China |
| 1. Critical Care Medicine, Affiliated Hospital of Guiyang Medical University, Guiyang, China |
| 1. Jinxiang people's Hospital, jin xiang people's hospital, Jining City, China |
| 1. Emergency Department and EICU/MICU, First Affiliated Hospital of Kunming Medical University, Kunming, China |
| 1. Cardiovascular Surgery Intensive Care Unit, Kunming Medical University Affiliated Yan’an Hospital, Kunming, China |
| 1. Emergency Intensive Care Unit, THE FIRST AFFILIATED HOSPITAL OF HENAN UNIVERSITY OF SCIENCE & TECHNOLOGY, Luoyang, China |
| 1. intensive care unit, Nanjing Drum Tower Hospital, Nanjing, China |
| 1. Intensive Care Unit, Zhongda Hospital, Nanjing, China |
| 1. intensive care unit, Nanjing jiangbei people's hospital, Nanjing, China |
| 1. Sicu, First affiliated hospital of Guangxi Medical University, Nanning, China |
| 1. Intensive Care Unit, Affiliated Hospital of Nantong University, Nantong, China |
| 1. The Department of Critical Care Unit, Qilu hospital of Shandong University?Qingdao?, Qingdao, China |
| 1. Department of Critical Care Medicine,Ruijin Hospital, Shanghai Jiaotong University School of Medicine, Shanghai, China, Ruijin Hospital, Shanghai Jiaotong University School of Medicine, Shanghai, China, Shanghai, China |
| 1. ICU, Shanghai General hospital, Shanghai, China |
| 1. Intensive Care Unit, Sheng Jing Hospital of China Medical University, Shenyang, China |
| 1. RICU of the second Department of Respiration Medicine, The Second Hospital of Hebei Medical University, Shijiazhuang, China |
| 1. intensive care unit, Changshu No.1 People’ s Hospital, Soochow, China |
| 1. ICU, Union Hospital, Tong Ji Medical College, Huazhong University of Science and Technology, Wuhan, Hubei, China |
| 1. Department of critical care medicine, First Affiliated Hospital of Wannan Medical College, Yijishan Hospital, Wuhu, China |
| 1. ICU, WuXi people's hospital, WuXi, China |
| 1. Departement of Crit Care Unit, The First Affiliated Hospital of Xiamen University, Xiamen, China |
| 1. Critical care medicine, The First Affiliated Hospital of Zhengzhou University, Zhengzhou, China |
| 1. Unidad de Cuidados Intensivos , Fundación Valle del Lilí, Cali, Colombia |
| 1. Intensive Care Unit, Hospital Vicente Corral Moscoso, Cuenca, Ecuador |
| 1. UCI, Clinica la Merced, Quito, Ecuador |
| 1. Réanimation Polyvalente, Centre Hospitalier Pierre Oudot, Bourgoin-Jallieu, France |
| 1. réanimation médicale, CHU Cavale blanche, Brest , France |
| 1. Réanimation polyvalente, Hopital d'Instruction des Armées Clermont Tonnerre, Brest , France |
| 1. Service de Reanimation Médicale, Centre Hospitalo-Universitaire de Caen, Caen, France |
| 1. Réanimation Polyvalente, Centre Hospitalier Public du Cotentin, Cherbourg en Cotentin, France |
| 1. Réanimation médico-chirurgicale, Louis Mourier, Colombes, France |
| 1. Réanimation Adulte, Centre Hospitalier Intercommunal de Créteil, Créteil, France |
| 1. Réanimation Polyvalente, Centre Hospitalier de Dieppe, Dieppe, France |
| 1. Réanimation, Centre Hospitalier Universitaire Grenoble-Alpes, Grenoble , France |
| 1. Réanimation médico-chirurgicale et Unité de Surveillance Continue, Centre Hospitalier Le Mans, Le Mans, France |
| 1. Critical Care Center, CHU de Lille, Hôpital R. Salengro, Lille, France |
| 1. Réanimation Polyvalente, Centre Hospitalier des Deux Vallées - Site de Longjumeau, Longjumeau, France |
| 1. Réanimation Medicale, Croix Rousse, Lyon, France |
| 1. Réanimation - Médecine Intensive, Groupe Hospitalier Sud Ile de France - Site de Melun, Melun, France |
| 1. Réanimation médico-chirurgicale, CHU de Nice L'Archet 2, Nice, France |
| 1. Medical ICU, Medical ICU Archet 1 university Hospital, Nice, France |
| 1. Medical ICU, Saint Louis APHP, Paris, France |
| 1. Réanimation, Groupe Hospitalier Paris Saint-Joseph, Paris, France |
| 1. Réanimation Médicale, Hôpital Cochin, Paris, France |
| 1. Unité de Réanimation Médico-chirurgicale, Hôpital Tenon, Paris, France |
| 1. Reanimation médicale, Hopital Europeen Georges Pompidou, Paris, France |
| 1. servive de réanimation polyvalente et unité de soins-continus, centre hospitalier de cornouaille, Quimper, France |
| 1. Service de Réanimation , Centre Hospitalier, Roanne, France |
| 1. Réanimation Chirurgicale, CHU de Rouen, Rouen, France |
| 1. Medical intensive care unit, CHU de Rouen, Rouen, France |
| 1. Service de Médecine Intensive - Réanimation, Hôpital Delafontaine, Saint Denis, France |
| 1. SERVICE DE REANIMATION POLYVALENTE, CHU FELIX GUYON, SAINT-DENIS DE LA REUNION, France |
| 1. Réanimation médicale, Hopital de Hautepierre, Strasbourg, France |
| 1. service de réanimation, Sainte Musse Hospital, Toulon, France |
| 1. réanimation polyvalente, Hôpital Nord Franche Compte, Trévenans, France |
| 1. Unité de Réanimation, Service de Pneumologie et de Réanimation, Pitié Salpêtrière, Paris, France |
| 1. Médecine Intensive - Réanimation Médicale, CHU de Poitiers, Poitiers, France |
| 1. Reanimation polyvalente, Ghef site de jossigny, Jossigny, France |
| 1. Dept. of Internal Medicine V,, University Hospital of Saarland, Homburg, Germany |
| 1. Intensive Care Unit, Hippocration General Hospital of Athens, Athens, Greece |
| 1. ICU, General Hospital Katerini, Katerini, Greece |
| 1. ICU , IPPOKRATION General Hospital, Thessaloniki, Greece |
| 1. Intensive Care Unit, Asklepieion Voulas General Hospital, Voula Atikki, Greece |
| 1. B INTENSIVE CARE UNIT CLINIC, ATTIKON UNIVERSITY HOSPITAL, Athens, Greece |
| 1. Intensive Care Unit, Dr. Kenessey Albert Hospital, Balassagyarmat, Hungary |
| 1. ICU, Uzsoki Hospital, Budapest, Hungary |
| 1. Department of Anesthesiology and Intensive Therapy, University of Szeged, Szeged, Hungary |
| 1. ICU, St. George Hospital, Fejér County, Székesfehérvár, Hungary |
| 1. Anaesthesiologie, Universitaetsmedizin Goettingen, Goettingen, Germany |
| 1. EICU, CIMS HOSPITAL , Ahmedabad , India |
| 1. Department of Neuroanaesthesia and Neurocritical Care, National Institute of Mental Health and NeuroSciences, Bangalore , India |
| 1. Critical Care Unit, Apollo Hospitals Bhubaneswar, Bhubaneswar, India |
| 1. ICU , KOVAI MEDICAL CENTER AND HOSPITAL, Coimbatore, India |
| 1. MEDICAL ICU, PSRI - PUSHPAWATI SINGHANIA RESEARCH INSTITUTE, Delhi, India |
| 1. Critical Care Unit, Virinchi Hospital, Hyderabad, India |
| 1. Critical Care Medicine , Sanjay Gandhi Postgraduate Institute of Medical Sciences (SGPGIMS), Lucknow, India |
| 1. Critical Care , Sir H N Reliance Foundation Hospital, Mumbai, India |
| 1. ICU, CRITICARE HOSPITAL & RESEARCH INSTITUTE, Nagpur, India |
| 1. Intensive care unit, Metro heart institute with multispecialty, Faridabad, India |
| 1. Masih Daneshvari Critical Care unit, Masih Daneshvari (NRITLD), Tehran, Iran |
| 1. Intensive Care Unit, St Vincents University Hospital, Dublin, Ireland |
| 1. Intensive Care Unit, Galway University Hospital, Galway, Ireland |
| 1. Intensive Care Unit, Midland Regional Hospital, Mullingar, Ireland |
| 1. Intensive Care Unit, St James Hospital , Dublin, Ireland |
| 1. Intensive Care and Anaesthesia, University Hospital Limerick, Limerick, Ireland |
| 1. Anestesia e Rianimazione 3a - Terapia Intensiva Adulti, ASST Papa Giovanni XXIII - Bergamo, Bergamo, Italy |
| 1. U.O. Anestesiologia e Terapia Intensiva Polivalente e dei Trapianti, AOU di Bologna. Policlinico Sant'Orsola Malpighi, Bologna, Italy |
| 1. Seconda Rianimazione, Spedali Civili, Brescia , Italy |
| 1. Anestesia e Rianimazione, ASST-LARIANA presidio di Cantù, Cantù, Italy |
| 1. UOC ANESTESIA E RIANIMAZIONE \ANTONELLA CARUSO\", ARNAS GARIBALDI CATANIA, Catania, Italy" |
| 1. Rianimazione e Terapia Intensiva, Mater Domini, Catanzaro, Italy |
| 1. Terapia Intensiva, Ospedale Uboldo - Cernusco sul Naviglio, Cernusco sul Naviglio, Italy |
| 1. Intensive Care Unit, ASST Franciacorta, Chiari (Brescia), Italy |
| 1. Anesthesia and general ICU, SS. Annunziata, Chieti, Italy |
| 1. RIANIMAZIONE E TERAPIA INTENSIVA, BASSINI ASST NORD MILANO , Cinisello Balsamo-Milano, Italy |
| 1. Intensive care unit, Arcispedale Sant'Anna -Ferrara, Ferrara, Italy |
| 1. Terapia Intensiva Oncologica, AOU Careggi, Florence, Italy |
| 1. Intensive Care , Azienda OORR-University of Foggia, Foggia, Italy |
| 1. rianimazione e terapia intensiva, irccs San Martino ist genova, Genova, Italy |
| 1. Rianimazione generale, A.Manzoni, Lecco, Italy |
| 1. U.O. Rianimazione, Ospedale civile di Legnano, Legnano, Italy |
| 1. Reparto di Terapia Intensiva, ASST Santi Paolo e Carlo - Ospedale San Paolo - Polo Universitario, Milan, Italy |
| 1. UO Rianimazione Generale, Azienda Ospedaliera Fatebenefratelli Sacco - Ospedale Sacco - Polo Universitario, Milan, Italy |
| 1. Neurorianimazione, ASST Grande Ospedale Metropolitano Niguarda, Milan, Italy |
| 1. Terapia Intensiva Generale 1, ASST Grande Ospedale Metropolitano Niguarda, Milan, Italy |
| 1. U.O. Neurorianimazione - Terapia Intensiva, Ospedale Civile Sant'Agostino Estense, Modena, Italy |
| 1. Terapia Intensiva Generale, ASST Monza, Monza, Italy |
| 1. Rianimazione, Ospedale Maggiore della Carità, Novara, Italy |
| 1. SCDU Anestesia e Rianimazione, Azienda Ospedaliero-Universitaria S. Luigi Gonzaga, Orbassano (TO), Italy |
| 1. Terapia Intensiva, Policlinico San Marco Zingonia, Osio Sotto (Bergamo), Italy |
| 1. ISTAR2, Azienda Ospedaliera di Padova, Padova, Italy |
| 1. UOS Terapia intensiva , Ospedale Sant'Antonio, Padova, Italy |
| 1. UTIP, Policlinico Paolo Giaccone, Palermo, Italy |
| 1. Terapia Intensiva Polivalente con Trauma Center, ARNAS OSPEDALE CIVICO DI CRISTINA BENFRATELLI, Palermo, Italy |
| 1. Primo Servizio Anestesia e Rianimazione, AOU Ospedale Maggiore, Parma, Italy |
| 1. Rianimazione 1, Fondazione IRCCS Policlinico San Matteo, Pavia, Italy |
| 1. Intensive care unit, Ospedale Civile dello Spirito Santo, Pescara, Italy |
| 1. Rianimazione, Ospedale Santo Stefano Prato, Prato, Italy |
| 1. ANESTESIA E RIANIMAZIONE, S.MARIA DELLE CROCI, Ravenna, Italy |
| 1. Intensive care unit, Infermi, Rimini, Italy |
| 1. Rianimazione, Policlinico Umberto I - Sapienza Università di Roma, Rome, Italy |
| 1. Intensive Care Unit, Policlinico Universitario Agostino Gemelli, Rome, Italy |
| 1. Centro Rianimazione , San Giovanni Addolorata, Rome, Italy |
| 1. Terapia Intensiva Generale, Istituto Clinico Humanitas, Rozzano (Milano), Italy |
| 1. UOC Anestesia e Rianimazione, Azienda Ospedaliera Universitaria San Giovanni di Dio e Ruggi D'aragona, Salerno, Italy |
| 1. Rianimazione 2, Asst Lariana Ospedale S.Anna di Como, San Fermo della Battaglia, Italy |
| 1. Unità di Terapia Intensiva, AOU Sassari - Cliniche Universitarie, Sassari, Italy |
| 1. Terapia Intensiva, ASST Nord MIlano Ospedale Città di Sesto San Giovanni, Sesto San Giovanni, Milano, Italy |
| 1. Rianimazione e Medicina critica, AOU Senese, Siena , Italy |
| 1. Department of Anaesthesia and Intensive Care , Città della Salute e della Scienza di Torino, Turin, Italy |
| 1. Rianimazioneq, San Giovanni Bosco, Turin, Italy |
| 1. Clinica di Anestesia e Rianimazione, ASUIUD \S. Maria della Misericordia\", Udine, Italy" |
| 1. Anestesia e Rianimazione N°1, Azienda Sanitaria Universitaria Integrata di Udine , Udine, Italy |
| 1. Anestesia e Rianimazione A, AOUI Verona, Verona, Italy |
| 1. Intensive Care Unit \E. VECLA\", Fondazione IRCCS Ca' Granda Ospedale Maggiore Policlinico, Milan, Italy" |
| 1. S.C. Anestesia e Rianimazione 2, Azienda Ospedaliera di Perugia, Perugia, Italy |
| 1. Post-surgical intensive care, Fondazione IRCCS Policlinico San Matteo, Pavia, Italy |
| 1. UOC Terapia Intensiva e Anestesia, Grande Ospedale Metropolitano \Binchi-Melacrino-Morelli\", Reggio Calabria, Italy" |
| 1. Rianimazione, ASST Monza - Ospedale di Desio, Desio MB, Italy |
| 1. Rianimazione, ASST Bergamo Ovest Treviglio, Treviglio, Italy |
| 1. Anestesia Rianimazione 3, ASST Grande Ospedale Metropolitano Niguarda, Milan, Italy |
| 1. Intensive Care Unit, San Bortolo Hospital, Vicenza, Italy |
| 1. Terapia Intensiva 2, ASUIUD \Santa Maria della Misericordia\", Udine, Italy" |
| 1. ICU , Tokyo Women’s Medical University Yachiyo Medical Center, Chiba, Japan |
| 1. Advanced emergency and critical care center, Shinshu University Hospital, Matsumoto, Japan |
| 1. Emermgency Intensive Care Unit, Okayama University Hospital, Okayama, Japan |
| 1. ICU, Jichi Medical University Saitama Medical Center, Saitama, Japan |
| 1. Department of Critical Care Medicine, Sakai City Medical Center, Sakai City, Japan |
| 1. ICU, Tohoku University Hospital, Sendai, Japan |
| 1. Division of Intensive Care, Department of Anesthesiology and Intensive Care Medicine, Jichi Medical University School of Medicine, Shimotsuke, Tochigi, Japan |
| 1. ICU, Tokyo Bay Urayasu Ichikawa Medical Center, Urayasu, Japan |
| 1. Department of Emergency and Critical Care Medicine, Institute of Biomedical & Health Sciences, Hiroshima University Advanced Emergency and Critical Care Center, Hiroshima University Hospital , Hiroshima, Japan |
| 1. SICU, Kurume University Hospital, Kurume, Japan |
| 1. Department of Anesthesiology and Intensive Care, Hamamatsu University School of Medicine, Hamamatsu, Japan |
| 1. Intensive Care Unit, Kumamoto University Hospital, Kumamoto, Japan |
| 1. Intensive Care Unit, Jikei University hospital, Tokyo, Japan |
| 1. Emergensy and Critical care unit, Tokyo Medical Center, Tokyo, Japan |
| 1. Intensive Care Unit, Tokushima University Hospital, Tokushima, Japan |
| 1. Emergency Intensive Care Unit, Kurashiki Central Hospital, Okayama, Japan |
| 1. Department of Intensive and Intensive Care Medicine, JA Hiroshima General Hospital, Hatsukaichi city, Japan |
| 1. Department of Anesthesiology, Kurashiki Central Hospital, Kurashiki, Okayama, Japan |
| 1. Intensive Care Unit, Yokohama City Minato Red Cross Hospital, Yokohama, Japan |
| 1. ICU, Musashino Red Cross Hospital, Tokyo, Japan |
| 1. Intensive Care Unit, Okinawa Chubu Hospital, Uruma city, Okinawa, Japan |
| 1. micu, Tripoli Medical Center, Tripoli, Libya |
| 1. Areas Criticas, Hospital General de Ecatepec \Las Americas\", Ecatepec, Mexico" |
| 1. UNIDAD DE CUIDADOS INTENSIVOS ADULTOS, HOSPITAL CIVIL GUADALAJARA JUAN I MENCHACA, Guadalajara, Mexico |
| 1. Department of Critical Care Medicine, Fundación Clínica Médica Sur, Mexico city, Mexico |
| 1. Hospital General de Zona 48 San Pedro Xalpa, Hospital General de Zona 48, Mexico city, Mexico |
| 1. Unidad de Cuidados Intensivos, Hospital Regional 1 de Octubre, Mexico city, Mexico |
| 1. General Critical care unit, IMSS Hospital de Especialidades Antonio Fraga Mouret, Mexico city, Mexico |
| 1. Unidad de Cuidados Intensivos Respiratorios, Instituto Nacional de Enfermedades Respiratorias, Mexico city, Mexico |
| 1. Unidad de Cuidados Intensivos , IMSS Unidad Médica de Alta Especialidad No. 21, Monterrey, Mexico |
| 1. UCIA , HGR Clinica 72, Tlalnepantla , Mexico |
| 1. Service de Réanimation Médicale, Ibn Sina Hospital, Rabat, Morocco |
| 1. neurosurgical icu, Specialities hospital, Rabat, Morocco |
| 1. Medical ICU, Mohammed VIth University Hospital, Marrakech, Morocco |
| 1. Intensive Care Unit, GRANDE INTERNATIONAL HOSPITAL , Kathmandu, Nepal |
| 1. Intensive Care Unit, T U Teaching Hospital, Kathmandu, Nepal |
| 1. ICV, VU University Medical Centre Amsterdam, Amsterdam, Netherlands |
| 1. ICU, Academic Medical Center, Amsterdam, Netherlands |
| 1. Intensive Care Unit, Maastricht University Medical Centre+, Maastricht, Netherlands |
| 1. Intensive Care and Medium Care, Radboudumc, Nijmegen, Netherlands |
| 1. Intensive Care, Medisch Spectrum Twente, Enschede, Netherlands |
| 1. Intensive Care , Maasstad Ziekenhuis, Rotterdam, Netherlands |
| 1. Intensive Care Unit, Jeroen Bosch Ziekenhuis, 's-Hertogenbosch, Netherlands |
| 1. Cardiothoracic and Vascular ICU, Cardiothoracic and Vascular ICU, Auckland City Hospital, Auckland, New Zealand |
| 1. Intensive Care, Hutt Hospital, Lower Hutt, New Zealand |
| 1. Intensive Care Unit, Rotorua Hospital, Rotorua, New Zealand |
| 1. Intensive Care Unit, Wellington Hospital, Wellington, New Zealand |
| 1. Intensive Care Unit, Department for surgical services, Haukeland University Hospital, Bergen, Norway |
| 1. Intensive care unit (AKUM), Nordland Hospital (Nordlandssykehuset Boodø), Bodø, Norway |
| 1. Intensive care unit, Sykehuset Innlandet Hamar, Hamar, Norway |
| 1. Intensive care unit, Sorlandet Hospital, Kristiansand, Norway |
| 1. Intensive Care Unit, Akershus University Hospital, Lørenskog, Norway |
| 1. ICU , Sykehus Molde, Molde, Norway |
| 1. General Intensive Care Unit, Rikshospitalet Medical Centre, Oslo University Hospital, Oslo, Norway |
| 1. Intensive Care Unit 2M, Stavanger University Hospital, Stavanger, Norway |
| 1. Dept. Anaesthesia and Intensive care, Aalesund hospital, Aalesund, Norway |
| 1. Medical Intensive Care Unit/ Surgical Intensive Care Unit, Pakistan Institute Of Medical Sciences, Islamabad, Pakistan |
| 1. Surgical ICU, Pakistan Institute Of Medical Sciences, Islamabad, Pakistan |
| 1. Unidad de Cuidados Intensivos I, National Hospital Daniel Alcides CARRION, Bellavista District, Province Callao, Peru |
| 1. CRITICAL CARE UNIT, HOSPITAL NACIONAL ALBERTO SABOGAL SOLOGUREN, Callao, Peru |
| 1. AREA CRITICA, HOSPITAL REGIONAL LAMBAYEQUE, Chiclayo, Peru |
| 1. UNIDAD DE CUIDADOS INTENSIVOS, HOSPITAL REGIONAL ICA, Ica, Peru |
| 1. Unidad de Cuidados Intensivos I, Hospital Edgardo Rebagliati Martins, Lima, Peru |
| 1. Unidad de Cuidados Intensivos 2, Hospital Edgardo Rebagliati Martins, Lima, Peru |
| 1. Unidad de Cuidados Intensivos 3, Hospital Edgardo Rebagliati Martins, Lima, Peru |
| 1. DEPARTAMENTO DE MEDICINA CRITICA - UCI, INSTITUTO NACIONAL ENFERMEDADES NEOPLASICAS, Lima, Peru |
| 1. UNIDAD DE CUIDADOS INTENSIVOS, CLINICA ONCOSALUD-AUNA, Lima, Peru |
| 1. Servicio de cuidados criticos, Hospital Nacional Dos de Mayo, Lima, Peru |
| 1. Unidad de Cuidados Intensivos, Hospital de Emergencias Grau, Lima, Peru |
| 1. INTENSIVE CARE UNIT, CENTRO MEDICO NAVAL \CMST\", Lima, Peru" |
| 1. Servicio de Cuidados Críticos, Hospital Nacional Dos de Mayo, Lima, Peru |
| 1. Intensive care unit, Hospital de apoyo Santa Rosa, Lima, Peru |
| 1. UNIDAD DE CUIDADOS INTENSIVOS, HOSPITAL REGIONAL DE HUACHO, Huacho, Peru |
| 1. cuidados intensivos, Hospital de emergencias Jose Casimiro ULLOA, Miraflores, Peru |
| 1. Department of Anaesthesiology and Intensive Care, Uniwersyteckie Centrum Kliniczne SUM, Katowice, Poland |
| 1. Skawinska ICU, University Hospital in Krakow, Krakow, Poland |
| 1. 1st Anaesthesiology and Intensive Care Unit, University Hospital Krakow, Krakow, Poland |
| 1. Department of Anesthesiology and Intensive Therapy, Wroclaw University Hospital, Wroclaw, Poland |
| 1. Department of intensive care , University Hospital Krakow, Krakow, Poland |
| 1. Unidade de cuidados Intensivos, Centro Hospitalar Cova da Beira, Covilhã, Portugal |
| 1. Unidade de Urgência Médica, Centro Hospitalar Lisboa Central - Hospital de São José, Lisboa, Portugal |
| 1. Unidade de Cuidados Intensivos Polivalente, Hospital Sao Francisco Xavier, Lisboa, Portugal |
| 1. Intensive Care Department 2, Centro Hospitalar Universitário do Algarve, Portimão, Portugal |
| 1. Unidade de Cuidados Intensivos Polivalente, Hospital Santo Antonio, Porto, Portugal |
| 1. Serviço de Medicina Intensiva Polivalente, Centro Hospitalar Entre Douro & Vouga, Santa Maria da Feira, Portugal |
| 1. Serviço de Medicina Intensiva, Hospital Garcia de Orta, Almada, Portugal |
| 1. UCI Polivalente Geral, Hospital S.João, Porto, Portugal |
| 1. Unidade de Cuidados Intensivos I - SCI 1, Centro Hospitalar do Porto, Porto, Portugal |
| 1. Serviço Cuidados Intensivos - Unidade Marc Velge, Centro Hospitalar de Setúbal - Hospital de São Bernardo, Setúbal, Portugal |
| 1. Anaesthesiology and Intensive Care, Krasnoyarsk Clinical Regional Hospital, Krasnoyarsk, Russian Federation |
| 1. Department of Anesthesiology and Intensive care, Regional Clinical Hospital ?2, Vladivostok, Russian Federation |
| 1. ICU no. 2, Moscow State Clinical Hospital named after V.V.Vinogradova , Moscow, Russian Federation |
| 1. Icu, Saudi German Hospital , Jeddah, Saudi Arabia |
| 1. Intensive Care Service Dept. , Prince Sultan Military Medical City , Riyadh, Saudi Arabia |
| 1. Adult ICU (ICU-2), King Abdulaziz Medical City, Riyadh, Saudi Arabia |
| 1. ICU department, Care National Hospital, Riyadh , Saudi Arabia |
| 1. Intensive care units, AFHSR Armed Forced Hospital Southern Region, Khamis Mushyte, Saudi Arabia |
| 1. Trauma ICU, Clinical Center of Serbia Emergency center , Belgrade, Serbia |
| 1. SOK E, Clinical center of Serbia -Emergency center , Belgrade, Serbia |
| 1. Center for Internal Intensive Medicine, University Medical Center Ljubljana, Ljubljana, Slovenia |
| 1. Medical Intensive Care Unit, Seoul National University Bundang Hospital, Seongnam, South Korea |
| 1. ICU, Ewha Womans University Mokdong Hospital , Seoul, South Korea |
| 1. ICU, Seoul National University Hospital , Seoul, South Korea |
| 1. ICU, Samsung Medical Center, Seoul, South Korea |
| 1. Medical intensive care unit, Inje University Sanggye Paik Hospital, Seoul, South Korea |
| 1. ICU, Seoul St.Mary's Hospital, Seoul, South Korea |
| 1. MICU, Soon Chun Hyang University Hospital Seoul, Seoul, South Korea |
| 1. Department of Medical Intensive Care Unit, Severance Hospital, Yonsei University College of Medicine, Seoul, South Korea |
| 1. ICU, Uiheongbu St. Mary's Hospital, Uijeongbu-si, Gyeonggi-do, South Korea |
| 1. ICU, Wonju Severance Christian Hospital, Wonju, South Korea |
| 1. MICU, ASAN Medical Center, Seoul, South Korea |
| 1. SICU, Ewha Womans University Mokdong Hospital, Seoul, South Korea |
| 1. ICU, Hanyang University Guri Hospital, Guri-si, South Korea |
| 1. Intensive Care Unit, Hospital Punta de Europa, Algeciras, Spain |
| 1. Servicio de Medicina Intensiva, Hospital Universitari Germans Trias i Pujol, Badalona/Barcelona, Spain |
| 1. Unidad Cuidados Intensivos, CST Terrassa, Barcelona, Spain |
| 1. Intensive Care Unit, Hospital del Mar, Barcelona, Spain |
| 1. Unidad de Cuidados Intensivos , Hospital General Universitario de Ciudad Real, Ciudad Real, Spain |
| 1. UNIDAD DE CUIDADOS INTENSIVOS, Hospital Universitario del Henares, Coslada, Spain |
| 1. Servicio de Medicina Intensiva, Juan Ramón Jiménez , Huelva, Spain |
| 1. Critical Care Unit, H.G.U Gregorio Marañón, Madrid, Spain |
| 1. Department of Intensive Medicine, Hospital Universitario Ramon y Cajal, Madrid, Spain |
| 1. Unidad de Cuidados Intensiva.- Servicio de Medicina Intensiva, Hospital Universitario 12 de Octubre, Madrid, Spain |
| 1. UCI de Trauma y Emergencias, Hospital Universitario 12 de Octubre, Madrid, Spain |
| 1. ICU OF ANESTHESIA, Hospital Universitario LA PAZ, Madrid, Spain |
| 1. ICU, Hospital Regional Universitario de Malaga, Malaga, Spain |
| 1. UNIDAD DE CUIDADOS INTENSIVOS, Hospital Rey Juan Carlos, Mostoles (Madrid), Spain |
| 1. SERVICIO DE MEDICINA INTENSIVA (ICU), Hospital UNIVERSITARIO REINA SOFIA DE MURCIA, Murcia, Spain |
| 1. UNIDAD DE CUIDADOS INTENSIVOS, Hospital UNIVERSITARIO SON ESPASES, Palma de Mallorca, Spain |
| 1. Intensive Care Department, Hospital Son LLàtzer, Palma de Mallorca, Spain |
| 1. UCI, Complejo Hospitalario de Navarra, Pamplona Navarra, Spain |
| 1. UNIDAD DE CUIDADOS INTENSIVOS, HOSPITAL UNIVERSITARIO INFANTA CRISTINA, Parla (Madrid), Spain |
| 1. INTENSIVE CARE UNIT, Hospital Universitari Sant Joan, Reus, Spain |
| 1. UCI , Corporació Sanitària i Universitària Parc Taulí, Sabadell, Spain |
| 1. UCI, Hospital Clínico de Salmanca, Salamanca, Spain |
| 1. UCI, Hospital Universitario General de Catalunya, Sant Cugat del Valles, Spain |
| 1. Servei de Medicina Intensiva, Hospital Sant Joan Despí Moisès Broggi, Sant Joan Despí, Spain |
| 1. Critical Care Unit, Hospital Universitario Ntra Sra de Candelaria, Santa Cruz de Tenerife, Spain |
| 1. UCIG-A, Hospital Universitario Marqués de Valdecilla, Santander, Spain |
| 1. Intensive Care Unit, Hospital Joan XXIII, Tarragona, Spain |
| 1. Intensive Care Unit, Hospital Universitario de Torrejón, Torrejon de Ardoz, Spain |
| 1. Servei de medicina intensiva, Hospital Verge de la Cinta, Tortosa, Spain |
| 1. Unidad de Reanimación, Hospital Clínico Universitario of Valencia, Valencia, Spain |
| 1. TRAUMA-Quemados, Rio Hortega, Valladolid, Spain |
| 1. Polivalente, Rio Hortega, Valladolid, Spain |
| 1. Intensive Care Unit, HOSPITAL UNIVERSITARIO DE ALAVA, Vitoria-Gasteiz, Spain |
| 1. UCI Traumatología, Miguel Servet Universitary Hospital, Zaragoza, Spain |
| 1. Critical Care Unit, Universitary Clinic Lozano Blesa, Zaragoza, Spain |
| 1. Unidad de Cuidados Intensivos, Hospital de la Princesa, Madrid, Spain |
| 1. Adult Intensive care Unit, CHUV-University Hospital of Lausanne, Lausanne, Switzerland |
| 1. Unidad de Cuidados Intensivos, Hospital Universitario de Getafe, Getafe, Madrid, Spain |
| 1. UCI, Hospital Santa Bárbara de Soria, Soria, Spain |
| 1. Surgical Intensive Care Unit, Hospital del Mar - Parc de Salut , Barcelona , Spain |
| 1. Criticado café unit, Hospital Universitario Príncipe de Asturias, Alcalá de Henares, Spain |
| 1. INTENSIVE CARE UNIT, HOSPITAL GENERAL UNIVERSITARI DE CASTELLÓ, CASTELLO, Spain |
| 1. Servicio de Medicina Intensiva, Hospital de la Santa Creu i Sant Pau, Barcelona, Spain |
| 1. Unidad de Cuidados Intensivos del Hospital de Leon, Complejo asistencial Universitario de Leon, Leon, Spain |
| 1. Critical Care Department, Hospital Universitari Vall d'Hebron, Barcelona, Spain |
| 1. Intensive Care Unit, National Hospital for Paraplegics, Toledo, Spain |
| 1. Unidad de Cuidados Críticos, Hospital Universitario Fundación Alcorcón, Alcorcón, Spain |
| 1. Pulmonary and Critical Care Unit, King Chulalongkorn Memorial Hospital, Bangkok, Thailand |
| 1. Respiratory Intensive Care Unit, Faculty of Medicine Siriraj Hospital, Bangkok, Thailand |
| 1. 9IC Unit, Ramathibodi hospital , Bangkok, Thailand |
| 1. 8IK Unit, Ramathibodi hospital , Bangkok, Thailand |
| 1. 7NW Unit, Ramathibodi hospital , Bangkok, Thailand |
| 1. 3IC Unit, Ramathibodi, Bangkok, Thailand |
| 1. EMICU, Vajira Hospital, Bangkok, Thailand |
| 1. ICU, Thammasat University Hospital, Pathumthani, Thailand |
| 1. Medical ICU , Vajira hospital , Bangkok, Thailand |
| 1. Anesthesiology and ICU Department, Mongi Slim Hospital, La Marsa, La Marsa, Tunisia |
| 1. Service Réanimation Médicale, Taher Sfar Hospital Mahdia, Mahdia, Tunisia |
| 1. ?nternal medicine intensive care unit, Çukurova University Balcal? Hospital , Adana, Turkey |
| 1. Respiratory ICU, Cukurova University Hospital , Adana, Turkey |
| 1. Medical intensive care unit , Toros neighorhood, Adana , Turkey |
| 1. Intensive care unit, Türker High Aducation and Research Hospital, Ankara, Turkey |
| 1. Medical Intensive Care Unit 1 and 2, S.B.Ü Atatürk Chest Diseases and Thoracic Surgery Hospital SUAM, Ankara, Turkey |
| 1. Gazi University Hospital, Medical Intensive Care Unit, Gazi University Hospital, Ankara, Turkey |
| 1. General Surgical ?ntensive Care Unit, Ankara Diskapi Yildirim Beyazit Research and Education Hospital, Ankara, Turkey |
| 1. ANESTHESIOLOGY INTENSIVE CARE UNIT, Akdeniz University Hospital, Antalya, Turkey |
| 1. Chest Disease Department, Medicine School of Ege University, Bornova/IZMIR, Turkey |
| 1. Medical Intensive Care, Düzce University Hospital, Düzce, Turkey |
| 1. Medical Intensive Care Unit, Marmara University Hospital, Istanbul, Turkey |
| 1. Sadi Sun Intensive Care Unit, Istanbul University Cerrahpasa medical faculty, Istanbul, Turkey |
| 1. MEDICAL INTENS?VE CARE UNIT, Sureyyapasa Chest Diseases and Thoracic Surgery Training Hospital, Istanbul, Turkey |
| 1. Anesthesiology and Reanimation, Marmara University Hospital, Istanbul, Turkey |
| 1. ADULT INTENSIVE CARE UNIT, Sisli Etfal educational research hospital, Istanbul, Turkey |
| 1. Intensive Care Unit, Dr. Suat Seren Chest Diseases and Surgery Training Hospital, Izmir, Turkey |
| 1. General Intensive Care 1-2-3, Konya Numune Hastanesi, Konya, Turkey |
| 1. intensive care, Erciyes Unicersity Faculty of medicine department of internal critical care unit, Kayseri, Turkey |
| 1. Anesthesiology Intensive Care, University of Istanbul - Istanbul Medical Faculty, Istanbul, Turkey |
| 1. Intensive Care Unit, Stoke Mandeville Hospital, Aylesbury, Buckinghamshire, UK - England |
| 1. Barnsley Hospital Intensive Care Unit., Barnsley Hospital NHS Foundation Trust, Barnsley, UK - England |
| 1. Intensive Care Unit, Royal United Hospitals Bath NHS Foundation Trust, Bath, UK - England |
| 1. Critical Care Complex, Bedford Hospital NHS trust, Bedford, UK - England |
| 1. Intensive Care Unit, Heart of England NHS Foundation Trust, Birmingham, UK - England |
| 1. Intensive Care Unit, Royal Sussex County Hospital, Brighton, UK - England |
| 1. General Intensive Care Unit A600, Southmead Hospital, Bristol, UK - England |
| 1. Intensive Care Unit, Bristol Royal Infirmary, Bristol, UK - England |
| 1. ITU, Burton Hospitals NHS FT, Burton on Trent, UK - England |
| 1. Anaesthetics and Critical Care Unit, West Suffolk Hospital NHS Foundation Trust, Bury St Edmunds, UK - England |
| 1. ITU Cumberland Infirmary, NCUHTrust , Carlisle, UK - England |
| 1. Chesterfield Royal Department of Critical Care, Chesterfield Royal nhs foundation trust, Chesterfield, UK - England |
| 1. Critical Care Unit, Leighton Hospital, Crewe, UK - England |
| 1. Intensive Care Unit, Croydon Health Services NHS Trust, Croydon , UK - England |
| 1. Critical Care Unit, The Princess Alexandra Hospital NHS Trust, Harlow, UK - England |
| 1. ICU, Harrogate District Hospital NHS Foundation Trust, Harrogate, UK - England |
| 1. Critical Care Centre, North West Anglia NHS Foundation Trust Hinchingbrooke Hospital , Huntingdon , UK - England |
| 1. Intensive Care Unit (J54), St James's University Hosptial , Leeds, UK - England |
| 1. Intensive Care Unit, Royal Liverpool University Hospital, Liverpool, UK - England |
| 1. Christine Brown Intensive Care Unit, King's College Hospital, London, UK - England |
| 1. ITU, Hillingdon Hospital, London, UK - England |
| 1. , Salford Royal NHS Foundation Trust, Manchester, UK - England |
| 1. Department of Critical Care, Milton Keynes University Hospital, Milton Keynes, UK - England |
| 1. Critical Care Unit, The Rotherham NHS Foundation Trust, Rotherham, UK - England |
| 1. Intensive Care Unit, South Tyneside NHS Foundation Trust, South Shields, UK - England |
| 1. General Intensive Care Unit, Lister Hospital, Stevenage , UK - England |
| 1. Critical Care Unit, University Hospital North Tees, Stockton-on-Tees, UK - England |
| 1. Critical Care, Musgrove Park, Taunton, UK - England |
| 1. Intensive Care Unit, Torbay Hospital, Torquay, UK - England |
| 1. Critical Care, Royal Cornwall Hospital, Truro, UK - England |
| 1. Critical Care Unit, Pinderfields Hospital, Wakefield, UK - England |
| 1. Intesive Care , Ulster Hospital, Belfast, UK - Northern Ireland |
| 1. Intensive Care Unit, Dumfries and Galloway Royal Infirmary, Dumfries, UK - Scotland |
| 1. Intensive Care Unit, Queen Elizabeth University Hospital, Glasgow, Glasgow, UK - Scotland |
| 1. Intensive care unit, Royal Glamorgan Hospital, Llantrisant, UK - Wales |
| 1. Intensive Care Unit, Yeovil District Hospital, Yeovil, UK - England |
| 1. INTENSIVE CARE UNIT, GLAN CLWYD HOSPITAL, Rhyl, UK - Wales |
| 1. Intensive Care Unit, Royal Surrey County Hospital, Guildford Surrey, UK - England |
| 1. Intensive Care Unit, North Manchester General Hospital, Manchester, UK - England |
| 1. ICU, Guy's and St Thomas' Hospitals , London, UK - England |
| 1. Departamento de Medicina Intensiva, CASMU, Montevideo, Uruguay |
| 1. Catedra Medicina Intensiva, Hospital de Clinicas, Montevideo, Uruguay |
| 1. Blake 12 ICU, Massachussetts General Hospital, Boston, USA |
| 1. SICU, Beth Isreal Deaconess Medical Center, Boston, USA |
| 1. Medical Intesive Care Unit, John H Stroger Hospital of Cook County, Chicago, USA |
| 1. ICU, University of Cincinnati Medical Center , Cincinnati, Ohio, USA |
| 1. Surgical ( SICU +CVICU ) and Medical ICU , Cleveland Clinic Foundation, Cleveland , USA |
| 1. Division of Pulmonary and Critical Care Medicine, The Ohio State University Wexner Medical , Columbus, USA |
| 1. El Centro Regional Medical Center ICU, El Centro Regional Medical Center, El Centro, California, USA |
| 1. Medical, Surgical, Cardiac, Neuro, University of California San Diego - La Jolla campus, La Jolla, USA |
| 1. Sulpizio Cardiovascular Center ICU, UCSD Sulpizio, La Jolla, USA |
| 1. MICU, North Shore Medical Centre, Salem, USA |
| 1. Shock Trauma ICU and Respiratory ICU, Intermountain Medical Center, Murray, USA |
| 1. Medical Intensive Care Unit, Tulane Medical Center, New Orleans, USA |
| 1. Medical ICU, Oregon Health & Science University, Portland, USA |
| 1. Mary Brigh 7 D/E, Mayo Clinic Rochester, Rochester, USA |
| 1. MICU, Mayo Clinic St Marys Campus, Rochester, USA |
| 1. 10-3 and 10-4, Mayo Clinic, Methodist Campus, Rochester, USA |
| 1. Medical Intensive care unit, NSMC Union Hospital, Lynn, USA |
| 1. 10ICU, University of California San Diego Medical Center - Hillcrest, San Diego, USA |
| 1. Surgical Intensive Care, Medical Intensive Care Unit, Regions Hospital, St Paul, USA |
| 1. Medical ICU, Vanderbilt University Medical Center, Nashville, USA |
| 1. E2 ICU, Stanford , Stanford, USA |
| 1. Medical Intensive Care Unit, Froedtert and the Medical College of Wisconsin, Milwaukee, USA |
| 1. ICU, s' Lands Hospitaal Paramaribo, Paramaribo, Suriname |
| 1. ICU Diakonessenhuis, Diakonessenhuis, Paramaribo, Suriname |
| 1. Intensive Care, Academisch Ziekenhuis Paramaribo, Paramaribo, Suriname |
| 1. Intensive Care, Sint Vincentius Hospital, Paramaribo, Suriname |
| 1. Critical Care Medicine department, Alexandria University Hospital, Alexandria, Egypt |
| 1. ICU, Alexandria Main University Hospital, Alexandria, Egypt |
| 1. Respiratory ICU, Zagazig University Hospitals Chest Department , Zagazig, Egypt |
| 1. Critical Care Medicine Unit, Menoufia University Hospitals, Shibin El-kom, Egypt |
| 1. Intensive care unit, SQUARE HOSPITALS LTD, Dhaka , Bangladesh |
| 1. ICU, Kristianstad Hospital, Kristianstad, Sweden |
| 1. Centrala intensivvårdsavdelningen, Akademiska sjukhuset, Uppsala, Sweden |
| 1. Department of Anesthesilogy and Intesive Care, Sunderby Hospital, Luleå, Sweden |
| 1. IVA Västervikssjukhus, Västervikssjukhus, Västervik, Sweden |
| 1. Operationskliniken, Västmanlands Sjukhus Västerås, Västerås, Sweden |
| 1. IVA, Falun, Falu lasarett, Falun, Sweden |
| 1. Östersund Intensive Care, Östersunds Hospital, Östersund, Sweden |
| 1. IVA, Nykopings Lasarett, Nykoping, Sweden |
| 1. ICU, Hôpital Bernard Mevs, Port-Au-Prince, Haïti |
| 1. ICU - cardiac adult surgery, ACIBADEM CITY CLINIC TOKUDA HOSPIAL, Sofia, Bulgaria |

**Appendix 4: List endorsing Societies**

European Society of Intensive Care Medicine

European Respiratory Society

ANZICS Clinical Trials Group

Indian Society of Critical Care Medicine

Irish Critical Care Trials Group

Société de Réanimation de Langue Française (SRLF)

Réseau Européen de Recherche en Ventilation Artificielle (ReVA Network)

Société Française d’Anesthésie et de Réanimation (SFAR)

Society of Critical Care Medicine (Discovery Network)
