# Supplementary material for: Impact of frailty and older age on weaning from invasive ventilation: a secondary analysis of the WEAN SAFE study
Source: Ann Intensive Care. 2025 Jan 20;15:13. doi: 10.1186/s13613-025-01435-1 (PMC11743409; doi:10.1186/s13613-025-01435-1)
Supplement: Supplementary file 2 — Supplementary Material 2 [file 13613_2025_1435_MOESM2_ESM.docx]

**Figure e1:** **Impact of elderly/frail status on weaning process groups to Day 90.**

Kaplan-Meier analysis of impact of age/frailty group on likelihood of entering the weaning process, and of weaning success probability over time (***Panel B***) to Day 90.





**Figure e2: Weaning duration outcomes by age and frailty category.**

Stacked bar chart of impact of frailty status (***Panel A***) and older age (***Panel B***) on weaning outcomes in the study population (***Panel A***). The numbers at the top of each bar represent the number sof patients in each catefory.

**Table e1. Univariate (left, n = 957) and multivariable (right, n = 734) logistic regression models of factors associated with wean failure in the frail cohort**

|  | **Unadjusted Odds Ratio** | **95% CI** | **p** | **Adjusted Odds Ratio** | **95% CI** | **p** |
| --- | --- | --- | --- | --- | --- | --- |
| Age 80+ | 1.17 | 0.81, 1.68 | 0.39 | 1.35 | 0.87, 2.08 | 0.17 |
| Chronic Cardiac Failure | 0.80 | 0.53, 1.2 | 0.29 | 0.84 | 0.51, 1.37 | 0.50 |
| **Reason for ICU Admission** | | | | | | |
| Cardiac Arrest | 3.29 | 2.07, 5.21 | **< 0.01** | 2.67 | 1.51, 4.67 | **< 0.01** |
| Trauma | 0.74 | 0.11, 2.89 | 0.70 | 1.47 | 0.21, 6.6 | 0.65 |
| Neurologic (non-trauma | 1.01 | 0.64, 1.55 | 0.96 | 0.87 | 0.47, 1.55 | 0.65 |
| **Lung Injury Indices** | | | | | | |
| P/F ratio | 1.00 | 1, 1 | 0.10 | 1.00 | 1, 1 | 0.70 |
| Respiratory Rate | 1.06 | 1.03, 1.08 | **< 0.01** | 1.06 | 1.03, 1.09 | **< 0.01** |
| PEEP | 1.07 | 0.99, 1.16 | 0.08 | 1.06 | 0.97, 1.17 | 0.20 |
| Driving Pressure | 1.05 | 1.03, 1.07 | **< 0.01** | 1.04 | 1.01, 1.07 | **< 0.01** |
| SOFA score (non-neuro) | 1.03 | 1, 1.07 | **0.05** | 1.05 | 1, 1.11 | **0.04** |
| Immune compromised | 1.22 | 0.85, 1.74 | 0.27 | 1.49 | 0.96, 2.29 | 0.07 |
| Use of paralyzing medications | 1.66 | 0.85, 3.08 | 0.12 | 0.99 | 0.43, 2.14 | 0.99 |
| **Sedation on the first day fulfilling WEC (Reference: awake)** | | | | | | |
| Moderate sedation | 1.07 | 0.73, 1.57 | 0.73 | 0.98 | 0.62, 1.56 | 0.92 |
| Deep sedation | 2.35 | 1.57, 3.54 | **< 0.01** | 1.96 | 1.2, 3.23 | **0.01** |

**Table e2. Univariate (left, n = 563) and multivariable (right, n = 445) logistic regression models of factors associated with wean failure in the elderly cohort**

|  | **Unadjusted Odds Ratio** | **95% CI** | **p** | **Adjusted Odds Ratio** | **95% CI** | **p** |  |
| --- | --- | --- | --- | --- | --- | --- | --- |
| Frail | 1.46 | 0.96, 2.21 | 0.07 | 1.48 | 0.89, 2.47 | 0.13 |  |
| Chronic Cardiac Failure | 1.40 | 0.82, 2.32 | 0.21 | 1.29 | 0.68, 2.38 | 0.42 |  |
| **Reason for ICU Admission** | | | | | | | |
| Cardiac Arrest | 2.93 | 1.55, 5.46 | **< 0.01** | 2.70 | 1.26, 5.69 | **0.01** |  |
| Trauma | 1.40 | 0.53, 3.27 | 0.46 | 1.69 | 0.51, 4.81 | 0.35 |  |
| Neurologic (non-trauma) | 1.47 | 0.86, 2.45 | 0.14 | 1.46 | 0.73, 2.83 | 0.27 |  |
| **Lung Injury Indices** | | | | | | | |
| P/F ratio | 1.00 | 1, 1 | 0.09 | 1.00 | 1, 1 | 0.32 |  |
| Respiratory Rate | 1.03 | 1, 1.07 | **0.05** | 1.03 | 1, 1.08 | 0.08 |  |
| PEEP Settings | 1.13 | 1.01, 1.25 | **0.03** | 1.14 | 0.99, 1.3 | 0.06 |  |
| Driving Pressure | 1.05 | 1.01, 1.09 | **0.01** | 1.03 | 0.99, 1.08 | 0.15 |  |
| SOFA score (non-neuro) | 1.01 | 0.96, 1.06 | 0.77 | 0.97 | 0.91, 1.04 | 0.42 |  |
| Immune compromised | 1.18 | 0.61, 2.18 | 0.61 | 1.25 | 0.56, 2.62 | 0.58 |  |
| Use of paralyzing medications | 1.10 | 0.36, 2.85 | 0.85 | 0.98 | 0.29, 2.77 | 0.96 |  |
| **Sedation on the first day fulfilling WEC (Reference: awake)** | | | | | | | |
| Moderate sedation | 0.66 | 0.39, 1.1 | 0.11 | 0.65 | 0.36, 1.19 | 0.16 |  |
| Deep sedation | 1.46 | 0.87, 2.48 | 0.16 | 1.18 | 0.63, 2.24 | 0.60 |  |
