# Supplementary material for: Impact of frailty and older age on weaning from invasive ventilation: a secondary analysis of the WEAN SAFE study
Source: Ann Intensive Care. 2025 Jan 20;15:13. doi: 10.1186/s13613-025-01435-1 (PMC11743409; doi:10.1186/s13613-025-01435-1)
Supplement: Supplementary file 3 — Supplementary Material 3 [file 13613_2025_1435_MOESM3_ESM.docx]

**WEAN SAFE Steering Committee**

| **First name** | **Surname** |
| --- | --- |
| Gaëtan | Beduneau |
| Giacomo | Bellani |
| Laurent | Brochard |
| Ewan | Goligher |
| Giacomo | Grasselli |
| Leo | Heunks |
| John | Laffey |
| Fabiana | Madotto |
| Jordi | Mancebo |
| Antonio | Pesenti |
| Tai | Pham |
| Lise | Piquilloud |
| Hannah | Wunsch |
| Frank | van Haren |

**WEAN SAFE National Coordinators**

| **First name** | **Surname** |
| --- | --- |
| Elisa | Estenssoro |
| Frank | Van Haren |
| Greet | Hermans |
| Ary | Serpa Neto |
| Haibo | Qiu |
| Ewan | Goligher |
| Guillermo | Bugedo |
| Vladimir | Cerny |
| Assem Abdel | Razek |
| Gaëtan | Beduneau |
| Sébastien | Perbet |
| Onnen | Moerer |
| Dimitrios | Matamis |
| Alfred | Papali |
| Zsolt | Molnar |
| Pravin | Amin |
| Seyed Mohammadreza | Hashemian |
| Kevin | Clarkson |
| Giacomo | Grasselli |
| Kiyoyasu | Kurahashi |
| Subhash P | Acharya |
| Asisclo | Villagomez |
| Amine Ali | Zeggwagh |
| Leo M | Heunks |
| Jon Henrik | Laake |
| Rollin | Roldan |
| Konstanty | Szuldrzynski |
| Irene | Aragao |
| Dana | Tomescu |
| Alexey | Gritsan |
| Yaseen | Arabi |
| Bojan | Jovanovic |
| Young-Jae | Cho |
| Óscar | Peñuelas |
| Bernardo | Panka |
| Johan | Berkius |
| Lise | Piquilloud |
| Nuttapol | Rittayamai |
| Fekri | Abrough |
| Ezgi | Ozylmaz |
| Luigi | Camporota |
| Philippe | Bauer |
| Daniel | Talmor |
| Jeremy | Beitler |

**WEAN SAFE collaborators (by country, by city, by alphabetical order)**

| **First name** | **Surname** |
| --- | --- |
| Alma | Cani |
| Sebastian | Fredes |
| Santiago | Ilutovich |
| Marco | Bezzi |
| Silvina | Borello |
| Gustavo | Plotnikow |
| Romina | Pratto |
| Nicolas | Iezzi |
| Rodolfo | Lopez |
| Mariano Andres | Furche |
| Paolo Nahuel | Rubatto Birri |
| Pablo | Lovazzano |
| Mariano | Setten |
| Matilde | Grando |
| Vanesa Alejandra | Pavlotsky |
| Daniela | Benvenuti |
| Eliana | Markman |
| Graciela | Paz |
| Aldana | Ruiz Robledo |
| Raúl Alejandro | Gomez |
| María Florencia | Valenti |
| Anatilde | Diaz |
| Analía | Garcia |
| Rosana | Hernandez |
| Maria Cristina | Orlandi |
| Juan | Conde |
| Rosa | Reina |
| Gustavogcha | Chaparro |
| Gonzalo | Pagella |
| Graciela | Zakalik |
| Carlos | Pellegrini |
| Ariel | Chena |
| Maria Fernanda | Farina |
| Claudia Elizabeth | Lopez |
| Fernando | Rios |
| Judith | Sagardia |
| María Elena | Romano |
| Cristina | Villegas Succar |
| Lisandro Roberto | Bettini |
| Luis Pablo | Cardonnet |
| Fernando | Rios |
| Alejandro | Risso Vazquez |
| Ramón | Carrillo |
| Robert | Giannoni |
| Gabriela | Bai |
| Santiago | Izza |
| Miguel | Escobar |
| Patricia | Sanchez |
| Andrew | Bersten |
| Shailesh | Bihari |
| Dianne | Hill |
| Angus | Richardson |
| Graeme | Duke |
| Stephanie | Hunter |
| Bernie | Bissett |
| Frank | Van Haren |
| Mark Kol | Kol |
| Asim | Shah |
| Peter | Oziemski |
| Deborah | Welsh |
| Vijayanand | Palaniswamy |
| Kathryn | Kerr |
| Ameet | Parekh |
| Ege | Eroglu |
| Adrian | Regli |
| Edward | Fysh |
| David | Blythe |
| Muraleekrishnan | Muthukrishnan |
| Janet | Ferrier |
| Edward | Litton |
| Gabrielle | Hanlon |
| Jonathan | Barrett |
| James | McCullough |
| Mandy | Tallott |
| Shihan Mahmud | Redwanul Huq |
| Raihan | Rabbani |
| Eric | Frans |
| Helga | Ceunen |
| Greet | Hermans |
| Filiep | Soetens |
| Marc | Vanhoof |
| Pierre | Bulpa |
| Isabelle | Michaux |
| Mónica | Crespo Ramirez |
| Orlando | Gordillo Romero |
| Sanja | Granov Grabovica |
| Slavenka | Straus |
| Bruno | Vilela Costa Pinto |
| Maria Augusta | Rahe Pereira |
| Tamasato | Tamasato |
| Wilson | Oliveira Filho |
| Jocyelle | Vieira |
| Fernanda | Kutchak |
| Marcelo | Rieder |
| Fabianne | Dantas |
| Louise | Gondim |
| Luciano | Azevedo |
| Leandro | Taniguchi |
| Fernando | da Silva Ramos |
| Ary | Serpa Neto |
| Karina | Timenetsky |
| Stephanie | Piras |
| Claudio | Piras |
| Eliana | Caser |
| Betania | Silva Sales |
| Margarita | Borislavova |
| Karen J | Bosma |
| Michael | Mikhaeil |
| Andrew | Seely |
| Laurent | Brochard |
| Tai | Pham |
| Ricard | Mellado Artigas |
| Thomas | Piraino |
| Phil | Shin |
| Sharique | Ansari |
| Victoria | McCredie |
| Daniel | Arellano |
| Rodrigo | Cornejo |
| Matias Jesús | Flamm Zamorano |
| Manuel | Gonzalez |
| Guillermo | Bugedo |
| David | Carpio |
| Jerónimo | Graf Santos |
| Rodrigo | Pérez-Araos |
| Eduardo | Labarca |
| Felipe | Martinez |
| Bin | Zhu |
| Guojun | Pan |
| Chen | Shuhua |
| Jiuzhi | Zhang |
| Kai | Chen |
| Rongguo | Yu |
| Tiehe | Qin |
| Shouhong | Wang |
| Xiang-Dong | Guan |
| Jian-Feng | Wu |
| Bi-Lin | Wei |
| Feng | Feng |
| Meihong | Hou |
| Hongwen | Zhang |
| Chuanyun | Qian |
| Wei | Zhang |
| Jia | Zheng |
| Zheng-Jiang | Xing |
| Dahuan | Li |
| Guoxiu | Zhang |
| Qing | Gu |
| Ning | Liu |
| Ling | Liu |
| Haibo | Qiu |
| Chengqing | Mei |
| Zhenglong | Ye |
| Liangyan | Jiang |
| Zhanhong | Tang |
| Chenliang | Sun |
| Hongsheng | Zhao |
| Wu | Dawei |
| Guo | Xi |
| Jialin | Liu |
| Hongping | Qu |
| Wang | Ruilan |
| Xie | Yun |
| Bin | Zang |
| Hua | Luo |
| Weixin | Zhang |
| Wensen | Pan |
| Boyun | Yuan |
| Yufeng | Feng |
| Min | Lu |
| Xia | Hongtao |
| Gong | Yu |
| You | Shang |
| Xiaobo | Yang |
| Yupeng | Qi |
| Tao | Yu |
| Hongyang | Xu |
| Jie | Yan |
| Chen | Jing |
| Zhang | Minwei |
| Hongbin | Li |
| Rongqing | Sun |
| Mónica | Vargas-Ordoñez |
| Juan Ignacio | Silesky Jimenez |
| Hernan | Aguirre-Bermeo |
| Diego Rolando | Morocho Tutillo |
| Andrea Gabriela | Peña Padilla |
| Diana | Alvarez |
| María Fernanda | Garcia |
| Mohamed | Elsaadany |
| Hany | Elsayed |
| Samar | Elsayed |
| Abdelrhman | Aboshady |
| Nagwa | Doha |
| Eman | Shebl |
| Philippe | Crova |
| Thuy | Nga Phan |
| Simon | Bocher |
| Gwenael | Prat |
| Marc | Danguy des Déserts |
| Françoise | Labat |
| Cédric | Daubin |
| Aurélie | Joret |
| Bertrand | Sauneuf |
| Xavier | Souloy |
| Malo | Emery |
| Damien | Roux |
| Frédérique | Schortgen |
| Pierre-Louis | Declercq |
| Stéphanie | Gelinotte |
| Louis-Marie | Galerneau |
| Nicolas | Terzi |
| Frank | Chemouni |
| Jonathan | Zarka |
| Nicolas | Chudeau |
| Saad | Nseir |
| Anahita | Rouze |
| Matthieu | Le Meur |
| Martial | Thyrault |
| Claude | Guérin |
| Jonathan | Chelly |
| Sébastien | Jochmans |
| Pierre-Eric | Danin |
| Jean | Dellamonica |
| Alexandre | Robert |
| Virginie | Lemiale |
| Cédric | Bruel |
| François | Philippart |
| Jean-François | Llitjos |
| Nathalie | Marin |
| Muriel | Fartoukh |
| Guillaume | Voiriot |
| Emmanuel | Guerot |
| Maxens | Decavèle |
| Martin | Dres |
| Faustine | Reynaud |
| Arnaud W | Thille |
| Alexandre | Tonnelier |
| Pascal | Beuret |
| Sébastien | Ena |
| Philippe | Gouin |
| Pierre-Gildas | Guitard |
| Gaëtan | Beduneau |
| Elisabeth | Surlemont |
| Gabriel | Preda |
| Daniel | Silva |
| Laurence | Dangers |
| Jean-Etienne | Herbrecht |
| Francis | Schneider |
| Jean-Michel | Arnal |
| Aude | Garnero |
| Julio | Badie |
| Loïc | Barrot |
| Onnen | Moerer |
| Philipp M | Lepper |
| Frederik | Seiler |
| Metaxia | Papanikolaou |
| Theonymfi | Papavasilopoulou |
| Olympia | Apostolopoulou |
| Chrysi | Diakaki |
| Panagiotis | Ioannides |
| Marina | Oikonomou |
| Eleni | Massa |
| Eleni | Mouloudi |
| Aikaterini | Dimoula |
| Sofia | Nikolakopoulou |
| Stacy | House |
| Monaly | Rivette |
| Csaba | Kopitko |
| László | Medve |
| Zoltan | Kulcsar |
| Zsuzsanna | Szabo |
| Zsolt | Molnar |
| Nándo | Öveges |
| Agnes | Sarkany |
| Shuchi | Kaushik |
| Bhagyesh | Shah |
| Radhakrishnan | Muthuchellappan |
| Ramesh | Vj |
| Saroj | Pattnaik |
| Banambar | Ray |
| Sanghamitra | Mishra |
| Basanta Kumar | Pati |
| Sivakumar | Nandakumar |
| Lakshmikanthcharan | Saravanabavan |
| Lakshay | Bhakhtiani |
| Simant | Jha |
| Vijay Kumar | Agrawal |
| Prakash | Khairnar |
| Srinivas | Samavedam |
| Arvind | Baronia |
| Mohan | Gurjar |
| Mayur | Patel |
| Darshana | Rathod |
| Harshal | Bawangade |
| Deepak | Jeswani |
| Harish | Mallapura Maheswarappa |
| Seyed Mohammadreza | Hashemian |
| Hamidreza | Jamaati |
| Laura | Flood |
| Alistair | Nichol |
| Ignacio | Martin-Loeches |
| Lindi | Snyman |
| Kevin | Clarkson |
| Rooney | Grainne |
| Catherine | Motherway |
| Don | Walsh |
| Mohammad | Faheem |
| Salvatore | Grasso |
| Rossella | di Mussi |
| Alessandra | Nasi |
| Ivano | Riva |
| Elisabetta | Pierucci |
| Rocco | D’Andrea |
| Elisabetta | Pecci |
| Rinaldo | Grasso |
| Gianmario | Monza |
| Jessica | Maugeri |
| Agrippino | Bellissima |
| Eugenio | Garofalo |
| Paolo | Navalesi |
| Massimo | Zambon |
| Paolo | Gnesin |
| Manuel | Todeschini |
| Salvatore Maurizio | Maggiore |
| Luca | Serano |
| Stefano | Muttini |
| Eduardo | Beck |
| Alberto | Facchini |
| Luca | Guatteri |
| Savino | Spadaro |
| Carlo Alberto | Volta |
| Cosimo | Chelazzi |
| Gilda | Cinnella |
| Lucia | Mirabella |
| Alexandre | Molin |
| Fabio | Tarantino |
| Andrea | Coppadoro |
| Ettore | Vascotto |
| Francesca | Orsenigo |
| Virginia | Porta |
| Davide | Chiumello |
| Giovanni | Mistraletti |
| Antonio | Castelli |
| Riccardo | Colombo |
| Francesco | Curto |
| Roberto | Fumagalli |
| Riccardo | Pinciroli |
| Giacomo | Grasselli |
| Monica | Savioli |
| Maurizio | Bottiroli |
| Maurizio | Pavesi |
| Giacomo | Bellani |
| Carlo | Oliveri |
| Rosanna | Vaschetto |
| Pietro | Caironi |
| Giacomo | Berta |
| Bruno | Ballico |
| Giovanni | Vitale |
| Paolo | Persona |
| Tommaso | Tonetti |
| Sabrina | Boraso |
| Laura | Pasin |
| Andrea | Cortegiani |
| Mariachiara | Ippolito |
| Andrea Neville | Cracchiolo |
| Maria Teresa | Strano |
| Edoardo | Picetti |
| Emanuele | Sani |
| Mirko | Belliato |
| Giorgio Antonio | Iotti |
| Anna | Aliberti |
| Francesco | Mojoli |
| Angelo | Giacomucci |
| Antonella | Frattari |
| Pietro | Bertini |
| Fabio | Guarracino |
| Iacopo | Cappellini |
| Guglielmo | Consales |
| Maurizio | Fusari |
| Gianluca | Zani |
| Andrea | Bruni |
| Sebastiano | Macheda |
| Laura | Bernabe |
| Edoardo | Piervincenzi |
| Marco | Ranieri |
| Gennaro | De Pascale |
| Luca | Montini |
| Roberta | Caccese |
| Yari | Gollo |
| Valeria | Lascari |
| Antonella | Fortunato |
| Salvatore | Palmese |
| Marco | Spagnoli |
| Simone Maria | Zerbi |
| Leda | Floris |
| Pierpaolo | Terragni |
| Stefano | Clementi |
| Rosella | Barbieri |
| Lucia | Cubattoli |
| Vito | Fanelli |
| Gabriele | Sales |
| Stefania | Sovatzis |
| Massimo | Borelli |
| Federica | Vagginelli |
| Paolo | Chiarandini |
| Manuela | Lugano |
| Stefania | Buttera |
| Andrea | Gigante |
| Francesca | Lucchese |
| Domenico | Gelormini |
| Elisa | Boni |
| Silvia | De Rosa |
| Moe | Oguchi |
| Tomohito | Sadahiro |
| Yukako | Obata |
| Sakuraya | Masaaki |
| Akihiro | Takaba |
| Shinichiro | Ohshimo |
| Nobuaki | Shime |
| Hidenobu | Kamohara |
| Hiromasa | Irie |
| Koichi | Arinaga |
| Shuhei | Niiyama |
| Katsunori | Mochizuki |
| Kenichi | Nitta |
| Tetsuya | Yumoto |
| Akira | Kuriyama |
| Misuzu | Nakanishi |
| Masamitsu | Sanui |
| Junji | Kumasawa |
| Takuya | Shiga |
| Norifumi | Yoshida |
| Shinshu | Katayama |
| Taiga | Itagaki |
| Kiyoyasu | Kurahashi |
| Kazuya | Omura |
| Kengo | Asano |
| Kei | Ota |
| Kotaro | Yamamoto |
| Daisuke | Taniguchi |
| Jun | Kataoka |
| Hiroki | Iriyama |
| Toshikazu | Abe |
| Izumi | Nakayama |
| Isao | Nagata |
| Mohamed | Benlamin |
| Abubaker S | Elmaryul |
| Felipe | de Jesus Montelongo |
| Victor Hugo | Madrigal Robles |
| Daniel | Rodriguez Gonzalez |
| Silvio Antonio | Namendys-Silva |
| Claudia | Lopez Nava |
| Nandyelly | San Juan Roman |
| Maria del Carmen | Marin |
| Asisclo | Villagomez |
| Nancy | Canedo |
| Alejandro | Esquivel |
| Carmen | Hernandez |
| Gustavo | Lugo Goytia |
| Antonio | Landaverde Lopez |
| Miguel Ángel | Sosa Medellin |
| Anaid | Manzano |
| Abdellatif | Benslama |
| Hanane | Ezzouine |
| Abdelhamid | Hachimi |
| Brahim | Housni |
| Tarek | Dendane |
| Abidi | Khalid |
| Doumiri | Mouhssine |
| Maazouzi | Wajdi |
| Subhash | Acharya |
| Anand | Thakur |
| Prabha | Gautam |
| Leo | Heunks |
| Ingrid | van den Hul |
| Luigi | Pisani |
| Marcus J | Schultz |
| Martin | Rinket |
| Jan Wytze | Vermeijden |
| Melanie | Acampo-de Jong |
| Serge | Heines |
| Tim | Frenzel |
| Hans | van der Hoeven |
| Nardo | Van Der Meer |
| Dolf | Weller |
| Koen | Simons |
| Rachael | Parke |
| Shay | McGuinness |
| Carmel | Chapman |
| Andrew | Stapleton |
| Ulrike | Buehner |
| Erin | Williams |
| Nina | Beehre |
| Finn H | Andersen |
| Brit Ågot | Sjøbø |
| Gabriele Leonie | Schwarz |
| Knut | Dybwik |
| Bror Anders | Johnstad |
| Terje | Legernaes |
| Ole Georg | Vinorum |
| Nils Christian | Ween-Velken |
| Martin | Fluckiger |
| Lutz | Fehrle |
| Tayyba | Naz Aslam |
| Jon Henrik | Laake |
| Linda | Rørtveit |
| Kristian | Strand |
| Muneeb | Ali |
| Taha | Pasha |
| Rakhshanda | Jabeen |
| Kamal | Nasir |
| Cecilia Eugenia | Chavez |
| Patricia | Gutierrez |
| Tapia | Muñoz |
| Jorge | Cabrera |
| Willy | Porras |
| Luis | Coaguila |
| Giovanna | Soto |
| Rosita | Gomero Paredes |
| Martin | Santos |
| Jesus Milagrito | Avalos Cabrera |
| Ivan | Canchos Gutierrez |
| Hector Higo Leon | Yoshido |
| Ronald | Zumaran |
| Guillermo | Malpartida |
| José | Portugal |
| Gabriel Omar | Heredia Orbegoso |
| Xandra Yanina | Rodriguez Tucto |
| Ronald | Perez Maita |
| Rocio | Quispe Soto |
| Helbert | Esquivel Gallegos |
| José | Cruz |
| Enrique | Paz |
| Willy | Diaz |
| Oscar | Gomez |
| Rainier | Ovalle Olmos |
| Rosari | Quispe Sierra |
| Peter | Malaga |
| Yazcitk | Sandoval |
| Manuel Alberto | Laca Barrera |
| Fernando | Pachas Alvarado |
| Teobaldo | Quintana |
| Julio | Yáñez |
| Luis | Herrera |
| Olga | Milagros |
| Mestanza | Arica |
| Piotr | Czempik |
| Milosz | Jankowski |
| Konstanty | Szuldrzynski |
| Jaroslaw | Garlicki |
| Wojciech | Serednicki |
| Jadwiga | Wojtas |
| Jakub | Smiechowicz |
| Nuno | Catorze |
| Tiago | Pereira |
| Rui | Gomes |
| Vera | Pereira |
| Cristina | Coxo |
| Luis | Bento |
| Sara | Ventura |
| Vitor | Mendes |
| Pedro | Povoa |
| Maksym | Dykyy |
| Juan | Hidalgo |
| Maria Teresa | Oliveira |
| Ana | Vaz |
| Heloisa | Castro |
| Maria João | Ferreira da Silva |
| Tiago | Leonor |
| Elsa | Sousa |
| João | Carvalho |
| Guilherme | Domingos |
| Ana Raquel | Lima |
| Igor | Milet |
| Luis | Patão |
| Carla | Santos |
| Andrey | Malyarchikov |
| Konstantin | Shapovalov |
| Andrey | Gazenkampf |
| Alexey | Gritsan |
| Marina | Petrova |
| Maria | Vatsik |
| Pavel | Dunts |
| Oleg | Li |
| Maie | Salem |
| Ghamdan | Al Sadeh |
| Mohamed | Mustafa |
| Yaseen | Arabi |
| Sultan | Alamri |
| Mohamed | Rabee |
| Ahmed | Rabie |
| Mostafa | Rajab |
| Mohamed Khalaf Ebraheim | Mervat |
| Ismael | Marey |
| Adi | Hadzibegovic |
| Bojan | Jovanovic |
| Branislava | Stefanovic |
| Rihard | Knafelj |
| Marko | Noc |
| Tai | SunPark |
| Young-Jae | Cho |
| Su Hwan | Lee |
| Young Ju | Lee |
| Kyung | Sook Hong |
| Jinwoo | Lee |
| Kyeongman | Jeon |
| Youjin | Chang |
| Lee | Jongmin |
| Kim | Seok Chan |
| Bo | Young Lee |
| Joo | Han Song |
| Jin | Won Huh |
| Lee | Hwa Young |
| Seok | Jeong Lee |
| Won-Yeon | Lee |
| Beatriz | Llorente |
| Maria-Consuelo | Pintado |
| Irene | Fernandez |
| Alejandro | Ubeda |
| María | del Carmen Campos Moreno |
| Cristina | Martin Dal Gesso |
| Aroa | Gomez |
| Pilar | Ricart |
| Joaquin | Amador |
| Maria Teresa | Jurado |
| Purificación | Perez-Teran |
| Antonia | Vazquez-Sanchez |
| Adela | Benitez-Cano |
| Jesus | Carazo |
| Francisco J. | Parrilla |
| César | Laborda |
| Oriol | Roca |
| Jose Manuel | Allegue |
| Agueda | Ojados |
| M Carmen | Hornos |
| Mariana | Portilla |
| Federico | Gordo |
| Marcela | Homez |
| Alberto | Belenguer Muncharaz |
| Manuel | Castillo Quintero |
| Maria | Morales |
| Demetrio | Carriedo |
| Covadonga | Rodriguez |
| Silvia | Avila Fuentes |
| Natalia | Resano Sarmiento |
| Pablo | Garcia Olivares |
| Alexis Jaspe | Codecido |
| Raúl | de Pablo |
| Luis Alberto | Jaramillo |
| Ignacio | Saez |
| Susana | Temprano |
| Isidro | Prieto |
| Emilio | Maseda |
| Patricia | Salgado |
| Cesar | Perez Calvo |
| Anxela | Vidal |
| Enrique | Cereijo |
| Enrique | Platas |
| Juan Luis | Galeas-Lopez |
| Manuel | Herrera-Gutierrez |
| Manuel | Perez |
| Eugenio Luis | Palazon Sanchez |
| Maria Teresa | Millan |
| Mireia | Ferreruela |
| Catalina | Forteza |
| Gemma | Rialp |
| Javier | Izura |
| Juna | Tirapu |
| Conchita | Martínez-Fidalgo |
| Elisabet | Garcia |
| Imma | Vallverdu |
| Candelaria | de Haro |
| Felix | Martin |
| Meisy | Perez Cheng |
| Aitor | Olmos |
| Roser | Tomas |
| Diego | De Mendoza |
| Arantxa | Mas |
| Raquel | Montiel |
| Dácil | Parrilla |
| Alejandro | Gonzalez-Castro |
| Maria | Mora Aznar |
| Daniel | Moreno Torres |
| Neus | Guasch |
| Mònica | Magret Iglesias |
| Jesus Emilio | Barrueco-Francioni |
| Angela | Algaba |
| Carlos | Munoz de Cabo |
| Ferran | Roche-Campo |
| Gerardo | Aguilar |
| Carlos | Ferrando |
| Maria Lorena | Fernandez-Rodriguez |
| Estefania | Prol-Silva |
| David | Perez-Torres |
| Jesus | Sanchez-Ballesteros |
| Borja | Fernandez |
| Ana | Villagra |
| Antonio Luis | Ruiz-Aguilar |
| Marta | Asín-Corrochano |
| Begoña | Zalba-Etayo |
| Preveen | Banwarie |
| Bernardo | Panka |
| Narain | Boedjawan |
| Yvette | Chou-Lie |
| Dieneke | Kienhorst |
| Navin | Ramdhani |
| Dick | Nahar |
| Alisha | Van Axel |
| Björn | Ahlström |
| Anna | Mattsson |
| Martin | Spångfors |
| Johanna | Henriksson |
| Dan | Lind |
| Harald | Zetterquist |
| Helena | Mansson |
| Line | Samuelsson |
| Gaetano | Perchiazzi |
| Magnus | von Seth |
| Elena | Nikolic |
| Johan | Berkius |
| Philippe | Eckert |
| Lise | Piquilloud |
| Napplika | Kongpolprom |
| Nuttapol | Rittayamai |
| Krittika | Teerapuncharoen |
| Yuda | Sutherasan |
| Pongdhep | Theerawit |
| Tananchai | Petnak |
| Viratch | Tangsujaritvijit |
| Detajin | Junhasavasdikul |
| Cherdkiat | Karnjanarachata |
| Sunthiti | Morakul |
| Poungrat | Thungtitigul |
| Konlawij | Trongtrakul |
| Yutthana | Apichatbutr |
| Nadwipa | Yuangtrakul |
| Pattarin | Pirompanich |
| Narongkorn | Saiphoklang |
| Mohamed | Besbes |
| Amira | Jamoussi |
| Asma | Ben Souissi |
| Mhamed | Sami Mebazaa |
| Souheil | Elatrous |
| Nejla | Tilouch |
| Didem | Sozutek |
| Ozlem | Ozkan Kuscu |
| Ezgi | Özyılmaz |
| Avşar | Zerman |
| Sema | Sari |
| Sema | Turan |
| Semih | Aydemir |
| Hilal | Sazak |
| Gulbin | Aygencel |
| Melda | Turkoglu |
| Fatma | Yildirim |
| Melike | Cengiz |
| Ayca | Gumus |
| Feza | Bacakoglu |
| Pervin | Korkmaz Ekren |
| Nermin | Kelebek Girgin |
| Ayşe | Nur Soyturk |
| Türkay | Akbas |
| Serdar | Efe |
| Volkan | Inal |
| Gülseren | Elay |
| Huseyin | Arikan |
| Sait | Karakurt |
| Ismail | Cinel |
| Fethi | Gül |
| Suha | Bozbay |
| Oktay | Demirkiran |
| Yalim | Dikmen |
| Elif | Erdogan |
| Perihan | Ergin Ozcan |
| Figen | Esen |
| Nalan | Adiguzel |
| Ozlem | Yazicioglu Mocin |
| Mustafa | Akker |
| Zafer | Çukurova |
| Yasemin | Seker Tekdos |
| Cenk | Kirakli |
| Iskender | Kara |
| Faruk | Seçkin Yücesoy |
| Hilmi | Demirkiran |
| Arzu | Esen Tekeli |
| Pradeep | Shanmugasundaram |
| Simon | Chau |
| Sughrat | Siddiqui |
| Tim | Cook |
| Ian | Kerslake |
| Sarah | Snape |
| Ana | Vochin |
| Gavin | Perkins |
| Elliot | Yates |
| Owen | Boyd |
| Laura | Ortiz-Ruiz De Gordoa |
| Caroline | Kane |
| Matt | Thomas |
| Jeremy | Bewley |
| Lisa | Grimmer |
| Paul | Smith |
| Kaushik | Bhowmick |
| Sally | Humphreys |
| Tim | Smith |
| Antoinette | Wilson |
| Sarah | Beavis |
| Nick | Spittle |
| Phil | Chilton |
| Clare | Hammel |
| Sundar | Raj Ashok |
| Arif | Moghal |
| David | Wrathall |
| Christopher | Wright |
| David | Slessor |
| Dagmar | Holmquist |
| Rajnish | Saha |
| Lorraine | Stephenson |
| Tamaas | Leiner |
| Andrew | Hermon |
| Ceri | Lynch |
| Simon | Whiteley |
| Elizabeth | Wilby |
| Ingeborg | Welters |
| Karen | Williams |
| Rohit | Saha |
| Grisma | Patel |
| Elisa | Kam |
| Amy | Collins |
| Ahmad | Zaki |
| Reza | Khorasanee |
| Elliot | Bertram-Ralph |
| Daniel | Horner |
| Jayaprakash | Patil |
| Christos | Chaintoutis |
| Keith | Hugill |
| Isabel | Gonzalez |
| Jane | Adderley |
| Alex | Martin |
| Richard | Pugh |
| Venkat | Sundaram |
| Anil | Hormis |
| Mark | Smith |
| Govindan | Balaraj |
| Riccardo | Scano |
| Sunil | Jamadarkhana |
| Rakesh | Bhandary |
| Michele | Clark |
| Patricia | Doble |
| Richard | Innes |
| Thomas | Clark |
| Daniel | Paul |
| Karen | Burt |
| Mike | Spivey |
| Alastair | Rose |
| Samantha | Hagan |
| John | Trinder |
| Agnieszka | Kubisz-Pudelko |
| Jarone | Lee |
| Gabriel | Rodriguez |
| Elias | Baedorf Kassis |
| Valerie | Banner-Goodspeed |
| Renaud | Gueret |
| Aiman | Tulaimat |
| Dina | Gomaa |
| Betty | Tsuei |
| Abhijit | Duggal |
| Ashish K. | Khanna |
| Joshua A. | Englert |
| Michael | Wert |
| Christian | Tomaszewski |
| Gabriel | Wardi |
| Jeffrey | Barry |
| Christine M. | Bojanowski |
| Nancy | Glober |
| Christopher | Tainter |
| Elizabeth | Stevenson |
| Rahul | Nanchal |
| Jonathon | Truwit |
| Colin | Grissom |
| Michael | Lanspa |
| Andrew | McKown |
| Todd | Rice |
| Shigeki | Saito |
| Akram | Khan |
| Stephanie | Nonas |
| Philippe | Bauer |
| Nathan | Smischney |
| Richard | Oeckler |
| Ashish | Rai |
| Kevin | Eng |
| Sanjeev | Tyagi |
| David | Dries |
| Elizabeth | Ramey |
| Angela | Rogers |
| Jack | Short |
| Gaston | Aguirre |
| Alberto | Deicas |
| Rodrigo | Beltramelli |
| Arturo | Briva |
