# Supplementary material for: Impact of frailty and older age on weaning from invasive ventilation: a secondary analysis of the WEAN SAFE study
Source: Ann Intensive Care. 2025 Jan 20;15:13. doi: 10.1186/s13613-025-01435-1 (PMC11743409; doi:10.1186/s13613-025-01435-1)
Supplement: Supplementary file 4 — Supplementary Material 4 [file 13613_2025_1435_MOESM4_ESM.docx]

# IV- Appendix 1: List of participating ICUs

| 1. Unidad de Cuidados Intensivos, Sanatorio La Trinidad Mitre, Buenos Aires, Argentina |
| --- |
| 1. Unidad de Terapia Intensiva, Hospital D.F Santojanni, Buenos Aires, Argentina |
| 1. Unidad de Cuidados Intensivos, Sanatorio Anchorena, Buenos Aires, Argentina |
| 1. Unidad e Terapia Intensiva, Instituto De Investigaciones Medicas Alfredo Lanari, Buenos Aires, Argentina" |
| 1. Intensive Care Service, Otamendi Health Center, Buenos Aires, Argentina |
| 1. Intensive Care Unit, CEMIC, Buenos Aires, Argentina |
| 1. Intensive Care Unit, Bazterrica, Buenos Aires, Argentina |
| 1. Unidad de Terapia Intensiva de Adultos, Hospital Misericordia, Cordoba, Argentina |
| 1. Terapia Intensiva, Higa San Martin La Plata, La Plata, Argentina |
| 1. Terapia Intensiva, Instituto Medico Platense , La Plata, Argentina |
| 1. Terapia Intensiva Hospital Lagomaggiore, Hospital Luis Carlos Lagomaggiore, Mendoza, Argentina |
| 1. Terapia Intensiva Adultos, Hospital Nacional Profesor Alejandro Posadas, Moron, Argentina |
| 1. Unidad de Terapia Intensiva, Clinica San Agustín, Neuquén, Argentina |
| 1. Unidad de Terapia Intensiva, Hospital Provincial del Centenario, Rosario, Argentina |
| 1. Terapia Intensiva, Jose Maria Cullen, Santa Fe, Argentina |
| 1. Unidad de Cuidados Intensivos, Sanatorio Guemes, Buenos Aires, Argentina |
| 1. Unidad de Cuidados Intensivos, Clínica Santa Isabel, Buenos Aires, Argentina |
| 1. Terapia Intensiva de Quemados, Hospital Luis Carlos Lagomaggiore, Mendoza, Argentina |
| 1. Intensive Care, Hospital Central, Mendoza, Argentina |
| 1. UTI, Francisco Lopez Lima Hospital, General Roca, Argentina |
| 1. UTI, Clinica y maternidad suizo argentina , Buenos Aires, Argentina |
| 1. Unidad de Terapia Intensiva, Sanatorio de Los Arcos, Buenos Aires, Argentina |
| 1. Intensive and Critical Care Unit, Flinders Medical Centre, Adelaide, Australia |
| 1. Critical Care Unit, Ballarat Health Services, Ballarat, Australia |
| 1. ICU, Canberra Hospital, Canberra, Australia |
| 1. Concord Hospital Intensive Care Unit, Concord Hospital, Concord, NSW, Australia |
| 1. Midland Intensive Care Unit, St John of God Midland Public and Private Hospitals, Perth, Australia |
| 1. Intensive Care Unit, Armadale Health Service, Perth, Australia |
| 1. ICU, Epworth Richmond, Richmond, Australia |
| 1. Intensive Care Unit, St John of God Subiaco, Perth, Australia |
| 1. Intensive Care Unit, John Hunter Hospital, Newcastle , Australia |
| 1. Intensive Care Unit, SJG Murdoch Hospital, WA Murdoch Perth, Australia |
| 1. Intensive Care, Gold Coast University Hospital, Southport, QLD, Australia |
| 1. Maroondah Intensive Care Unit, Eastern Health, Maroondah Campus, East Ringwood, Australia |
| 1. Intensive Care Unit, Cairns and Hinterland Hospital, Cairns City, Australia |
| 1. Medical Intensive Care Unit, UZLeuven, Leuven, Belgium |
| 1. ICU, AZ Turnhout, Turnhout, Belgium |
| 1. ICU, CHU UcL Namur site Godinne (Godinne University Hospital), Yvoir, Belgium |
| 1. Intensieve zorgen , Imelda Ziekenhuis, Bonheiden, Belgium |
| 1. Unidad de Terapia Intensiva, Hospital Universitario Japonés, Santa Cruz, Bolivia |
| 1. Department for cardioanesthesia , Clinic for cardiosurgery, University Clinical Center Kosevo, Sarajevo, Bosnia and Herzegovina |
| 1. Centro de Terapia Intensiva da Santa Casa de Campo Grande, Associação Beneficiente de Campo Grande MS, Campo Grande - Mato Grosso do Sul - MS, Brazil |
| 1. UTI, HPS 28 de agosto, Manaus, Brazil |
| 1. UTI do PSM, Hospital das Clínicas da FMUSP, Sao Paulo, Brazil |
| 1. ICU Hospital BP Mirante, BP Mirante, Sao Paulo, Brazil |
| 1. Critical Care Medicine, Hospital Israelita Albert Einstein, Sao Paulo, Brazil |
| 1. UTI Geral, Vitoria Apart Hospital, Vitoria, Brazil |
| 1. Unidade de terapia intensiva geral, Hospital \|Unimed Vitória, Vitoria, Brazil |
| 1. Unidade de Terapia Intensiva, Hospital Cristo Redentor , Porto Alegre, Brazil |
| 1. Medical Surgical intensive care, St Michael's hospital, Toronto, Canada |
| 1. ICU, The Ottawa Hospital, Ottawa, Canada |
| 1. Critical Care Unit, North York General Hospital, Toronto, Canada |
| 1. Medical Surgical Intensive Care Unit, London Health Sciences Centre - University Hospital, London, Canada |
| 1. MSNICU, Toronto Western Hospital, Division of University Health Network, Toronto, Canada |
| 1. The Allan T. Lambert Trauma and Neurosurgery Intensive Care Unit (TNICU), St. Michael's Hospital, Toronto, Canada |
| 1. Unidad de Cuidados Intensivos, Hospital Clinico Universidad de Chile, Santiago, Chile |
| 1. Unidad de Cuidados Intensivos Adulto, Hospital Felix Bulnes Cerda, Santiago, Chile |
| 1. Unidad de Cuidados Intensivos Generales, Hospital Naval Almirante Nef, Viña del Mar, Chile |
| 1. Unidad de Paciente Crítico , Hospital Clínico Pontificia Universidad Católica de Chile, Santiago, Chile |
| 1. Departamento de Paciente Critico, Clinica Alemana de Santiago, Santiago, Chile |
| 1. Department of Critical Care Medicine, BinZhu, ChangZhou, China |
| 1. ICU, the FourthPeople`s Hospital Chang'zhou, Changzhou, China |
| 1. Department of Intensive Care Medicine, 1st affiliated Hospital of Dalian Medical university , Dalian, China |
| 1. Intensive Care Unit, Fujian Provincial Hospital, Fuzhou, China |
| 1. Intensive Care Unit, Guangdong General Hospital, Guangzhou, China |
| 1. Department of Surgical Intensive Care Unit, The First Affiliated Hospital, Sun Yat-sen University, Guangzhou, China |
| 1. Critical Care Medicine, Affiliated Hospital of Guiyang Medical University, Guiyang, China |
| 1. Jinxiang People's Hospital, Jin Xiang People's Hospital, Jining City, China |
| 1. Emergency Department and EICU/MICU, First Affiliated Hospital of Kunming Medical University, Kunming, China |
| 1. Cardiovascular Surgery Intensive Care Unit, Kunming Medical University Affiliated Yan’an Hospital, Kunming, China |
| 1. Emergency Intensive Care Unit, The First Affiliated Hospital of Henan University of Science & Technology, Luoyang, China |
| 1. Intensive Care Unit, Nanjing Drum Tower Hospital, Nanjing, China |
| 1. Intensive Care Unit, Zhongda Hospital, Nanjing, China |
| 1. Intensive Care Unit, Nanjing Jiangbei People's Hospital, Nanjing, China |
| 1. Sicu, First Affiliated Hospital of Guangxi Medical University, Nanning, China |
| 1. Intensive Care Unit, Affiliated Hospital of Nantong University, Nantong, China |
| 1. The Department of Critical Care Unit, Qilu Hospital of Shandong University of Qingdao, Qingdao, China |
| 1. Department of Critical Care Medicine,Ruijin Hospital, Shanghai Jiaotong University School of Medicine, Shanghai, China, Ruijin Hospital, Shanghai Jiaotong University School of Medicine, Shanghai, China, Shanghai, China |
| 1. ICU, Shanghai General Hospital, Shanghai, China |
| 1. Intensive Care Unit, Sheng Jing Hospital of China Medical University, Shenyang, China |
| 1. RICU of the Second Department of Respiration Medicine, The Second Hospital of Hebei Medical University, Shijiazhuang, China |
| 1. Intensive Care Unit, Changshu No.1 People’ s Hospital, Soochow, China |
| 1. ICU, Union Hospital, Tong Ji Medical College, Huazhong University of Science and Technology, Wuhan, Hubei, China |
| 1. Department of Critical Care Medicine, First Affiliated Hospital of Wannan Medical College, Yijishan Hospital, Wuhu, China |
| 1. ICU, WuXi People's Hospital, WuXi, China |
| 1. Departement of Crit Care Unit, The First Affiliated Hospital of Xiamen University, Xiamen, China |
| 1. Critical Care Medicine, The First Affiliated Hospital of Zhengzhou University, Zhengzhou, China |
| 1. Unidad de Cuidados Intensivos , Fundación Valle del Lilí, Cali, Colombia |
| 1. Intensive Care Unit, Hospital Vicente Corral Moscoso, Cuenca, Ecuador |
| 1. UCI, Clinica la Merced, Quito, Ecuador |
| 1. Réanimation Polyvalente, Centre Hospitalier Pierre Oudot, Bourgoin-Jallieu, France |
| 1. Réanimation Médicale, CHU Cavale Blanche, Brest , France |
| 1. Réanimation Polyvalente, Hopital d'Instruction des Armées Clermont Tonnerre, Brest , France |
| 1. Service de Reanimation Médicale, Centre Hospitalo-Universitaire de Caen, Caen, France |
| 1. Réanimation Polyvalente, Centre Hospitalier Public du Cotentin, Cherbourg en Cotentin, France |
| 1. Médecine Intensive Réanimation, Hôpital Louis Mourier, Assistance Publique - Hôpitaux de Paris Colombes, France |
| 1. Réanimation Adulte, Centre Hospitalier Intercommunal de Créteil, Créteil, France |
| 1. Médecine Intensive Réanimation, Centre Hospitalier de Dieppe, Dieppe, France |
| 1. Réanimation, Centre Hospitalier Universitaire Grenoble-Alpes, Grenoble , France |
| 1. Réanimation Médico-chirurgicale et Unité de Surveillance Continue, Centre Hospitalier Le Mans, Le Mans, France |
| 1. Critical Care Center, CHU de Lille, Hôpital R. Salengro, Lille, France |
| 1. Réanimation Polyvalente, Centre Hospitalier des Deux Vallées - Site de Longjumeau, Longjumeau, France |
| 1. Réanimation Medicale, Hôpital de la Croix Rousse, Lyon, France |
| 1. Réanimation - Médecine Intensive, Groupe Hospitalier Sud Ile de France - Site de Melun, Melun, France |
| 1. Réanimation médico-chirurgicale, CHU de Nice L'Archet 2, Nice, France |
| 1. Medical ICU, Medical ICU Archet 1 university Hospital, Nice, France |
| 1. Medical ICU, Saint Louis APHP, Paris, France |
| 1. Réanimation médico-chirurgicale, Groupe Hospitalier Paris Saint-Joseph, Paris, France |
| 1. Médecine Intensive Réanimation, Hôpital Cochin, Paris, France |
| 1. Médecine intensive réanimation, Hôpital Tenon, Paris, France |
| 1. Reanimation médicale, Hopital Europeen Georges Pompidou, Paris, France |
| 1. Servive de réanimation polyvalente et unité de soins-continus, centre hospitalier de cornouaille, Quimper, France |
| 1. Service de Réanimation , Centre Hospitalier, Roanne, France |
| 1. Réanimation Chirurgicale, CHU de Rouen, Rouen, France |
| 1. Medical intensive care unit, CHU de Rouen, Rouen, France |
| 1. Service de Médecine Intensive - Réanimation, Hôpital Delafontaine, Saint Denis, France |
| 1. Service de réanimation polyvalente, CHU Felix Guyon, Saint-Denis de la Réunion, France |
| 1. Réanimation médicale, Hopital de Hautepierre, Strasbourg, France |
| 1. Service de réanimation, Sainte Musse Hospital, Toulon, France |
| 1. Réanimation polyvalente, Hôpital Nord Franche Compte, Trévenans, France |
| 1. Service de Médecine Intensive – Réanimation (Département "R3S"), AP-HP. Sorbonne Université, Hôpital Pitié Salpêtrière, Paris, France |
| 1. Médecine Intensive Réanimation Médicale, CHU de Poitiers, Poitiers, France |
| 1. Reanimation polyvalente, Ghef site de jossigny, Jossigny, France |
| 1. Dept. of Internal Medicine V,, University Hospital of Saarland, Homburg, Germany |
| 1. Intensive Care Unit, Hippocration General Hospital of Athens, Athens, Greece |
| 1. ICU, General Hospital Katerini, Katerini, Greece |
| 1. ICU , IPPOKRATION General Hospital, Thessaloniki, Greece |
| 1. Intensive Care Unit, Asklepieion Voulas General Hospital, Voula Atikki, Greece |
| 1. B Intensive Care Unit Clinic, Attikon University Hospital, Athens, Greece |
| 1. Intensive Care Unit, Dr. Kenessey Albert Hospital, Balassagyarmat, Hungary |
| 1. ICU, Uzsoki Hospital, Budapest, Hungary |
| 1. Department of Anesthesiology and Intensive Therapy, University of Szeged, Szeged, Hungary |
| 1. ICU, St. George Hospital, Fejér County, Székesfehérvár, Hungary |
| 1. Anaesthesiologie, Universitaetsmedizin Goettingen, Goettingen, Germany |
| 1. EICU, CIMS Hospital, Ahmedabad , India |
| 1. Department of Neuroanaesthesia and Neurocritical Care, National Institute of Mental Health and NeuroSciences, Bangalore, India |
| 1. Critical Care Unit, Apollo Hospitals Bhubaneswar, Bhubaneswar, India |
| 1. ICU, KOVAI Medical Center And Hospital, Coimbatore, India |
| 1. Medical ICU, PSRI - Pushpawati Singhania Research Institute, Delhi, India |
| 1. Critical Care Unit, Virinchi Hospital, Hyderabad, India |
| 1. Critical Care Medicine , Sanjay Gandhi Postgraduate Institute of Medical Sciences (SGPGIMS), Lucknow, India |
| 1. Critical Care , Sir H N Reliance Foundation Hospital, Mumbai, India |
| 1. ICU, Criticare Hospital & Research Institute, Nagpur, India |
| 1. Intensive care unit, Metro heart institute with multispecialty, Faridabad, India |
| 1. Masih Daneshvari Critical Care unit, Masih Daneshvari (NRITLD), Tehran, Iran |
| 1. Intensive Care Unit, St Vincents University Hospital, Dublin, Ireland |
| 1. Intensive Care Unit, Galway University Hospital, Galway, Ireland |
| 1. Intensive Care Unit, Midland Regional Hospital, Mullingar, Ireland |
| 1. Intensive Care Unit, St James Hospital , Dublin, Ireland |
| 1. Intensive Care and Anaesthesia, University Hospital Limerick, Limerick, Ireland |
| 1. Anestesia e Rianimazione 3a - Terapia Intensiva Adulti, ASST Papa Giovanni XXIII - Bergamo, Bergamo, Italy |
| 1. U.O. Anestesiologia e Terapia Intensiva Polivalente e dei Trapianti, AOU di Bologna. Policlinico Sant'Orsola Malpighi, Bologna, Italy |
| 1. Seconda Rianimazione, Spedali Civili, Brescia , Italy |
| 1. Anestesia e Rianimazione, ASST-LARIANA presidio di Cantù, Cantù, Italy |
| 1. UOC Anestesia E Rianimazione\Antonella Caruso\, ARNAS Garibaldi Catania, Catania, Italy" |
| 1. Rianimazione e Terapia Intensiva, Mater Domini, Catanzaro, Italy |
| 1. Terapia Intensiva, Ospedale Uboldo - Cernusco sul Naviglio, Cernusco sul Naviglio, Italy |
| 1. Intensive Care Unit, ASST Franciacorta, Chiari (Brescia), Italy |
| 1. Anesthesia and General ICU, SS. Annunziata, Chieti, Italy |
| 1. Rianimazione E Terapia Intensiva, Bassini ASST Nord Milano, Cinisello Balsamo-Milano, Italy |
| 1. Intensive Care Unit, Arcispedale Sant'Anna -Ferrara, Ferrara, Italy |
| 1. Terapia Intensiva Oncologica, AOU Careggi, Florence, Italy |
| 1. Intensive Care , Azienda OORR-University of Foggia, Foggia, Italy |
| 1. Rianimazione e Terapia Intensiva, IRCCS San Martino IST Genova, Genova, Italy |
| 1. Rianimazione Generale, A.Manzoni, Lecco, Italy |
| 1. U.O. Rianimazione, Ospedale Civile di Legnano, Legnano, Italy |
| 1. Reparto di Terapia Intensiva, ASST Santi Paolo e Carlo - Ospedale San Paolo - Polo Universitario, Milan, Italy |
| 1. UO Rianimazione Generale, Azienda Ospedaliera Fatebenefratelli Sacco - Ospedale Sacco - Polo Universitario, Milan, Italy |
| 1. Neurorianimazione, ASST Grande Ospedale Metropolitano Niguarda, Milan, Italy |
| 1. Terapia Intensiva Generale 1, ASST Grande Ospedale Metropolitano Niguarda, Milan, Italy |
| 1. U.O. Neurorianimazione - Terapia Intensiva, Ospedale Civile Sant'Agostino Estense, Modena, Italy |
| 1. Terapia Intensiva Generale, ASST Monza, Monza, Italy |
| 1. Rianimazione, Ospedale Maggiore della Carità, Novara, Italy |
| 1. SCDU Anestesia e Rianimazione, Azienda Ospedaliero-Universitaria S. Luigi Gonzaga, Orbassano (TO), Italy |
| 1. Terapia Intensiva, Policlinico San Marco Zingonia, Osio Sotto (Bergamo), Italy |
| 1. ISTAR2, Azienda Ospedaliera di Padova, Padova, Italy |
| 1. UOS Terapia Intensiva, Ospedale Sant'Antonio, Padova, Italy |
| 1. UTIP, Policlinico Paolo Giaccone, Palermo, Italy |
| 1. Terapia Intensiva Polivalente con Trauma Center, ARNAS Ospedale Civico Di Cristina Benfratelli, Palermo, Italy |
| 1. Primo Servizio Anestesia e Rianimazione, AOU Ospedale Maggiore, Parma, Italy |
| 1. Rianimazione 1, Fondazione IRCCS Policlinico San Matteo, Pavia, Italy |
| 1. Intensive care unit, Ospedale Civile dello Spirito Santo, Pescara, Italy |
| 1. Rianimazione, Ospedale Santo Stefano Prato, Prato, Italy |
| 1. Anestesia E Rianimazione, S.Maria Delle Croci, Ravenna, Italy |
| 1. Intensive care unit, Infermi, Rimini, Italy |
| 1. Rianimazione, Policlinico Umberto I - Sapienza Università di Roma, Rome, Italy |
| 1. Intensive Care Unit, Policlinico Universitario Agostino Gemelli, Rome, Italy |
| 1. Centro Rianimazione, San Giovanni Addolorata, Rome, Italy |
| 1. Terapia Intensiva Generale, Istituto Clinico Humanitas, Rozzano (Milano), Italy |
| 1. UOC Anestesia e Rianimazione, Azienda Ospedaliera Universitaria San Giovanni di Dio e Ruggi D'aragona, Salerno, Italy |
| 1. Rianimazione 2, Asst Lariana Ospedale S.Anna di Como, San Fermo della Battaglia, Italy |
| 1. Unità di Terapia Intensiva, AOU Sassari - Cliniche Universitarie, Sassari, Italy |
| 1. Terapia Intensiva, ASST Nord MIlano Ospedale Città di Sesto San Giovanni, Sesto San Giovanni, Milano, Italy |
| 1. Rianimazione e Medicina critica, AOU Senese, Siena , Italy |
| 1. Department of Anaesthesia and Intensive Care , Città della Salute e della Scienza di Torino, Turin, Italy |
| 1. Rianimazione, San Giovanni Bosco, Turin, Italy |
| 1. Clinica di Anestesia e Rianimazione, ASUIUD \S. Maria della Misericordia\", Udine, Italy" |
| 1. Anestesia e Rianimazione N°1, Azienda Sanitaria Universitaria Integrata di Udine , Udine, Italy |
| 1. Anestesia e Rianimazione A, AOUI Verona, Verona, Italy |
| 1. Intensive Care Unit \E. Vecla\", Fondazione IRCCS Ca' Granda Ospedale Maggiore Policlinico, Milan, Italy" |
| 1. S.C. Anestesia e Rianimazione 2, Azienda Ospedaliera di Perugia, Perugia, Italy |
| 1. Post-Surgical Intensive Care, Fondazione IRCCS Policlinico San Matteo, Pavia, Italy |
| 1. UOC Terapia Intensiva e Anestesia, Grande Ospedale Metropolitano \Binchi-Melacrino-Morelli\", Reggio Calabria, Italy" |
| 1. Rianimazione, ASST Monza - Ospedale di Desio, Desio MB, Italy |
| 1. Rianimazione, ASST Bergamo Ovest Treviglio, Treviglio, Italy |
| 1. Anestesia Rianimazione 3, ASST Grande Ospedale Metropolitano Niguarda, Milan, Italy |
| 1. Intensive Care Unit, San Bortolo Hospital, Vicenza, Italy |
| 1. Terapia Intensiva 2, ASUIUD \Santa Maria della Misericordia\", Udine, Italy" |
| 1. ICU , Tokyo Women’s Medical University Yachiyo Medical Center, Chiba, Japan |
| 1. Advanced emergency and critical care center, Shinshu University Hospital, Matsumoto, Japan |
| 1. Emermgency Intensive Care Unit, Okayama University Hospital, Okayama, Japan |
| 1. ICU, Jichi Medical University Saitama Medical Center, Saitama, Japan |
| 1. Department of Critical Care Medicine, Sakai City Medical Center, Sakai City, Japan |
| 1. ICU, Tohoku University Hospital, Sendai, Japan |
| 1. Division of Intensive Care, Department of Anesthesiology and Intensive Care Medicine, Jichi Medical University School of Medicine, Shimotsuke, Tochigi, Japan |
| 1. ICU, Tokyo Bay Urayasu Ichikawa Medical Center, Urayasu, Japan |
| 1. Department of Emergency and Critical Care Medicine, Institute of Biomedical & Health Sciences, Hiroshima University Advanced Emergency and Critical Care Center, Hiroshima University Hospital , Hiroshima, Japan |
| 1. SICU, Kurume University Hospital, Kurume, Japan |
| 1. Department of Anesthesiology and Intensive Care, Hamamatsu University School of Medicine, Hamamatsu, Japan |
| 1. Intensive Care Unit, Kumamoto University Hospital, Kumamoto, Japan |
| 1. Intensive Care Unit, Jikei University hospital, Tokyo, Japan |
| 1. Emergensy and Critical care unit, Tokyo Medical Center, Tokyo, Japan |
| 1. Intensive Care Unit, Tokushima University Hospital, Tokushima, Japan |
| 1. Emergency Intensive Care Unit, Kurashiki Central Hospital, Okayama, Japan |
| 1. Department of Intensive and Intensive Care Medicine, JA Hiroshima General Hospital, Hatsukaichi city, Japan |
| 1. Department of Anesthesiology, Kurashiki Central Hospital, Kurashiki, Okayama, Japan |
| 1. Intensive Care Unit, Yokohama City Minato Red Cross Hospital, Yokohama, Japan |
| 1. ICU, Musashino Red Cross Hospital, Tokyo, Japan |
| 1. Intensive Care Unit, Okinawa Chubu Hospital, Uruma City, Okinawa, Japan |
| 1. MICI, Tripoli Medical Center, Tripoli, Libya |
| 1. Areas Criticas, Hospital General de Ecatepec \Las Americas\", Ecatepec, Mexico" |
| 1. Unidad de Cuidados Intensivos Adultos, Hospital Civil Guadalajara Juan I Menchaca, Guadalajara, Mexico |
| 1. Department of Critical Care Medicine, Fundación Clínica Médica Sur, Mexico City, Mexico |
| 1. Hospital General de Zona 48 San Pedro Xalpa, Hospital General de Zona 48, Mexico City, Mexico |
| 1. Unidad de Cuidados Intensivos, Hospital Regional 1 de Octubre, Mexico City, Mexico |
| 1. General Critical care unit, IMSS Hospital de Especialidades Antonio Fraga Mouret, Mexico City, Mexico |
| 1. Unidad de Cuidados Intensivos Respiratorios, Instituto Nacional de Enfermedades Respiratorias, Mexico City, Mexico |
| 1. Unidad de Cuidados Intensivos, IMSS Unidad Médica de Alta Especialidad No. 21, Monterrey, Mexico |
| 1. UCIA , HGR Clinica 72, Tlalnepantla , Mexico |
| 1. Service de Réanimation Médicale, Ibn Sina Hospital, Rabat, Morocco |
| 1. Neurosurgical ICU, Specialities Hospital, Rabat, Morocco |
| 1. Medical ICU, Mohammed VIth University Hospital, Marrakech, Morocco |
| 1. Intensive Care Unit, Grande International Hospital, Kathmandu, Nepal |
| 1. Intensive Care Unit, T U Teaching Hospital, Kathmandu, Nepal |
| 1. ICV, VU University Medical Centre Amsterdam, Amsterdam, Netherlands |
| 1. ICU, Academic Medical Center, Amsterdam, Netherlands |
| 1. Intensive Care Unit, Maastricht University Medical Centre+, Maastricht, Netherlands |
| 1. Intensive Care and Medium Care, Radboudumc, Nijmegen, Netherlands |
| 1. Intensive Care, Medisch Spectrum Twente, Enschede, Netherlands |
| 1. Intensive Care , Maasstad Ziekenhuis, Rotterdam, Netherlands |
| 1. Intensive Care Unit, Jeroen Bosch Ziekenhuis, 's-Hertogenbosch, Netherlands |
| 1. Cardiothoracic and Vascular ICU, Cardiothoracic and Vascular ICU, Auckland City Hospital, Auckland, New Zealand |
| 1. Intensive Care, Hutt Hospital, Lower Hutt, New Zealand |
| 1. Intensive Care Unit, Rotorua Hospital, Rotorua, New Zealand |
| 1. Intensive Care Unit, Wellington Hospital, Wellington, New Zealand |
| 1. Intensive Care Unit, Department for surgical services, Haukeland University Hospital, Bergen, Norway |
| 1. Intensive care unit (AKUM), Nordland Hospital (Nordlandssykehuset Boodø), Bodø, Norway |
| 1. Intensive care unit, Sykehuset Innlandet Hamar, Hamar, Norway |
| 1. Intensive care unit, Sorlandet Hospital, Kristiansand, Norway |
| 1. Intensive Care Unit, Akershus University Hospital, Lørenskog, Norway |
| 1. ICU, Sykehus Molde, Molde, Norway |
| 1. General Intensive Care Unit, Rikshospitalet Medical Centre, Oslo University Hospital, Oslo, Norway |
| 1. Intensive Care Unit 2M, Stavanger University Hospital, Stavanger, Norway |
| 1. Dept. Anaesthesia and Intensive care, Aalesund hospital, Aalesund, Norway |
| 1. Medical Intensive Care Unit/ Surgical Intensive Care Unit, Pakistan Institute Of Medical Sciences, Islamabad, Pakistan |
| 1. Surgical ICU, Pakistan Institute Of Medical Sciences, Islamabad, Pakistan |
| 1. Unidad de Cuidados Intensivos I, National Hospital Daniel Alcides Carrion, Bellavista District, Province Callao, Peru |
| 1. Critical Care Unit, Hospital Nacional Alberto Sabogal Sologuren, Callao, Peru |
| 1. Area Critica, Hospital Regional Lambayeque, Chiclayo, Peru |
| 1. Unidad de Cuidados Intensivos, Hospital Regional Ica, Ica, Peru |
| 1. Unidad de Cuidados Intensivos I, Hospital Edgardo Rebagliati Martins, Lima, Peru |
| 1. Unidad de Cuidados Intensivos 2, Hospital Edgardo Rebagliati Martins, Lima, Peru |
| 1. Unidad de Cuidados Intensivos 3, Hospital Edgardo Rebagliati Martins, Lima, Peru |
| 1. Departamento de Medicina Critica - UCI, Instituto Nacional Enfermedades Neoplasicas, Lima, Peru |
| 1. Unidad de Cuidados Intensivos, Clinica ONCOSALUD-AUNA, Lima, Peru |
| 1. Servicio de cuidados criticos, Hospital Nacional Dos de Mayo, Lima, Peru |
| 1. Unidad de Cuidados Intensivos, Hospital de Emergencias Grau, Lima, Peru |
| 1. Intensive Care Unit, Centro Medico Naval\CMST\, Lima, Peru |
| 1. Servicio de Cuidados Críticos, Hospital Nacional Dos de Mayo, Lima, Peru |
| 1. Intensive care unit, Hospital de apoyo Santa Rosa, Lima, Peru |
| 1. Unidad de Cuidados Intensivos, Hospital Regional de Huacho, Huacho, Peru |
| 1. Cuidados Intensivos, Hospital de emergencias Jose Casimiro ULLOA, Miraflores, Peru |
| 1. Department of Anaesthesiology and Intensive Care, Uniwersyteckie Centrum Kliniczne SUM, Katowice, Poland |
| 1. Skawinska ICU, University Hospital in Krakow, Krakow, Poland |
| 1. 1st Anaesthesiology and Intensive Care Unit, University Hospital Krakow, Krakow, Poland |
| 1. Department of Anesthesiology and Intensive Therapy, Wroclaw University Hospital, Wroclaw, Poland |
| 1. Department of intensive care , University Hospital Krakow, Krakow, Poland |
| 1. Unidade de cuidados Intensivos, Centro Hospitalar Cova da Beira, Covilhã, Portugal |
| 1. Unidade de Urgência Médica, Centro Hospitalar Lisboa Central - Hospital de São José, Lisboa, Portugal |
| 1. Unidade de Cuidados Intensivos Polivalente, Hospital Sao Francisco Xavier, Lisboa, Portugal |
| 1. Intensive Care Department 2, Centro Hospitalar Universitário do Algarve, Portimão, Portugal |
| 1. Unidade de Cuidados Intensivos Polivalente, Hospital Santo Antonio, Porto, Portugal |
| 1. Serviço de Medicina Intensiva Polivalente, Centro Hospitalar Entre Douro & Vouga, Santa Maria da Feira, Portugal |
| 1. Serviço de Medicina Intensiva, Hospital Garcia de Orta, Almada, Portugal |
| 1. UCI Polivalente Geral, Hospital S.João, Porto, Portugal |
| 1. Unidade de Cuidados Intensivos I - SCI 1, Centro Hospitalar do Porto, Porto, Portugal |
| 1. Serviço Cuidados Intensivos - Unidade Marc Velge, Centro Hospitalar de Setúbal - Hospital de São Bernardo, Setúbal, Portugal |
| 1. Anaesthesiology and Intensive Care, Krasnoyarsk Clinical Regional Hospital, Krasnoyarsk, Russian Federation |
| 1. Department of Anesthesiology and Intensive care, Regional Clinical Hospital ?2, Vladivostok, Russian Federation |
| 1. ICU no. 2, Moscow State Clinical Hospital named after V.V.Vinogradova , Moscow, Russian Federation |
| 1. Icu, Saudi German Hospital , Jeddah, Saudi Arabia |
| 1. Intensive Care Service Dept. , Prince Sultan Military Medical City , Riyadh, Saudi Arabia |
| 1. Adult ICU (ICU-2), King Abdulaziz Medical City, Riyadh, Saudi Arabia |
| 1. ICU department, Care National Hospital, Riyadh , Saudi Arabia |
| 1. Intensive care units, AFHSR Armed Forced Hospital Southern Region, Khamis Mushyte, Saudi Arabia |
| 1. Trauma ICU, Clinical Center of Serbia Emergency center , Belgrade, Serbia |
| 1. SOK E, Clinical center of Serbia -Emergency center , Belgrade, Serbia |
| 1. Center for Internal Intensive Medicine, University Medical Center Ljubljana, Ljubljana, Slovenia |
| 1. Medical Intensive Care Unit, Seoul National University Bundang Hospital, Seongnam, South Korea |
| 1. ICU, Ewha Womans University Mokdong Hospital , Seoul, South Korea |
| 1. ICU, Seoul National University Hospital , Seoul, South Korea |
| 1. ICU, Samsung Medical Center, Seoul, South Korea |
| 1. Medical intensive care unit, Inje University Sanggye Paik Hospital, Seoul, South Korea |
| 1. ICU, Seoul St.Mary's Hospital, Seoul, South Korea |
| 1. MICU, Soon Chun Hyang University Hospital Seoul, Seoul, South Korea |
| 1. Department of Medical Intensive Care Unit, Severance Hospital, Yonsei University College of Medicine, Seoul, South Korea |
| 1. ICU, Uiheongbu St. Mary's Hospital, Uijeongbu-si, Gyeonggi-do, South Korea |
| 1. ICU, Wonju Severance Christian Hospital, Wonju, South Korea |
| 1. MICU, ASAN Medical Center, Seoul, South Korea |
| 1. SICU, Ewha Womans University Mokdong Hospital, Seoul, South Korea |
| 1. ICU, Hanyang University Guri Hospital, Guri-si, South Korea |
| 1. Intensive Care Unit, Hospital Punta de Europa, Algeciras, Spain |
| 1. Servicio de Medicina Intensiva, Hospital Universitari Germans Trias i Pujol, Badalona/Barcelona, Spain |
| 1. Unidad Cuidados Intensivos, CST Terrassa, Barcelona, Spain |
| 1. Intensive Care Unit, Hospital del Mar, Barcelona, Spain |
| 1. Unidad de Cuidados Intensivos , Hospital General Universitario de Ciudad Real, Ciudad Real, Spain |
| 1. Unidad de Cuidados Intensivos, Hospital Universitario del Henares, Coslada, Spain |
| 1. Servicio de Medicina Intensiva, Juan Ramón Jiménez , Huelva, Spain |
| 1. Critical Care Unit, H.G.U Gregorio Marañón, Madrid, Spain |
| 1. Department of Intensive Medicine, Hospital Universitario Ramon y Cajal, Madrid, Spain |
| 1. Unidad de Cuidados Intensiva.- Servicio de Medicina Intensiva, Hospital Universitario 12 de Octubre, Madrid, Spain |
| 1. UCI de Trauma y Emergencias, Hospital Universitario 12 de Octubre, Madrid, Spain |
| 1. ICU of Anesthesia, Hospital Universitario La Paz, Madrid, Spain |
| 1. ICU, Hospital Regional Universitario de Malaga, Malaga, Spain |
| 1. Unidad de Cuidados Intensivos, Hospital Rey Juan Carlos, Mostoles (Madrid), Spain |
| 1. Servicio de Medicina Intensiva (ICU), Hospital Universitario Reina Sofia de Murcia, Murcia, Spain |
| 1. Unidad de Cuidados Intensivos, Hospital Universitario Son Espases, Palma de Mallorca, Spain |
| 1. Intensive Care Department, Hospital Son LLàtzer, Palma de Mallorca, Spain |
| 1. UCI, Complejo Hospitalario de Navarra, Pamplona Navarra, Spain |
| 1. Unidad de Cuidados Intensivos, Hospital Universitario Infanta Cristina, Parla (Madrid), Spain |
| 1. Intensive Care Unit, Hospital Universitari Sant Joan, Reus, Spain |
| 1. UCI , Corporació Sanitària i Universitària Parc Taulí, Sabadell, Spain |
| 1. UCI, Hospital Clínico de Salmanca, Salamanca, Spain |
| 1. UCI, Hospital Universitario General de Catalunya, Sant Cugat del Valles, Spain |
| 1. Servei de Medicina Intensiva, Hospital Sant Joan Despí Moisès Broggi, Sant Joan Despí, Spain |
| 1. Critical Care Unit, Hospital Universitario Ntra Sra de Candelaria, Santa Cruz de Tenerife, Spain |
| 1. UCIG-A, Hospital Universitario Marqués de Valdecilla, Santander, Spain |
| 1. Intensive Care Unit, Hospital Joan XXIII, Tarragona, Spain |
| 1. Intensive Care Unit, Hospital Universitario de Torrejón, Torrejon de Ardoz, Spain |
| 1. Servei de medicina intensiva, Hospital Verge de la Cinta, Tortosa, Spain |
| 1. Unidad de Reanimación, Hospital Clínico Universitario of Valencia, Valencia, Spain |
| 1. TRAUMA-Quemados, Rio Hortega, Valladolid, Spain |
| 1. Polivalente, Rio Hortega, Valladolid, Spain |
| 1. Intensive Care Unit, Hospital Universitario de Alava, Vitoria-Gasteiz, Spain |
| 1. UCI Traumatología, Miguel Servet Universitary Hospital, Zaragoza, Spain |
| 1. Critical Care Unit, Universitary Clinic Lozano Blesa, Zaragoza, Spain |
| 1. Unidad de Cuidados Intensivos, Hospital de la Princesa, Madrid, Spain |
| 1. Adult Intensive care Unit, CHUV-University Hospital of Lausanne, Lausanne, Switzerland |
| 1. Unidad de Cuidados Intensivos, Hospital Universitario de Getafe, Getafe, Madrid, Spain |
| 1. UCI, Hospital Santa Bárbara de Soria, Soria, Spain |
| 1. Surgical Intensive Care Unit, Hospital del Mar - Parc de Salut , Barcelona , Spain |
| 1. Criticado café unit, Hospital Universitario Príncipe de Asturias, Alcalá de Henares, Spain |
| 1. Intensive Care Unit, Hospital General Universitari de Castelló, Castelló , Spain |
| 1. Servicio de Medicina Intensiva, Hospital de la Santa Creu i Sant Pau, Barcelona, Spain |
| 1. Unidad de Cuidados Intensivos del Hospital de Leon, Complejo asistencial Universitario de Leon, Leon, Spain |
| 1. Critical Care Department, Hospital Universitari Vall d'Hebron, Barcelona, Spain |
| 1. Intensive Care Unit, National Hospital for Paraplegics, Toledo, Spain |
| 1. Unidad de Cuidados Críticos, Hospital Universitario Fundación Alcorcón, Alcorcón, Spain |
| 1. Pulmonary and Critical Care Unit, King Chulalongkorn Memorial Hospital, Bangkok, Thailand |
| 1. Respiratory Intensive Care Unit, Faculty of Medicine Siriraj Hospital, Bangkok, Thailand |
| 1. 9IC Unit, Ramathibodi Hospital , Bangkok, Thailand |
| 1. 8IK Unit, Ramathibodi Hospital , Bangkok, Thailand |
| 1. 7NW Unit, Ramathibodi Hospital , Bangkok, Thailand |
| 1. 3IC Unit, Ramathibodi, Bangkok, Thailand |
| 1. EMICU, Vajira Hospital, Bangkok, Thailand |
| 1. ICU, Thammasat University Hospital, Pathumthani, Thailand |
| 1. Medical ICU, Vajira Hospital , Bangkok, Thailand |
| 1. Anesthesiology and ICU Department, Mongi Slim Hospital, La Marsa, La Marsa, Tunisia |
| 1. Service Réanimation Médicale, Taher Sfar Hospital Mahdia, Mahdia, Tunisia |
| 1. Internal Medicine Intensive Care Unit, Çukurova University Balcali Hospital , Adana, Turkey |
| 1. Respiratory ICU, Cukurova University Hospital , Adana, Turkey |
| 1. Medical Intensive Care Unit , Toros neighorhood, Adana , Turkey |
| 1. Intensive Care Unit, Türker High Aducation and Research Hospital, Ankara, Turkey |
| 1. Medical Intensive Care Unit 1 and 2, S.B.Ü Atatürk Chest Diseases and Thoracic Surgery Hospital SUAM, Ankara, Turkey |
| 1. Gazi University Hospital, Medical Intensive Care Unit, Gazi University Hospital, Ankara, Turkey |
| 1. General Surgical Intensive Care Unit, Ankara Diskapi Yildirim Beyazit Research and Education Hospital, Ankara, Turkey |
| 1. Anesthesiology Intensive Care Unit, Akdeniz University Hospital, Antalya, Turkey |
| 1. Chest Disease Department, Medicine School of Ege University, Bornova/Izmir, Turkey |
| 1. Medical Intensive Care, Düzce University Hospital, Düzce, Turkey |
| 1. Medical Intensive Care Unit, Marmara University Hospital, Istanbul, Turkey |
| 1. Sadi Sun Intensive Care Unit, Istanbul University Cerrahpasa Medical Faculty, Istanbul, Turkey |
| 1. Medical Intensive Care Unit, Sureyyapasa Chest Diseases and Thoracic Surgery Training Hospital, Istanbul, Turkey |
| 1. Anesthesiology and Reanimation, Marmara University Hospital, Istanbul, Turkey |
| 1. Adult Intensive Care Unit, Sisli Etfal Educational Research Hospital, Istanbul, Turkey |
| 1. Intensive Care Unit, Dr. Suat Seren Chest Diseases and Surgery Training Hospital, Izmir, Turkey |
| 1. General Intensive Care 1-2-3, Konya Numune Hastanesi, Konya, Turkey |
| 1. intensive care, Erciyes Unicersity Faculty of Medicine Department of Internal Critical cCre Unit, Kayseri, Turkey |
| 1. Anesthesiology Intensive Care, University of Istanbul - Istanbul Medical Faculty, Istanbul, Turkey |
| 1. Intensive Care Unit, Stoke Mandeville Hospital, Aylesbury, Buckinghamshire, UK - England |
| 1. Barnsley Hospital Intensive Care Unit., Barnsley Hospital NHS Foundation Trust, Barnsley, UK - England |
| 1. Intensive Care Unit, Royal United Hospitals Bath NHS Foundation Trust, Bath, UK - England |
| 1. Critical Care Complex, Bedford Hospital NHS trust, Bedford, UK - England |
| 1. Intensive Care Unit, Heart of England NHS Foundation Trust, Birmingham, UK - England |
| 1. Intensive Care Unit, Royal Sussex County Hospital, Brighton, UK - England |
| 1. General Intensive Care Unit A600, Southmead Hospital, Bristol, UK - England |
| 1. Intensive Care Unit, Bristol Royal Infirmary, Bristol, UK - England |
| 1. ITU, Burton Hospitals NHS FT, Burton on Trent, UK - England |
| 1. Anaesthetics and Critical Care Unit, West Suffolk Hospital NHS Foundation Trust, Bury St Edmunds, UK - England |
| 1. ITU Cumberland Infirmary, NCUHTrust , Carlisle, UK - England |
| 1. Chesterfield Royal Department of Critical Care, Chesterfield Royal nhs foundation trust, Chesterfield, UK - England |
| 1. Critical Care Unit, Leighton Hospital, Crewe, UK - England |
| 1. Intensive Care Unit, Croydon Health Services NHS Trust, Croydon , UK - England |
| 1. Critical Care Unit, The Princess Alexandra Hospital NHS Trust, Harlow, UK - England |
| 1. ICU, Harrogate District Hospital NHS Foundation Trust, Harrogate, UK - England |
| 1. Critical Care Centre, North West Anglia NHS Foundation Trust Hinchingbrooke Hospital , Huntingdon , UK - England |
| 1. Intensive Care Unit (J54), St James's University Hosptial , Leeds, UK - England |
| 1. Intensive Care Unit, Royal Liverpool University Hospital, Liverpool, UK - England |
| 1. Christine Brown Intensive Care Unit, King's College Hospital, London, UK - England |
| 1. ITU, Hillingdon Hospital, London, UK - England |
| 1. Intensive Care Unit, Salford Royal NHS Foundation Trust, Manchester, UK - England |
| 1. Department of Critical Care, Milton Keynes University Hospital, Milton Keynes, UK - England |
| 1. Critical Care Unit, The Rotherham NHS Foundation Trust, Rotherham, UK - England |
| 1. Intensive Care Unit, South Tyneside NHS Foundation Trust, South Shields, UK - England |
| 1. General Intensive Care Unit, Lister Hospital, Stevenage , UK - England |
| 1. Critical Care Unit, University Hospital North Tees, Stockton-on-Tees, UK - England |
| 1. Critical Care, Musgrove Park, Taunton, UK - England |
| 1. Intensive Care Unit, Torbay Hospital, Torquay, UK - England |
| 1. Critical Care, Royal Cornwall Hospital, Truro, UK - England |
| 1. Critical Care Unit, Pinderfields Hospital, Wakefield, UK - England |
| 1. Intesive Care , Ulster Hospital, Belfast, UK - Northern Ireland |
| 1. Intensive Care Unit, Dumfries and Galloway Royal Infirmary, Dumfries, UK - Scotland |
| 1. Intensive Care Unit, Queen Elizabeth University Hospital, Glasgow, Glasgow, UK - Scotland |
| 1. Intensive care unit, Royal Glamorgan Hospital, Llantrisant, UK - Wales |
| 1. Intensive Care Unit, Yeovil District Hospital, Yeovil, UK - England |
| 1. Intensive Care Unit, Glan Clwyd Hospital, Rhyl, UK - Wales |
| 1. Intensive Care Unit, Royal Surrey County Hospital, Guildford Surrey, UK - England |
| 1. Intensive Care Unit, North Manchester General Hospital, Manchester, UK - England |
| 1. ICU, Guy's and St Thomas' Hospitals , London, UK - England |
| 1. Departamento de Medicina Intensiva, CASMU, Montevideo, Uruguay |
| 1. Catedra Medicina Intensiva, Hospital de Clinicas, Montevideo, Uruguay |
| 1. Blake 12 ICU, Massachussetts General Hospital, Boston, USA |
| 1. SICU, Beth Isreal Deaconess Medical Center, Boston, USA |
| 1. Medical Intesive Care Unit, John H Stroger Hospital of Cook County, Chicago, USA |
| 1. ICU, University of Cincinnati Medical Center , Cincinnati, Ohio, USA |
| 1. Surgical ( SICU +CVICU ) and Medical ICU , Cleveland Clinic Foundation, Cleveland , USA |
| 1. Division of Pulmonary and Critical Care Medicine, The Ohio State University Wexner Medical , Columbus, USA |
| 1. El Centro Regional Medical Center ICU, El Centro Regional Medical Center, El Centro, California, USA |
| 1. Medical, Surgical, Cardiac, Neuro, University of California San Diego - La Jolla campus, La Jolla, USA |
| 1. Sulpizio Cardiovascular Center ICU, UCSD Sulpizio, La Jolla, USA |
| 1. MICU, North Shore Medical Centre, Salem, USA |
| 1. Shock Trauma ICU and Respiratory ICU, Intermountain Medical Center, Murray, USA |
| 1. Medical Intensive Care Unit, Tulane Medical Center, New Orleans, USA |
| 1. Medical ICU, Oregon Health & Science University, Portland, USA |
| 1. Mary Brigh 7 D/E, Mayo Clinic Rochester, Rochester, USA |
| 1. MICU, Mayo Clinic St Marys Campus, Rochester, USA |
| 1. 10-3 and 10-4, Mayo Clinic, Methodist Campus, Rochester, USA |
| 1. Medical Intensive care unit, NSMC Union Hospital, Lynn, USA |
| 1. 10ICU, University of California San Diego Medical Center - Hillcrest, San Diego, USA |
| 1. Surgical Intensive Care, Medical Intensive Care Unit, Regions Hospital, St Paul, USA |
| 1. Medical ICU, Vanderbilt University Medical Center, Nashville, USA |
| 1. E2 ICU, Stanford , Stanford, USA |
| 1. Medical Intensive Care Unit, Froedtert and the Medical College of Wisconsin, Milwaukee, USA |
| 1. ICU, s' Lands Hospitaal Paramaribo, Paramaribo, Suriname |
| 1. ICU Diakonessenhuis, Diakonessenhuis, Paramaribo, Suriname |
| 1. Intensive Care, Academisch Ziekenhuis Paramaribo, Paramaribo, Suriname |
| 1. Intensive Care, Sint Vincentius Hospital, Paramaribo, Suriname |
| 1. Critical Care Medicine department, Alexandria University Hospital, Alexandria, Egypt |
| 1. ICU, Alexandria Main University Hospital, Alexandria, Egypt |
| 1. Respiratory ICU, Zagazig University Hospitals Chest Department , Zagazig, Egypt |
| 1. Critical Care Medicine Unit, Menoufia University Hospitals, Shibin El-kom, Egypt |
| 1. Intensive care unit, Square Hospitals LTD, Dhaka , Bangladesh |
| 1. ICU, Kristianstad Hospital, Kristianstad, Sweden |
| 1. Centrala intensivvårdsavdelningen, Akademiska sjukhuset, Uppsala, Sweden |
| 1. Department of Anesthesilogy and Intesive Care, Sunderby Hospital, Luleå, Sweden |
| 1. IVA Västervikssjukhus, Västervikssjukhus, Västervik, Sweden |
| 1. Operationskliniken, Västmanlands Sjukhus Västerås, Västerås, Sweden |
| 1. IVA, Falun, Falu lasarett, Falun, Sweden |
| 1. Östersund Intensive Care, Östersunds Hospital, Östersund, Sweden |
| 1. IVA, Nykopings Lasarett, Nykoping, Sweden |
| 1. ICU, Hôpital Bernard Mevs, Port-Au-Prince, Haïti |
| 1. ICU - Cardiac Adult Surgery, Acibadem City Clinic Tokuda Hospial, Sofia, Bulgaria |

# V - Appendix 2: List of national National Societies/Networks endorsing the Study

European Society of Intensive Care Medicine, European Respiratory Society, ANZICS Clinical Trials Group, Indian Society of Critical Care Medicine, Irish Critical Care Trials Group; Société de Réanimation de Langue Française (SRLF); Réseau Européen de Recherche en Ventilation Artificielle (ReVA Network); Société Française d’Anesthésie et de Réanimation (SFAR); Society of Critical Care Medicine (Discovery Network).

# V - Appendix 3: List of WEAN SAFE investigators

**WEAN SAFE Steering Committee:** Tài Pham, Leo Heunks, Giacomo Bellani, Fabiana Madotto; Gaëtan Beduneau, Ewan C. Goligher, Giacomo Grasselli, Jordi Mancebo, Lise Piquilloud, Antonio Pesenti, Hannah Wunsch, Frank van Haren, Laurent Brochard, John G. Laffey.

**WEAN SAFE National Coordinators**: Argentina: Elisa Estenssoro; Australia/New Zealand: Frank Van Haren; Belgium: Greet Hermans; Brazil: Ary Serpa Neto; China: Haibo Qiu; Canada: Ewan Goligher; Chile: Guillermo Bugedo; Czechia: Vladimir Cerny; Egypt: Assem Abdel Razek; France: Gaëtan Beduneau, Sébastien Perbet; Germany: Onnen Moerer; Greece: Dimitrios Matamis; Haiti: Alfred Papali; Hungary: Zsolt Molnar; India: Pravin Amin; Iran: Sayed Mohammadreza Hashemian; Ireland: Kevin Clarkson; Italy: Giacomo Grasselli; Japan: Kiyoyasu Kurahashi; Nepal: Subhash P Acharya; Mexico: Asisclo Villagomez; Morocco: Amine Ali Zeggwagh; Netherlands: Leo M. Heunks; Norway: Jon Henrik Laake; Peru: Rollin Roldan; Poland: Konstanty Szuldrzynski; Portugal: Irene Aragao; Romania: Dana Tomescu; Russia: Alexey Gritsan; Saudi Arabia: Yaseen Arabi; Serbia: Bojan Jovanovic; South Korea: Young-Jae Cho; Spain: Óscar Peñuelas; Suriname: Bernardo Panka; Sweden: Johan Berkius; Switzerland: Lise Piquilloud; Thailand: Nuttapol Rittayamai; Tunisia: Fekri Abrough; Turkey: Ezgi Ozylmaz; United Kingdom: Luigi Camporota; United States: Philippe Bauer, Daniel Talmor, Jeremy Beitler.

**List of WEAN SAFE collaborators (by country)**

**ALBANIA**: University Hospital Shefqet Ndroqi (Tirana): Alma Cani;

**ARGENTINA:** Sanatorio La Trinidad Mitre (Buenos Aires): Sebastian Fredes, Santiago Ilutovich; Hospital D.F Santojanni (Buenos Aires): Marco Bezzi, Silvina Borello; Sanatorio Anchorena (Buenos Aires): Gustavo Plotnikow, Romina Pratto; Instituto de Investigaciones Medicas Alfredo Lanari (Buenos Aires): Nicolas Iezzi, Rodolfo Lopez; Otamendi Health Center (Buenos Aires): Mariano Andres Furche, Paolo Nahuel Rubatto Birri; CEMIC (Buenos Aires): Pablo Lovazzano, Mariano Setten; Clinica Bazterrica (Buenos Aires): Matilde Grando, Vanesa Alejandra Pavlotsky; Sanatorio Guemes (Buenos Aires): Daniela Benvenuti; Clínica Santa Isabel (Buenos Aires): Eliana Markman, Graciela Paz; Clinica y Maternidad Suizo Argentina (Buenos Aires): Aldana Ruiz Robledo; Sanatorio de Los Arcos (Buenos Aires): Raúl Alejandro Gomez, María Florencia Valenti; Hospital Misericordia (Cordoba): Anatilde Diaz, Analía Garcia; Francisco Lopez Lima Hospital (General Roca): Rosana Hernandez, Maria Cristina Orlandi; HIGA San Martin La Plata (La Plata): Juan Conde, Rosa Reina; Instituto Médico Platense (La Plata): Gustavogcha Chaparro; Hospital Luis Carlos Lagomaggiore Terapia Intensiva (Mendoza): Gonzalo Pagella, Graciela Zakalik; Hospital Luis Carlos Lagomaggiore Terapia Intensiva de Quemados (Mondoza): Carlos Pellegrini, Ariel Chena; Hospital Central (Mendoza): Maria Fernanda Farina, Claudia Elizabeth Lopez; Hospital Nacional Profesor Alejandro Posadas (Moron): Fernando Rios, Judith Sagardia; Clinica San Agustín (Neuquén): María Elena Romano, Cristina Villegas Succar; Hospital Provincial del Centenario (Rosario): Lisandro Roberto Bettini, Luis Pablo Cardonnet; Sanatorio Las Lomas (San Isidro, Buenos Aires): Fernando Rios, Alejandro Risso Vazquez; Ramón Carrillo (Santiago del Estero): Robert Giannoni; José Maria Cullen (Santa Fe): Gabriela Bai, Santiago Izza; Hospital Municipal Dr. B. A. Houssay (Vicente López): Miguel Escobar, Patricia Sanchez;

**AUSTRALIA:** Flinders Medical Centre (Adelaide): Andrew Bersten, Shailesh Bihari; Ballarat Health Services (Ballarat): Dianne Hill, Angus Richardson; Eastern Health (Box Hill): Graeme Duke, Stephanie Hunter; Canberra Hospital (Canberra): Bernie Bissett, Frank Van Haren; Concord Hospital (Concord): Mark Kol, Asim Shah; Eastern Health, Maroondah Campus (East Ringwood): Peter Oziemski, Deborah Welsh; Cairns and Hinterland Hospital (Cairns): Vijayanand Palaniswamy; John Hunter Hospital (Newcastle): Kathryn Kerr, Ameet Parekh; St John of God Murdoch Hospital (Perth): Ege Eroglu, Adrian Regli; St John of God Midland Public and Private Hospitals (Perth): Edward Fysh; Armadale Health Service (Perth): David Blythe, Muraleekrishnan Muthukrishnan; St John of God Subiaco Hospital (Perth): Janet Ferrier, Edward Litton; Epworth Richmond (Richmond): Gabrielle Hanlon, Jonathan Barrett; Gold Coast University Hospital (Southport): James McCullough, Mandy Tallott;

**BANGLADESH**: Square Hospitals Ltd (Dhaka): Shihan Mahmud Redwanul Huq, Raihan Rabbani;

**BELGIUM:** Imelda Ziekenhuis (Bonheiden): Eric Frans; UZ Leuven (Leuven): Helga Ceunen, Greet Hermans; AZ Turnhout (Turnhout): Filiep Soetens, Marc Vanhoof; CHU UCL Namur, Mont-Godinne University Hospital (Yvoir): Pierre Bulpa; Isabelle Michaux;

**BOLIVIA:** Hospital Universitario Japonés (Santa Cruz): Mónica Crespo Ramirez, Orlando Gordillo Romero;

**BOSNIA AND HERZEGOVINA:** Clinic for Cardiosurgery, University Clinical Center Kosevo (Sarajevo): Sanja Granov Grabovica, Slavenka Straus;

**BRAZIL:** Hospital Lifecenter (Belo Horizonte): Bruno Vilela Costa Pinto; Associação beneficiente de Campo Grande MS (Campo Grande - Mato Grosso do Sul): Maria Augusta Rahe Pereira, Edys Tamasato; HPS 28 de Agosto (Manaus): Wilson Oliveira Filho, Jocyelle Vieira; Hospital Cristo Redentor (Porto Alegre): Fernanda Kutchak, Marcelo Rieder; Hospital das Clinicas de Pernambuco (Recife): Fabianne Dantas; UDI Hospital (São Luis): Louise Gondim; Hospital das Clínicas da FMUSP (São Paulo): Luciano Azevedo, Leandro Taniguchi; BP Mirante (São Paulo): Fernando da Silva Ramos; Hospital Israelita Albert Einstein (São Paulo) : Ary Serpa Neto, Karina Timenetsky; Vitoria Apart Hospital (Vitoria): Stephanie Piras, Claudio Piras; Hospital Unimed Vitória (Vitoria): Eliana Caser, Betania Silva Sales;

**BULGARIA**: Acibadem CITY Clinic Tokuda Hospial (Sofia): Margarita Borislavova;

**CANADA:** London Health Sciences Centre University Hospital (London): Karen J. Bosma, Michael Mikhaeil; The Ottawa Hospital (Ottawa): Andrew Seely; St Michael's Hospital (Toronto): Laurent Brochard, Thai Pham; St. Michael's Hospital Allan T Lambert TNICU (Toronto): Ricard Mellado Artigas, Thomas Piraino; North York General Hospital (Toronto): Phil Shin; Toronto Western Hospital (Toronto): Sharique Ansari, Victoria McCredie;

**CHILE**: Hospital Clinico Universidad de Chile (Santiago): Daniel Arellano, Rodrigo Cornejo; Hospital Felix Bulnes Cerda (Santiago): Matias Jesús Flamm Zamorano, Manuel Gonzalez; Hospital Clínico Pontificia Universidad Católica de Chile (Santiago): Guillermo Bugedo, David Carpio; Clinica Alemana de Santiago (Santiago): Jerónimo Graf Santos, Rodrigo Pérez-Araos; Hospital Naval Almirante Nef (Viña del Mar): Eduardo Labarca; Facultad de Medicina, Escuela de Medicina, Universidad Andrés Bello (Viña del Mar): Felipe Martinez;

**CHINA**: Changzhou First People’s Hospital (ChangZhou): Bin Zhu; Changzhou Fourth People`s Hospital (Changzhou): Guojun Pan, Chen Shuhua; First Affiliated Hospital of Dalian Medical University (Dalian): Jiuzhi Zhang; Fujian Provincial Hospital (Fuzhou): Kai Chen, Rongguo Yu; Guangdong General Hospital (Guangzhou): Tiehe Qin, Shouhong Wang; The First Affiliated Hospital Sun Yat-sen University (Guangzhou): Xiang-Dong Guan, Jian-Feng Wu, Bi-Lin Wei; Affiliated Hospital of Guiyang Medical University (Guiyang): Feng Feng; Jin Xiang People's Hospital (Jining): Meihong Hou, Hongwen Zhang; First Affiliated Hospital of Kunming Medical University (Kunming): Chuanyun Qian, Wei Zhang; Kunming Medical University Affiliated Yan’an Hospital (Kunming): Jia Zheng, Zheng-Jiang Xing; The First Affiliated Hospital of Henan University of Science & Technology (Luoyang): Dahuan Li; Guoxiu Zhang; Nanjing Drum Tower Hospital (Nanjing): Qing Gu, Ning Liu; Zhongda Hospital (Nanjing): Ling Liu, Haibo Qiu; Nanjing Jiangbei People's Hospital (Nanjing): Chengqing Mei, Zhenglong Ye; First Affiliated Hospital of Guangxi Medical University (Nanning): Liangyan Jiang, Zhanhong Tang; Affiliated Hospital of Nantong University (Nantong): Chenliang Sun, Hongsheng Zhao; Qilu Hospital of Shandong University (Qingdao): Wu Dawei, Guo Xi; Ruijin Hospital, Shanghai Jiaotong University School of Medicine (Shanghai): Jialin Liu, Hongping Qu; Shanghai General Hospital (Shanghai): Wang Ruilan, Xie Yun; Sheng Jing Hospital of China Medical University (Shenyang): Bin Zang; Peking University Shenzhen Hospital (Shenzhen): Hua Luo, Weixin Zhang; The Second Hospital of Hebei Medical University (Shijiazhuang): Wensen Pan, Boyun Yuan; Changshu First People’s Hospital (Soochow): Yufeng Feng, Min Lu; Suining Central Hospital (Suining): Xia Hongtao, Gong Yu; Union Hospital, Tong Ji Medical College, Huazhong University of Science and Technology (Wuhan): You Shang, Xiaobo Yang; First Affiliated Hospital of Wannan Medical College, Yijishan Hospital (Wuhu): Yupeng Qi, Tao Yu; WuXi People's Hospital (WuXi): Hongyang Xu, Jie Yan; The First Affiliated Hospital of Xiamen University (Xiamen): Chen Jing, Zhang Minwei; The First Affiliated Hospital of Zhengzhou University (Zhengzhou): Hongbin Li, Rongqing Sun;

**COLOMBIA**: Fundación Valle del Lilí (Cali): Mónica Vargas-Ordoñez;

**COSTA RICA**: Hospital San Juan de Dios (San José): Juan Ignacio Silesky Jimenez;

**ECUADOR**: Hospital Vicente Corral Moscoso (Cuenca): Hernan Aguirre-Bermeo; Hospital de Especialidades Eugenio Espejo (Quito): Diego Rolando Morocho Tutillo, Andrea Gabriela Peña Padilla; Clinica la Merced (Quito): Diana Alvarez, María Fernanda Garcia;

**EGYPT**: Alexandria University Hospital (Alexandria): Mohamed Elsaadany, Hany Elsayed; Alexandria Main University Hospital (Alexandria): Samar Elsayed; Menoufia University Hospitals (Shibin El-Kom): Abdelrhman Aboshady, Nagwa Doha; Zagazig University Hospitals Chest Department (Zagazig): Eman Shebl;

**FRANCE**: Centre Hospitalier Pierre Oudot (Bourgoin-Jallieu): Philippe Crova, Thuy Nga Phan; CHU Cavale Blanche (Brest): Simon Bocher, Gwenael Prat; Hôpital d'Instruction des Armées Clermont Tonnerre (Brest): Marc Danguy des Déserts, Françoise Labat; Centre Hospitalo-Universitaire de Caen (Caen): Cédric Daubin, Aurélie Joret; Centre Hospitalier Public du Cotentin (Cherbourg): Bertrand Sauneuf, Xavier Souloy; Hôpital Louis Mourier (Colombes): Malo Emery, Damien Roux; Centre Hospitalier Intercommunal de Créteil (Créteil): Frédérique Schortgen; Centre Hospitalier de Dieppe (Dieppe): Pierre-Louis Declercq, Stéphanie Gelinotte; Centre Hospitalier Universitaire Grenoble-Alpes (Grenoble): Louis-Marie Galerneau, Nicolas Terzi; GHEF site de Marne-la-Vallée (Jossigny): Frank Chemouni, Jonathan, Zarka, Centre Hospitalier Le Mans (le Mans): Nicolas Chudeau; CHU de Lille, Hôpital R. Salengro (Lille): Saad Nseir, Anahita Rouze; Centre Hospitalier des Deux Vallées - Site de Longjumeau (Longjumeau): Matthieu Le Meur, Martial Thyrault; Hôpital Croix Rousse (Lyon): Claude Guérin; Groupe Hospitalier Sud Ile de France - Site de Melun (Melun): Jonathan Chelly, Sébastien Jochmans; CHU de Nice L'Archet 2 (Nice): Pierre-Eric Danin, Louis Humbert; CHU de Nice l’Archet 1 (Nice): Jean Dellamonica, Mathieu Buscot; Hôpital Saint-Louis (Paris): Virginie Lemiale, Etienne Ghrenassia; Groupe Hospitalier Paris Saint-Joseph (Paris): Cédric Bruel, François Philippart; Hôpital Cochin (Paris): Jean-François Llitjos, Nathalie Marin; Hôpital Tenon (Paris): Muriel Fartoukh, Guillaume Voiriot; Hopital Européen Georges Pompidou (Paris): Emmanuel Guerot; Pitié-Salpêtrière (Paris): Maxens Decavèle, Martin Dres; CHU de Poitiers (Poitiers): Faustine Reynaud, Arnaud W. Thille; Centre Hospitalier de Cornouaille (Quimper): Alexandre Tonnelier; Centre Hospitalier de Roanne (Roanne): Pascal Beuret; Hôpital Jacques Puel (Rodez): Sébastien Ena; CHU de Rouen - SICU (Rouen): Philippe Gouin, Pierre-Gildas Guitard; CHU de Rouen – MICU : Elisabeth Surlemont, Laurie Lagache; Hôpital Delafontaine (Saint-Denis): Gabriel Preda, Daniel Silva; CHU Felix Guyon (Saint-Denis): Laurence Dangers, Benjamin Delmas; Hôpital de Hautepierre, Hôpitaux Universitaires de Strasbourg (Strasbourg): Jean-Etienne Herbrecht, Francis Schneider; Hôpital Sainte-Musse (Toulon): Jean-Michel Arnal, Aude Garnero; Hôpital Nord Franche-Comté (Trévenans): Julio Badie, Loïc Barrot;

**GERMANY**: Universitaetsmedizin Goettingen (Goettingen): Onnen Moerer; University Hospital of Saarland (Homburg): Philipp M. Lepper, Frederik Seiler;

**GREECE**: Hippocration General Hospital of Athens (Athens): Metaxia Papanikolaou, Theonymfi Papavasilopoulou; Attikon University Hospital (Athens): Olympia Apostolopoulou, Chrysi Diakaki; General Hospital of Katerini (Katerini): Panagiotis Ioannides, Marina Oikonomou; Hippocration General Hospital (Thessaloniki): Eleni Mass, Eleni Mouloudi; Asklepieion Voulas General Hospital (Voula Athens): Aikaterini Dimoula, Sofia Nikolakopoulou;

**HAITI**: Hospital Bernard Mevs (Port-au-Prince): Stacy House, Monaly Rivette ;

**HUNGARY**: Dr. Kenessey Albert Hospital (Balassagyarmat): Csaba Kopitko, László Medve; Uzsoki Hospital (Budapest): Zoltan Kulcsar, Zsuzsanna Szabo; University Hospital of Szeged (Szeged): Zsolt Molnar, Nándo Öveges; St. George Hospital (Székesfehérvár): Agnes Sarkany;

**INDIA**: CIMS Hospital (Ahmedabad): Shuchi Kaushik, Bhagyesh Shah; National Institute of Mental Health and NeuroSciences (Bangalore): Radhakrishnan Muthuchellappan, Ramesh Vj; Apollo Hospitals (Bhubaneswar): Saroj Pattnaik, Banambar Ray; SOA University IMS & Sum Hospital (Bhubaneswar): Sanghamitra Mishra, Basanta Kumar Pati; Kovai Medical Centre and Hospital (Coimbatore): Sivakumar Nandakumar, Lakshmikantcharan Saravanabavan; PSRI - Pushpawati Singhania Research Institute (Dehli): Lakshay Bhakhtiani, Simant Jha; Metro Heart Institute With Multispecialty (Faridabad): Vijay Kumar Agrawal, Prakash Khairnar; Virinchi Hospital (Hyderabad): Srinivas Samavedam; Sanjay Gandhi Postgraduate Institute of Medical Science (Lucknow): Arvind Baronia, Mohan Gurjar; Sir H N Reliance Foundation Hospital (Mumbai): Mayur Patel, Darshana Rathod; Criticare Hospital & Research Institute (Nagpur): Harshal Bawangade, Deepak Jeswani; Yashoda Hospitals (Secunderabad): Harish Mallapura Maheswarappa;

**IRAN**: Masih Daneshvari (NRITLD) (Tehran): Seyed Mohammadreza Hashemian, Hamidreza Jamaati;

**IRELAND**: St Vincents University Hospital (Dublin): Laura Flood, Alistair Nichol; St James Hospital (Dublin): Ignacio Martin-Loeches, Lindi Snyman; Galway University Hospital (Galway): Kevin Clarkson, Rooney Grainne; University Hospital Limerick (Limerick): Catherine Motherway, Don Walsh; Midland Regional Hospital (Mullingar): Mohammad Faheem;

**ITALY**: Azienda Ospedaliero-Universitaria Policlinico (Bari): Salavtore Grasso, Tania Stripoli; ASST Papa Giovanni XXIII (Bergamo): Alessandra Nasi, Ivano Riva; AOU di Bologna. Policlinico Sant'Orsola Malpighi (Bologna): Elisabetta Pierucci, Rocco D’Andrea; Spedali Civili (Brescia): Elisabetta Pecci; ASST-Lariana Presidio di Cantù (Cantù): Rinaldo Grasso, Gianmario Monza; ARNAS Garibaldi Catania (Catania): Jessica Maugeri, Agrippino Bellissima; Mater Domini (Catanzaro): Eugenio Garofalo, Paolo Navalesi; Ospedale Uboldo (Cernusco sul Naviglio): Massimo Zambon; ASST Franciacorta (Chiari): Paolo Gnesin, Manuel Todeschini; SS. Annunziata (Chieti): Salvatore Maurizio Maggiore; Bassini ASST Nord Milano (Cinisello Balsamo-Milano): Stefano Muttini; ASST Monza - Ospedale di Desio (Desio): Eduardo Beck, Alberto Facchini; Ospedale "Sacra Famiglia" - PLV Fatebenefratelli (Erba): Luca Guatteri; Arcispedale Sant'Anna (Ferrara): Savino Spadaro, Carlo Alberto Volta; AOU Careggi (Firenze): Cosimo Chelazzi; Azienda OORR-University of Foggia (Foggia): Gilda Cinnella, Lucia Mirabella; IRCCS San Martino IST Genova (Genova): Alexandre Molin, Fabio Tarantino; Ospedale A.Manzoni (Lecco): Andrea Coppadoro, Ettore Vascotto; Ospedale civile di Legnano (Legnano): Francesca Orsenigo, Virginia Porta; ASST Santi Paolo e Carlo, Ospedale San Paolo - Polo universitario (Milan): Davide Chiumello, Giovanni Mistraletti; Azienda Ospedaliera Fatebenefratelli Sacco - Ospedale Sacco - Polo Universitario (Milan): Antonio Castelli, Riccardo Colombo; ASST Grande Ospedale Metropolitano Niguarda Neurorianimazione (Milan): Francesco Curto; ASST Grande Ospedale Metropolitano Niguarda Terapia Intensiva Generale (Milan): Roberto Fumagalli, Riccardo Pinciroli; Fondazione IRCCS Ca' Granda Ospedale Maggiore Policlinico (Milan): Giacomo Grasselli, Monica Savioli; ASST Grande Ospedale Metropolitano Niguarda Rianimazione 3 (Milan): Maurizio Bottiroli; Ospedale Civile Sant'Agostino Estense (Modena): Maurizio Pavesi; ASST Monza (Monza): Giacomo Bellani; Ospedale Maggiore della Carità (Novara): Carlo Oliveri, Rosanna Vaschetto; Azienda Ospedaliero-Universitaria S. Luigi Gonzaga (Orbassano): Pietro Caironi; Policlinico San Marco Zingonia (Osio Sotto): Bruno Ballico, Giovanni Vitale; Azienda Ospedaliera di Padova (Padova): Paolo Persona, Tommaso Tonetti; Ospedale Sant'Antonio (Padova): Sabrina Boraso, Laura Pasin; Policlinico Paolo Giaccone (Palermo): Andrea Cortegiani, Mariachiara Ippolito; ARNAS Ospedale Civico di Cristina Benfratelli (Palermo): Andrea Neville Cracchiolo, Maria Teresa Strano; AOU Ospedale Maggiore (Parma): Edoardo Picetti, Emanuele Sani; Fondazione IRCCS Policlinico San Matteo Rianimazione 1 (Pavia): Mirko Belliato, Giorgio Antonio Iotti; Fondazione IRCCS Policlinico San Matteo Post-Surgical Intensive Care (Pavia): Anna Aliberti, Francesco Mojoli; Azienda Ospedaliera di Perugia (Perugia): Angelo Giacomucci; Ospedale Civile dello Spirito Santo (Pescara): Antonella Frattari; Azienda Ospedaliero Universitaria Pisana (Pisa): Pietro Bertini, Fabio Guarracino; Ospedale Santo Stefano Prato (Prato): Iacopo Cappellini, Guglielmo Consales; Santa.Maria delle Croci (Ravenna): Maurizio Fusari, Gianluca Zani; Grande Ospedale Metropolitano Binchi-Melacrino-Morelli (Reggio Calabria): Andrea Bruni, Sebastiano Macheda; Infermi (Rimini): Laura Bernabe; Policlinico Umberto I - Sapienza Università di Roma (Roma): Edoardo Piervincenzi, Marco Ranieri; Policlinico Universitario Agostino Gemelli (Roma): Gennaro De Pascale, Luca Montini; San Giovanni Addolorata (Roma): Roberta Caccese; IRCCS Humanitas Research Hospital (Rozzano): Yari Gollo, Valeria Lascari; Azienda Ospedaliera Universitaria San Giovanni di Dio e Ruggi D'Aragona (Salerna): Antonella Fortunato, Salvatore Palmese; Asst Lariana Ospedale S.Anna di Como (San Fermo della Battaglia): Marco Spagnoli, Simone Maria Zerbi; AOU Sassari - Cliniche Universitarie (Sassari): Leda Floris, Pierpaolo Terragni; ASST Nord Milano Ospedale Città di Sesto San Giovanni (Sesto San Giovanni): Stefano Clementi; AOU Senese (Siena): Rosella Barbieri, Lucia Cubattoli; Città della Salute e della Scienza di Torino (Torino): Vito Fanelli, Gabriele Sales; San Giovanni Bosco (Torino): Stefania Sovatzis; ASST Bergamo Ovest Treviglio (Treviglio): Massimo Borelli, Federica Vagginelli; ASUIUD "S. Maria della Misericordia" Terapia Intensiva 1 (Udine): Paolo Chiarandini, Manuela Lugano; ASUIUD "Santa Maria della Misericordia" Terapia Intensiva 2 (Udine): Stefania Buttera; Azienda Sanitaria Universitaria Integrata di Udine (Udine): Andrea Gigante, Francesca Lucchese; AOUI Verona (Verona): Domenico Gelormini; San Bortolo Hospital (Vicenza): Elisa Boni, Silvia De Rosa;

**JAPAN**: Tokyo Women’s Medical University Yachiyo Medical Center (Chiba): Moe Oguchi, Tomohito Sadahiro; Hamamatsu University School of Medicine (Hamamatsu): Yukako Obata; JA Hiroshima General Hospital (Hatsukaichi): Sakuraya Masaaki, Akihiro Takaba; (Hiroshima University Advanced Emergency and Critical Care Center (Hiroshima): Shinichiro Ohshimo, Nobuaki Shime; Kumamoto University Hospital (Kumamoto): Hidenobu Kamohara; Kurashiki Central Hospital (Kurashiki, Okayama): Hiromasa Irie; Kurume University Hospital (Kurume): Koichi Arinaga, Shuhei Niiyama; Shinshu University Hospital (Matsumoto): Katsunori Mochizuki, Kenichi Nitta; Okayama University Hospital (Okayama): Tetsuya Yumoto; Kurashiki Central Hospital (Okayama): Akira Kuriyama, Misuzu Nakanishi; Jichi Medical University Saitama Medical Center (Saitama): Masamitsu Sanui; Sakai City Medical Center (Sakai City): Junji Kumasawa; Tohoku University Hospital (Sendai): Takuya Shiga, Norifumi Yoshida; Jichi Medical University School of Medicine (Shimotsuke, Tochigi): Shinshu Katayama; Tokushima University Hospital (Tokushima): Taiga Itagaki; International University of Health and Welfare Mita Hospital (Tokyo): Kiyoyasu Kurahashi, Kazuya Omura; Jikei University School of Medicine (Tokyo): Kengo Asano; Tokyo Medical Center (Tokyo): Kei Ota; Musashino Red Cross Hospital (Tokyo): Kotaro Yamamoto; Toyooka Hospital (Toyooka, Hyogo): Daisuke Taniguchi; Tokyo Bay Urayasu Ichikawa Medical Center (Urayasu): Jun Kataoka; Juntendo University Urayasu Hospital (Urayasu): Hiroki Iriyama, Toshikazu Abe; Okinawa Chubu Hospital (Uruma City, Okinawa): Izumi Nakayama; Yokohama City Minato Red Cross Hospital (Yokohama): Isao Nagata;

**LIBYA**: Tripoli University Hospital (Tripoli): Mohamed Benlamin, Abubaker S ELmaryul;

**MEXICO**: Hospital General de Ecatepec "Las Americas" (Ecatepec): Felipe de Jesus Montelongo; Hospital Civil Guadalajara Juan i Menchaca (Guadalajara): Victor Hugo Madrigal Robles, Daniel Rodriguez Gonzalez; Fundación Clínica Médica Sur (Mexico City): Silvio Antonio Namendys-Silva; Hospital General de Zona 48 (Mexico City): Claudia Lopez Nava, Nandyelly San Juan Roman; Hospital Regional 1 de Octubre (Mexico City): Maria del Carmen Marin, Asisclo Villagomez; IMSS Hospital de Especialidades Antonio Fraga Mouret (Mexico City): Nancy Canedo, Alejandro Esquivel; Instituto Nacional de Enfermedades Respiratorias (Mexico City): Carmen Hernandez, Gustavo Lugo Goytia; IMSS Unidad Médica de Alta Especialidad No. 21 (Monterrey): Antonio Landaverde Lopez, Miguel Ángel Sosa Medellin; HGR Clinica 72 (Tlalnepantla): Anaid Manzano;

**MOROCCO**: University Hassan II in Casablanca University Teaching Hospital Ibn Rushd (Casablanca): Abdellatif Benslama, Hanane Ezzouine; Mohammed VI University Hospital (Marrakech): Abdelhamid Hachimi; Mohammed I University in Oujda, Mohammed 6 Hospital-Oujda, ICU (Oujda): Brahim Housni; Ibn Sina Hospital Medical ICU Mohammed V University in Rabat (Rabat): Tarek Dendane, Amine Ali Zeggwagh; Specialities Hospital (Rabat): Doumiri Mouhssine, Maazouzi Wajdi;

**NEPAL**: Grande International Hospital (Kathmandu): Subhash Acharya, Anand Thakur; Teaching University Hospital (Kathmandu): Subhash Acharya, Prabha Gautam;

**NETHERLANDS**: VU University Medical Center Amsterdam (Amsterdam): Leo Heunks, Ingrid van den Hul; Academic Medical Center (Amsterdam): Luigi Pisani, Marcus J. Schultz; Medisch Spectrum Twente (Enschede): Martin Rinket, Jan Wytze Vermeijden; Maastricht University Medical Centre (Maastricht): Melanie Acampo-de Jong, Serge Heines; Radboudumc (Nijmegen): Tim Frenzel, Hans van der Hoeven; Maasstad Ziekenhuis (Rotterdam): Nardo Van Der Meer, Dolf Weller; Jeroen Bosch Ziekenhuis ('s-Hertogenbosch): Koen Simons;

**NEW ZEALAND**: Auckland City Hospital (Auckland): Rachael Parke, Shay McGuinness; Hutt Hospital (Lower Hutt): Carmel Chapman, Andrew Stapleton; Rotorua Hospital (Rotorua): Ulrike Buehner, Erin Williams; Wellington Hospital (Wellington): Nina Beehre;

**NORWAY**: Aalesund Hospital (Aalesund): Finn H. Andersen; Haukeland University Hospital (Bergen): Brit Ågot Sjøbø, Gabriele Leonie Schwarz; Nordland Hospital (Bodø): Knut Dybwik; Sykehuset Innlandet Hamar (Hamar): Bror Anders Johnstad, Terje Legernaes; Sorlandet Hospital (Kristiansand): Ole Georg Vinorum, Nils Christian Wenn-Velken; Akershus University Hospital (Lørenskog): Martin Fluckiger; Sykehus Molde (Molde): Lutz Fehrle; Oslo University Hospital, Rikshospitalet Medical Centre (Oslo): Tayyba Naz Aslam, Jon Henrik Laake; Stavanger University Hospital (Stavanger): Linda Rørtveit, Kristian Strand;

**PAKISTAN**: Pakistan Institute Of Medical Sciences MICU (Islamabad): Muneeb Ali, Taha Pasha; Pakistan Institute Of Medical Sciences SICU (Islamabad): Rakhshanda Jabeen, Kamal Nasir;

**PERU**: Hospital Eegional Honorio Delgado (Arequipa): Cecilia Eugenia Chavez; National Hospital Daniel Alcides Carrion (Bellavista): Patricia Gutierrez, Tapia Muñoz; Hospital Nacional Alberto Sabogal Sologuren (Callao): Jorge Cabrera, Willy Porras; Hospital Regional Lambayeque (Chiclayo): Luis Coaguila, Giovanna Soto; Hospital Regional de Huacho (Huacho): Rosita Gomero Paredes, Martin Santos; Hospital regional Ica (Ica): Jesus Milagrito Avalos Cabrera, Ivan Canchos Gutierrez; Hospital Edgardo Rebagliati Martins UCI 1 (Lima): Hector Higo Leon Yoshido, Ronald Zumaran; Hospital Edgardo Rebagliati Martins UCI 2 (Lima): Guillermo Malpartida, José Portugal; Hospital Edgardo Rebagliati Martins UCI 3 (Lima): Gabriel Omar Heredia Orbegoso, Xandra Yanina Rodriguez Tucto; Instituto Nacional Enfermedades Neoplasicas (Lima): Ronald Perez Maita, Rocio Quispe Soto; Clinica Oncosalud-Auna (Lima): Helbert Esquivel Gallegos, Rocio Qiospe Soto; Hospital Guillermo Almenara (Lima): José Cruz, Enrique Paz; Hospital Nacional Dos de Mayo UCI 1 (Lima): Willy Diaz, Oscar Gomez; Hospital Nacional Dos de Mayo UCI 2 (Lima): Rainier Ovalle Olmos, Rosario Quispe Sierra; Hospital de Emergencias Grau (Lima): Peter Malaga, Yazcitk Sandoval; Centro Medico Naval CMST (Lima): Manuel Alberto Laca Barrera, Fernando Pachas Alvarado; Hospital de Apoyo Santa Rosa (Lima): Teobaldo Quintana, Julio Yáñez; Hospital de Emergencias Jose Casimiro ULLOA (Miraflores): Luis Herrera, Olga Milagros, Mestanza Arica;

**POLAND**: Uniwersyteckie Centrum Kliniczne SUM (Katowice): Piotr Czempik; University Hospital in Krakow Skawinska ICU (Krakow): Milosz Jankowski, Konstanty Szuldrzynski; University Hospital in Krakow ICU & Anaesthesiology 1 (Krakow): Jaroslaw Garlicki; University Hospital Krakow Dept of Critical care (Krakow): Wojciech Serednicki, Jadwiga Wojtas; Wroclaw University Hospital (Wroclaw): Jakub Smiechowicz;

**PORTUGA**L: CHMT - Hospital Abrantes (Abrantes): Nuno Catorze, Tiago Pereira; Hospital Garcia de Orta (Almada): Rui Gomes, Vera Pereira; Centro Hospitalar Cova da Beira (Covilhã): Cristina Coxo; Centro Hospitalar Lisboa Central - Hospital de São José (Lisbon): Luis Bento, Sara Ventura; Centro Hospitalar Lisboa Ocidental (CHLO), Hospital Sao Francisco Xavier (Lisbon): Victor Mendes, Pedro Povoa; Centro Hospitalar Universitário do Algarve (Portimão): Maksym Dykyy, Juan Hidalgo; Hospital S. João (Porto): Maria Teresa Oliveira, Ana Vaz; Centro Hospitalar Universitário do Porto (Porto), Hospital Santo Antonio: Heloisa Castro, Maria João Ferreira da Silva; Centro Hospitalar Entre Douro e Vouga (Santa Maria da Feira): Tiago Leonor, Elsa Sousa; Centro Hospitalar de Setúbal - Hospital de São Bernardo (Setubal): João Carvalho, Guilherme Domingos; CHTMAD - Vila Real (Vila Real): Ana Raquel Lima, Igor Milet; Centro Hospitalar Tondela-Viseu (Viseu): Luis Patão, Carla Santos;

**RUSSIA**: Regional Clinical Hospital (Chita): Andrey Malyarchikov, Konstantin Shapovalov; Professor V.F. Voino-Yasenetsky Krasnoyarsk State Medical University, Krasnoyarsk Clinical Regional Hospital (Krasnoyarsk): Andrey Gazenkampf, Alexey Gritsan; Moscow State Clinical Hospital V.V.Vinogradova (Mosscow): Marina Petrova, Maria Vatsik; Regional Clinical Hospital №2 (Vladivostok): Pavel Dunts, Oleg Li;

**SAUDI ARABIA**: Saudi German Hospital (Jeddah): Maie Salem; Prince Sultan Military Medical City (Riyadh): Ghamdan Al Sadeh, Mohamed Mustafa; King Abdulaziz Medical City (Riyadh): Yaseen Arabi; Care National Hospital (Riyadh): Sultan Alamri, Mohamed Rabee; Prince Mohamed Bin Abdelaziz Hospital (Riyadh): Ahmed Rabie, Mostafa Rajab; AFHSR Armed Forced Hospital Southern Region (Khamis Mushyte): Mervat Mohamed Khalaf Ebraheim;

**SERBIA**: Clinical Center of Serbia Emergency Center Trauma ICU (Belgrade): Adi Hadzibegovic, Bojan Jovanovic; Clinical Center of Serbia Emergency Center SOK E (Belgrade): Branislava Stefanovic;

**SLOVENIA**: University Medical Center Ljubljana (Ljubljana): Rihard Knafelj, Marko Noc;

**SOUTH KOREA**: Hanyang University Guri Hospital (Guri-si): Tai SunPark; Seoul National University Bundang Hospital (Seongnam): Young-Jae Cho; Ewha Womans University Mokdong Hospital ICU (Seoul): Su Hwan Lee, Young Ju Lee; Ewha Womans University Mokdong Hospital SICU (Seoul): Kyung Sook Hong; Seoul National University Hospital (Seoul): Jinwoo Lee; Samsung Medical Center (Seoul): Kyeongman Jeon; Inje University Sanggye Paik Hospital (Seoul): Youjin Chang; Seoul St.Mary's Hospital (Seoul): Lee Jongmin, Kim Seok Chan; Soon Chun Hyang University Hospital Seoul (Seoul): Bo Young Lee; Severance Hospital, Yonsei University College of Medicine (Seoul): Joo Han Song; ASAN Medical Center (Seoul): Jin Won Huh; Uiheongbu St. Mary's Hospital (Uijeongbu-si, Gyeonggi-do): Lee Hwa Young; Wonju Severance Christian Hospital (Wonju): Seok Jeong Lee, Won-Yeon Lee;

**SPAIN**: Hospital Universitario Príncipe de Asturias (Alcalá de Henares): Beatriz Llorente, Maria-Consuelo Pintado; Hospital Punta de Europa (Algerciras): Irene Fernandez, Alejandro Ubeda; Hospital Universitario Fundación Alcorcón (Alcorcón ): María del Carmen Campos Moreno, Cristina Martin Dal Gesso; Hospital Universitari Germans Trias i Pujol (Badalona/Barcelona): Aroa Gomez, Pilar Ricart; CST Terrassa (Barcelona): Joaquin Amador, Maria Teresa Jurado; Hospital del Mar ICU (Barcelona): Purificación Perez-Teran, Antonia Vazquez-Sanchez; Hospital del Mar SICU (Barcelona): Adela Benitez-Cano, Jesus Carazo; Hospital de la Santa Creu i Sant Pau (Barcelona): Francisco J. Parrilla; Hospital Universitari Vall d'Hebron (Barcelona): César laborda, Oriol Roca; Hospital Universitario Santa Lucia (Cartagena): Jose Manuel Allegue, Agueda Ojados; Hospital General Universitario de Ciudad Real (Ciudad Real): M. Carmen Hornos, Mariana Portilla; Hospital Universitario del Henares (Coslada): Federico Gordo, Marcela Homez; Hospital General Universitari de Castelló (Castelló): Alberto Belenguer Muncharaz; Hospital Juan Ramón Jiménez (Huelva): Manuel Castillo Quintero, Maria Morales; Hospital Universitario de Getafe (Getafe): Demetrio Carriedo, Covadonga Rodriguez; Complejo asistencial Universitario de Leon (Leon): Silvia Avila Fuentes, Natalia Resano Sarmiento; H.G.U Gregorio Marañón (Madrid): Pablo Garcia Olivares, Alexis Jaspe Codecido; Hospital Universitario Ramon y Cajal (Madrid): Raúl de Pablo, Luis Alberto Jaramillo; Hospital Universitario 12 de Octubre UCI (Madrid): Ignacio Saez, Susana Temprano; Hospital Universitario 12 de Octubre UCI Trauma (Madrid): Isidro Prieto; Hospital Universitario La Paz (Madrid): Emilio Maseda; Fundacion Jimenez Diaz (Madrid): Cesar Perez Calvo, Anxela Vidal; Hospital de la Princesa (Madrid): Enrique Cereijo, Enrique Platas; Hospital Regional Universitario de Malaga (Malaga): Juan Luis Galeas-Lopez, Manuel Herrera-Gutierrez; Hospital Rey Juan Carlos (Mostoles): Manuel Perez; Hospital Universitario Reina Sofia de Murcia (Murcia): Eugenio Luis Palazon Sanchez; Hospital Universitario Son Espases (Palma de Mallorca): Maria Teresa Millan; Mireia Ferreruela; Hospital Universitari Son Llàtzer (Palma, Illes Ballears): Catalina Forteza, Gemma Rialp; Complejo Hospitalario de Navarra (Pamplona): Javier Izura, Juna Tirapu; Hospital Universitario Infanta Cristina (Parla): Conchita Martínez-Fidalgo; Hospital Universitari Sant Joan (Reus): Elisabet Garcia, Imma Vallverdu; Corporació Sanitària i Universitària Parc Taulí (Sabadell): Candelaria de Haro; Hospital Clínico de Salamanca (Salamanca): Felix Martin, Meisy Perez Cheng; Hospital Universitario General de Catalunya (Sant Cugat del Valles): Aitor Olmos, Roser Tomas; Hospital Sant Joan Despí Moisès Broggi (Sant Joan Despí): Diego De Mendoza, Arantxa Mas; Hospital Universitario Ntra Sra de Candelaria (Santa Cruz de Tenerife): Raquel Montiel, Dácil Parrilla; Hospital Universitario Marqués de Valdecilla (Santander): Alejandro Gonzalez-Castro; Hospital Santa Bárbara de Soria (Soria): Maria Mora Aznar, Daniel Moreno Torres; Hospital Joan XXIII (Tarragona): Neus Guasch, Mònica Magret Iglesias; National Hospital for Paraplegics (Toledo): Jesus Emilio Barrueco-Francioni; Hospital Universitario de Torrejón (Torrejon de Ardoz): Angela Algaba, Carlos Munoz de Cabo; Hospital Verge de la Cinta (Tortosa): Ferran Roche-Campo; ospital Clínico Universitario of Valencia (Valencia): Gerardo Aguilar, Carlos Ferrando; Hospital Universitario Río Hortega, Servicio de Medicina Intensiva, Unidad de Trauma y Quemados  (Valladolid): Maria Lorena Fernandez-Rodriguez, Estefania Prol-Silva; Hospital Universitario Río Hortega, Servicio de Medicina Intensiva, Unidad Polivalente  (Valladolid): David Perez-Torres, Jesus Sanchez-Ballesteros; Hospital Universitario de Alava (Vitoria-Gasteiz): Borja Fernandez, Ana Villagra; Miguel Servet Universitary Hospital (Zaragoza): Antonio Luis Ruiz-Aguilar; Hospital Clínico Universitario Lozano-Blesa (Zaragoza): Marta Asín-Corrochano, Begoña Zalba-Etayo;

**SURINAME**: s' Lands Hospitaal Paramaribo (Paramaribo): Preveen Banwarie, Bernardo Panka; Diakonessenhuis (Paramaribo): Narain Boedjawan, Yvette Chou-Lie; Academisch Ziekenhuis Paramaribo (Paramaribo): Dieneke Kienhorst, Navin Ramdhani; Sint Vincentius Hospital (Paramaribo): Dick Nahar, Alisha Van Axel;

**SWEDEN**: Falu Lasarett (Falun): Björn Ahlström, Anna Mattsson; Kristianstad Hospital (Kristianstad): Martin Spångfors; Sunderby Hospital (Luleå): Johanna Henriksson, Dan Lind; Nykopings Lasarett (Nykoping): Harald Zetterquist; Östersunds Hospital (Östersund): Helena Mansson, Line Samuelsson; Akademiska sjukhuset (Uppsala): Gaetano Perchiazzi, Magnus von Seth; Västmanlands Sjukhus Västerås (Västerås): Elena Nikolic; Västervikssjukhus (Västervik): Johan Berkius;

**SWITZERLAND**: CHUV-University Hospital of Lausanne (Lausanne): Philippe Eckert, Lise Piquilloud;

**THAILAND**: King Chulalongkorn Memorial Hospital (Bangkok): Napplika Kongpolprom; Faculty of Medicine Siriraj Hospital (Bangkok): Nuttapol Rittayamai, Krittika Teerapuncharoen; Ramathibodi Hospital 9IC Unit (Bangkok): Yuda Sutherasan, Pongdhep Theerawit; Ramathibodi Hospital 8IK Unit (Bangkok): Tananchai Petnak, Viratch Tangsujaritvijit; Ramathibodi Hospital 7NW Unit (Bangkok): Detajin Junhasavasdikul; Ramathibodi Hospital 3IC Unit (Bangkok): Cherdkiat Karnjanarachata, Sunthiti Morakul; Faculty of Medicine Vajira Hospital EMICU (Bangkok): Poungrat Thungtitigul, Konlawij Trongtrakul; Faculty of Medicine Vajira Hospital MICU (Bangkok): Yutthana Apichatbutr, Nadwipa Yuangtrakul; Thammasat University Hospital (Pathumthani): Pattarin Pirompanich, Narongkorn Saiphoklang;

**TUNISIA**: Hôpital A. Mami (Ariana): Mohamed Besbes, Amira Jammoussi; Mongi Slim Hospital (La Marsa): Asma Ben Souissi, Mhamed Sami Mebazaa; Taher Sfar Hospital Mahdia (Mahdia): Souheil Elatrous, Nejla Tilouch;

**TURKEY**: Çukurova University Balcalı Hospital Interal Medicine ICU (Adana): Didem Sozutek; Çukurova University Hospital Respiratory ICU (Adana): Ozlem Ozkan Kuscu, Ezgi Özyılmaz; Adana City Training and Research Hospital (Adana): Avşar Zerman; Niğde Training and Research Hospital (Ankara): Sema Sari; Ministry of Health Ankara City Hospital (Ankara): Sema Turan; University of Health Sciences, Atatürk Chest Diseases and Thoracic Surgery Training and Research Hospital (Ankara): Semih Aydemir, Hilal Sazak; Gazi University Hospital (Ankara): Gulbin Aygencel, Melda Turkoglu; 'University of Health Sciences, Dışkapı Yıldırım Beyazıt Research and Training Hospital, Department of Surgical Intensive Care Unit (Ankara): Fatma Yildirim; Akdeniz University Hospital (Antalya): Melike Cengiz, Ayca Gumus; Medicine School of Ege University Hospital (Bornova/Izmir): Feza Bacakoglu, Pervin Korkmaz Ekren; Uludag University (Bursa): Nermin Kelebek Girgin, Ayşe Nur Soyturk; Düzce University Hospital (Düzce): Türkay Akbas; Trakya Universty Hospital (Edirne): Serdar Efe, Volkan Inal; Dr Ersin Arslan Eğitim ve Araştırma Hastanesi (Gaziantep): Gülseren Elay; Marmara University Hospital Medical Intensive Care Unit (Istanbul): Huseyin Arikan, Sait Karakurt; Marmara University Hospital Anesthesiology and Reanimation (Istanbul): Ismail Cinel, Fethi Gül; Cerrahpasa Medical School Hospital (Istanbul): Suha Bozbay, Oktay Demirkiran; Istanbul University Cerrahpasa Medical Faculty (Istanbul): Yalim Dikmen, Elif Erdogan; Istanbul University Medical Faculty (Istanbul): Perihan Ergin Ozcan, Figen Esen; Sureyyapasa Chest Diseases and Thoracic Surgery Training Hospital (Istanbul): Nalan Adiguzel, Ozlem Yazicioglu Mocin; Sisli Etfal Educational Research Hospital (Istanbul): Mustafa Akker; Bakırköy Dr. Sadi Konuk Traning and Researching Hospital (Istanbul): Zafer Çukurova, Yasemin Seker Tekdos; Dr. Suat Seren Chest Diseases and Surgery Training Hospital (Izmir): Cenk Kirakli; Konya Numune Hastanesi (Konya): Iskender Kara; Erciyes University Hospital Faculty of Medicine Dep. Critical Care (Kayseri): Faruk Seçkin Yücesoy; Van Yüzüncü Yil University, School of Medicine, Department of Anesthesiology and Reanimation (Van): Hilmi Demirkiran, Arzu Esen Tekeli;

**UNITED KINGDOM:** Stoke Mandeville Hospital (Aylesbury): Pradeep Shanmugasundaram; Barnsley Hospital NHS Foundation Trust (Barnsley): Simon Chau, Sughrat Siddiqui; Royal United Hospitals Bath NHS Foundation Trust (Bath): Tim Cook, Ian Kerslake; Bedford Hospital NHS trust (Bedford): Sarah Snape, Ana Vochin; Heartlands Hospital, University Hospitals Birmingham (Birmingham): Gavin Perkins, Elliot Yates; Royal Sussex County Hospital (Brighton): Owen Boyd, Laura Ortiz-Ruiz De Gordoa; Southmead Hospital (Bristol): Caroline Kane, Matt Thomas; Bristol Royal Infirmary (Bristol): Jeremy Bewley, Lisa Grimmer; Burton Hospitals NHS FT (Burton on Trent): Paul Smith; West Suffolk Hospital NHS Foundation Trust (Bury St Edmunds): Kaushik Bhowmick, Sally Humphreys; NCUH Trust (Carlisle): Tim Smith, Antoinette Wilson; Chesterfield Royal NHS Foundation Trust (Chesterfield): Sarah Beavis, Nick Spittle; Leighton Hospital (Crewe): Phil Chilton, Clare Hammel; Croydon Health Services NHS Trust (Croydon): Sundar Raj Ashok, Arif Moghal; Dumfries and Galloway Royal Infirmary (Dumfries): David Wrathall; Queen Elizabeth University Hospital (Glasgow): Christopher Wright; Royal Surrey County Hospital (Guildford): David Slessor; The Princess Alexandra Hospital NHS Trust (Harlow): Dagmar Holmquist, Rajnish Saha; Harrogate District Hospital NHS Foundation Trust (Harrogate): Lorraine Stephenson; North West Anglia NHS Foundation Trust Hinchingbrooke Hospital (Huntingdon): Tamaas Leiner; Royal Glamorgan Hospital (Llantrisant): Andrew Hermon, Ceri Lynch; St James's University Hosptial (Leeds): Simon Whiteley, Elizabeth Wilby; Royal Liverpool University Hospital (Liverpool): Ingeborg Welters, Karen Williams; King's College Hospital (London): Rohit Saha, Grisma Patel; Hillingdon Hospital (London): Elisa Kam; Queen Elizabeth Hospital (London): Amy Collins, Ahmed Zaki; Guy's and St Thomas' Hospitals (London): Reza Khorasanee; Salford Royal NHS Foundation Trust (Manchester): Elliot Bertram-Ralph, Daniel Horner; North Manchester General Hospital (Manchester): Jayaprakash Patil, Christos Chaintoutis; James Cook University Hospital (Middlesbrough): Keith Hugill, Isabel Gonzalez; Milton Keynes University Hospital (Milton Keynes): Jane Adderley, Alex Martin; Glan Clwyd Hospital (Rhyl): Richard Pugh, Venkat Sundaram; The Rotherham NHS Foundation Trust (Rotherham): Anil Hormis, Mark Smith; South Tyneside NHS Foundation Trust (South Shields): Govindan Balaraj, Riccardo Scano; Lister Hospital (Stevenage): Sunil Jamadarkhana; University Hospital North Tees (Stockton-on-Tees): Rakesh Bhandary, Michele Clark; Musgrove Park (Taunton): Patricia Doble, Richard Innes; Torbay Hospital (Torquay): Thomas Clark; Royal Cornwall Hospital (Truro): Karen Burt, Mike Spivey; Pinderfields Hospital (Wakefield): Alastair Rose; Ulster Hospital (Belfast): Samantha Hagan, John Trinder; Yeovil District Hospital (Yeovil): Agnieszka Kubisz-Pudelko;

**URUGUAY**: CASMU (Montevideo): Gaston Aguirre, Alberto Deicas; Hospital de Clinicas (Montevideo): Rodrigo Beltramelli, Arturo Briva;

**UNITED STATES**: Massachussetts General Hospital (Boston): Jarone Lee, Gabriel Rodriguez; Beth Isreal Deaconess Medical Center (Boston): Elias Baedorf Kassis, Valerie Banner-Goodspeed; John H Stroger Hospital of Cook County (Chicago) Renaud Gueret, Aiman Tulaimat; University of Cincinnati Medical Center (Cincinnati): Dina Gomaa, Betty Tsuei; Cleveland Clinic Foundation (Cleveland): Abhijit Duggal, Ashish K. Khanna; The Ohio State University Wexner Medical Hospital (Columbus): Joshua A. Englert, Michael Wert; El Centro Regional Medical Center (El Centro): Christian Tomaszewski, Gabriel Wardi; University of California San Diego – Jacobs Medical Center (La Jolla): Jeffrey Barry, Christine M. Bojanowski; UCSD Sulpizio (La Jolla): Nancy Glober, Christopher Tainter; NSMC Union Hospital (Lynn): Elizabeth Stevenson; Froedtert and the Medical College of Wisconsin (Milwaukee): Rahul Nanchal, Jonathon Truwit; Intermountain Medical Center (Murray): Colin Grissom, Michael Lanspa; Vanderbilt University Medical Center (Nashville): Andrew McKown, Todd Rice; Tulane Medical Center (New Orleans): Shigeki Saito; Oregon Health & Science University (Portland): Akram Khan, Stephanie Nonas; Mayo Clinic St Marys Surgical ICU (Rochester): Philippe Bauer, Nathan Smischney; Mayo Clinic St Marys Campus Medical ICU (Rochester): Philippe Bauer, Richard Oeckler, Mayo Clinic Methodist Campus Medico-Surgical and Transplant ICU (Rochester): Philippe Bauer, Richard Oeckler; North Shore Medical Centre (Salem): Ashish Rai; University of California San Diego Medical Center – Hillcrest (San Diego): Kevin Eng, Sanjeev Tyagi; Regions Hospital (St Paul): David Dries, Elizabeth Ramey; Stanford Hospital (Stanford): Angela Rogers, Jack Short;
