# Supplementary material for: Correction: Impact of frailty and older age on weaning from invasive ventilation: a secondary analysis of the WEAN SAFE study
Source: Ann Intensive Care. 2025 Apr 28;15:58. doi: 10.1186/s13613-025-01467-7 (PMC12037443; doi:10.1186/s13613-025-01467-7)
Supplement: Supplementary file 1 — Supplementary Material 1. [file 13613_2025_1467_MOESM1_ESM.docx]

Gaëtan Beduneau, Giacomo Bellani, Laurent Brochard, Ewan Goligher, Giacomo Grasselli, Leo Heunks, John Laffey, Fabiana Madotto, Jordi Mancebo, Antonio Pesenti, Tai Pham, Lise Piquilloud, Hannah Wunsch, Frank van Haren, Elisa Estenssoro**,** Frank Van Haren, Greet Hermans, Ary Serpa Neto, Haibo Qiu, Ewan Goligher, Guillermo Bugedo, Vladimir Cerny, Assem Abdel Razek, Gaëtan Beduneau**,** Sébastien Perbet, Onnen Moerer, Dimitrios Matamis, Alfred Papali, Zsolt Molnar, Pravin Amin, Seyed Mohammadreza Hashemian, Kevin Clarkson, Giacomo Grasselli, Kiyoyasu Kurahashi, Subhash P Acharya, Asisclo Villagomez, Amine Ali Zeggwagh, Leo M Heunks, Jon Henrik Laake, Rollin Roldan, Konstanty Szuldrzynski, Irene Aragao, Dana Tomescu, Alexey Gritsan, Yaseen Arabi, Bojan Jovanovic, Young-Jae Cho, Óscar Peñuelas, Bernardo Panka, Johan Berkius, Lise Piquilloud, Nuttapol Rittayamai, Fekri Abrough, Ezgi Ozylmaz, Luigi Camporota, Philippe Bauer, Daniel Talmor, Jeremy Beitler, Alma Cani, Sebastian Fredes, Santiago Ilutovich, Marco Bezzi, Silvina Borello, Gustavo Plotnikow, Romina Pratto, Nicolas Iezzi, Rodolfo Lopez, Mariano Andres Furche, Paolo Nahuel Rubatto Birri, Pablo Lovazzano, Mariano Setten, Matilde Grando, Vanesa Alejandra Pavlotsky, Daniela Benvenuti, Eliana Markman, Graciela Paz, Aldana Ruiz Robledo, Raúl Alejandro Gomez, María Florencia Valenti, Anatilde Diaz, Analía Garcia, Rosana Hernandez, Maria Cristina Orlandi, Juan Conde, Rosa Reina, Gustavogcha Chaparro, Gonzalo Pagella, Graciela Zakalik, Carlos Pellegrini, Ariel Chena, Maria Fernanda Farina, Claudia Elizabeth Lopez, Fernando Rios, Judith Sagardia, María Elena Romano, Cristina Villegas Succar, Lisandro Roberto Bettini, Luis Pablo Cardonnet, Fernando Rios, Alejandro Risso Vazquez, Ramón Carrillo, Robert Giannoni, Gabriela Bai, Santiago Izza, Miguel Escobar, Patricia Sanchez, Andrew Bersten, Shailesh Bihari, Dianne Hill, Angus Richardson, Graeme Duke, Stephanie Hunter, Bernie Bissett, Frank Van Haren, Mark Kol Kol, Asim Shah, Peter Oziemski, Deborah Welsh, Vijayanand Palaniswamy, Kathryn Kerr, Ameet Parekh, Ege Eroglu, Adrian Regli, Edward Fysh, David Blythe, Muraleekrishnan Muthukrishnan, Janet Ferrier, Edward Litton, Gabrielle Hanlon, Jonathan Barrett, James McCullough, Mandy Tallott, Shihan Mahmud Redwanul Huq, Raihan Rabbani, Eric Frans, Helga Ceunen, Greet Hermans, Filiep Soetens, Marc Vanhoof, Pierre Bulpa, Isabelle Michaux, Mónica Crespo Ramirez, Orlando Gordillo Romero, Sanja Granov Grabovica, Slavenka Straus, Bruno Vilela Costa Pinto, Maria Augusta Rahe Pereira, Tamasato Tamasato, Wilson Oliveira Filho, Jocyelle Vieira, Fernanda Kutchak, Marcelo Rieder, Fabianne Dantas, Louise Gondim, Luciano Azevedo, Leandro Taniguchi, Fernando da Silva Ramos, Ary Serpa Neto, Karina Timenetsky, Stephanie Piras, Claudio Piras, Eliana Caser, Betania Silva Sales, Margarita Borislavova, Karen J Bosma, Michael Mikhaeil, Andrew Seely, Laurent Brochard, Tai Pham, Ricard Mellado Artigas, Thomas Piraino, Phil Shin, Sharique Ansari, Victoria McCredie, Daniel Arellano, Rodrigo Cornejo, Matias Jesús Flamm Zamorano, Manuel Gonzalez, Guillermo Bugedo, David Carpio, Jerónimo Graf Santos, Rodrigo Pérez-Araos, Eduardo Labarca, Felipe Martinez, Bin Zhu**,** Guojun Pan, Chen Shuhua, Jiuzhi Zhang, Kai Chen, Rongguo Yu, Tiehe Qin, Shouhong Wang, Xiang-Dong Guan, Jian-Feng Wu, Bi-Lin Wei, Feng Feng, Meihong Hou, Hongwen Zhang, Chuanyun Qian, Wei Zhang, Jia Zheng, Zheng-Jiang Xing, Dahuan Li, Guoxiu Zhang, Qing Gu, Ning Liu, Ling Liu, Haibo Qiu, Chengqing Mei, Zhenglong Ye, Liangyan Jiang, Zhanhong Tang, Chenliang Sun, Hongsheng Zhao, Wu Dawei, Guo Xi, Jialin Liu, Hongping Qu, Wang Ruilan, Xie Yun, Bin Zang, Hua Luo, Weixin Zhang, Wensen Pan, Boyun Yuan, Yufeng Feng, Min Lu, Xia Hongtao, Gong Yu, You Shang, Xiaobo Yang, Yupeng Qi, Tao Yu, Hongyang Xu, Jie Yan, Chen Jing, Zhang Minwei, Hongbin Li, Rongqing Sun, Mónica Vargas-Ordoñez, Juan Ignacio Silesky Jimenez, Hernan Aguirre-Bermeo, Diego Rolando Morocho Tutillo, Andrea Gabriela Peña Padilla, Diana Alvarez, María Fernanda Garcia, Mohamed Elsaadany, Hany Elsayed, Samar Elsayed, Abdelrhman Aboshady, Nagwa Doha, Eman Shebl, Philippe Crova, Thuy Nga Phan, Simon Bocher, Gwenael Prat, Marc Danguy des Déserts, Françoise Labat, Cédric Daubin, Aurélie Joret, Bertrand Sauneuf, Xavier Souloy, Malo Emery, Damien Roux, Frédérique Schortgen, Pierre-Louis Declercq, Stéphanie Gelinotte, Louis-Marie Galerneau, Nicolas Terzi, Frank Chemouni, Jonathan Zarka, Nicolas Chudeau, Saad Nseir, Anahita Rouze, Matthieu Le Meur, Martial Thyrault, Claude Guérin, Jonathan Chelly, Sébastien Jochmans, Pierre-Eric Danin, Jean Dellamonica, Alexandre Robert, Virginie Lemiale, Cédric Bruel, François Philippart, Jean-François Llitjos, Nathalie Marin, Muriel Fartoukh, Guillaume Voiriot, Emmanuel Guerot, Maxens Decavèle, Martin Dres, Faustine Reynaud, Arnaud W Thille, Alexandre Tonnelier, Pascal Beuret, Sébastien Ena, Philippe Gouin, Pierre-Gildas Guitard, Gaëtan Beduneau, Elisabeth Surlemont, Gabriel Preda, Daniel Silva, Laurence Dangers, Jean-Etienne Herbrecht, Francis Schneider, Jean-Michel Arnal, Aude Garnero, Julio Badie, Loïc Barrot, Onnen Moerer, Philipp M Lepper, Frederik Seiler, Metaxia Papanikolaou, Theonymfi Papavasilopoulou, Olympia Apostolopoulou, Chrysi Diakaki, Panagiotis Ioannides, Marina Oikonomou, Eleni Massa, Eleni Mouloudi, Aikaterini Dimoula, Sofia Nikolakopoulou, Stacy House, Monaly Rivette , Csaba Kopitko, László Medve, Zoltan Kulcsar, Zsuzsanna Szabo, Zsolt Molnar, Nándo Öveges, Agnes Sarkany, Shuchi Kaushik, Bhagyesh Shah, Radhakrishnan Muthuchellappan, Ramesh Vj, Saroj Pattnaik, Banambar Ray, Sanghamitra Mishra, Basanta Kumar Pati, Sivakumar Nandakumar, Lakshmikanthcharan Saravanabavan, Lakshay Bhakhtiani, Simant Jha, Vijay Kumar Agrawal, Prakash Khairnar, Srinivas Samavedam, Arvind Baronia, Mohan Gurjar, Mayur Patel, Darshana Rathod, Harshal Bawangade, Deepak Jeswani, Harish Mallapura Maheswarappa, Seyed Mohammadreza Hashemian, Hamidreza Jamaati, Laura Flood, Alistair Nichol, Ignacio Martin-Loeches, Lindi Snyman, Kevin Clarkson, Rooney Grainne, Catherine Motherway, Don Walsh, Mohammad Faheem, Salvatore Grasso, Rossella di Mussi, Alessandra Nasi, Ivano Riva, Elisabetta Pierucci, Rocco D’Andrea, Elisabetta Pecci, Rinaldo Grasso, Gianmario Monza, Jessica Maugeri, Agrippino Bellissima, Eugenio Garofalo, Paolo Navalesi, Massimo Zambon, Paolo Gnesin, Manuel Todeschini, Salvatore Maurizio Maggiore, Luca Serano, Stefano Muttini, Eduardo Beck, Alberto Facchini, Luca Guatteri, Savino Spadaro, Carlo Alberto Volta, Cosimo Chelazzi, Gilda Cinnella, Lucia Mirabella, Alexandre Molin, Fabio Tarantino, Andrea Coppadoro, Ettore Vascotto, Francesca Orsenigo, Virginia Porta, Davide Chiumello, Giovanni Mistraletti, Antonio Castelli, Riccardo Colombo, Francesco Curto, Roberto Fumagalli, Riccardo Pinciroli, Giacomo Grasselli, Monica Savioli, Maurizio Bottiroli, Maurizio Pavesi, Giacomo Bellani, Carlo Oliveri, Rosanna Vaschetto, Pietro Caironi, Giacomo Berta, Bruno Ballico, Giovanni Vitale, Paolo Persona, Tommaso Tonetti, Sabrina Boraso, Laura Pasin, Andrea Cortegiani, Mariachiara Ippolito, Andrea Neville Cracchiolo, Maria Teresa Strano, Edoardo Picetti, Emanuele Sani, Mirko Belliato, Giorgio Antonio Iotti, Anna Aliberti, Francesco Mojoli, Angelo Giacomucci, Antonella Frattari, Pietro Bertini, Fabio Guarracino, Iacopo Cappellini, Guglielmo Consales, Maurizio Fusari, Gianluca Zani, Andrea Bruni, Sebastiano Macheda, Laura Bernabe, Edoardo Piervincenzi, Marco Ranieri, Gennaro De Pascale, Luca Montini, Roberta Caccese, Yari Gollo, Valeria Lascari, Antonella Fortunato, Salvatore Palmese, Marco Spagnoli, Simone Maria Zerbi, Leda Floris, Pierpaolo Terragni, Stefano Clementi, Rosella Barbieri, Lucia Cubattoli, Vito Fanelli, Gabriele Sales, Stefania Sovatzis, Massimo Borelli, Federica Vagginelli, Paolo Chiarandini, Manuela Lugano, Stefania Buttera, Andrea Gigante, Francesca Lucchese, Domenico Gelormini, Elisa Boni, Silvia De Rosa, Moe Oguchi, Tomohito Sadahiro, Yukako Obata, Sakuraya Masaaki, Akihiro Takaba, Shinichiro Ohshimo, Nobuaki Shime, Hidenobu Kamohara, Hiromasa Irie, Koichi Arinaga, Shuhei Niiyama, Katsunori Mochizuki, Kenichi Nitta, Tetsuya Yumoto, Akira Kuriyama, Misuzu Nakanishi, Masamitsu Sanui, Junji Kumasawa, Takuya Shiga, Norifumi Yoshida, Shinshu Katayama, Taiga Itagaki, Kiyoyasu Kurahashi, Kazuya Omura, Kengo Asano, Kei Ota, Kotaro Yamamoto, Daisuke Taniguchi, Jun Kataoka, Hiroki Iriyama, Toshikazu Abe, Izumi Nakayama, Isao Nagata, Mohamed Benlamin, Abubaker S Elmaryul, Felipe de Jesus Montelongo, Victor Hugo Madrigal Robles, Daniel Rodriguez Gonzalez, Silvio Antonio Namendys-Silva, Claudia Lopez Nava, Nandyelly San Juan Roman, Maria del Carmen Marin, Asisclo Villagomez, Nancy Canedo, Alejandro Esquivel, Carmen Hernandez, Gustavo Lugo Goytia, Antonio Landaverde Lopez, Miguel Ángel Sosa Medellin, Anaid Manzano, Abdellatif Benslama, Hanane Ezzouine, Abdelhamid Hachimi, Brahim Housni, Tarek Dendane, Abidi Khalid, Doumiri Mouhssine, Maazouzi Wajdi, Subhash Acharya, Anand Thakur, Prabha Gautam, Leo Heunks, Ingrid van den Hul, Luigi Pisani, Marcus J Schultz, Martin Rinket, Jan Wytze Vermeijden, Melanie Acampo-de Jong, Serge Heines, Tim Frenzel, Hans van der Hoeven, Nardo Van Der Meer, Dolf Weller, Koen Simons, Rachael Parke, Shay McGuinness, Carmel Chapman, Andrew Stapleton, Ulrike Buehner, Erin Williams, Nina Beehre, Finn H Andersen, Brit Ågot Sjøbø, Gabriele Leonie Schwarz, Knut Dybwik, Bror Anders Johnstad, Terje Legernaes, Ole Georg Vinorum, Nils Christian Ween-Velken, Martin Fluckiger, Lutz Fehrle, Tayyba Naz Aslam, Jon Henrik Laake, Linda Rørtveit, Kristian Strand, Muneeb Ali, Taha Pasha, Rakhshanda Jabeen, Kamal Nasir, Cecilia Eugenia Chavez, Patricia Gutierrez, Tapia Muñoz, Jorge Cabrera, Willy Porras, Luis Coaguila, Giovanna Soto, Rosita Gomero Paredes, Martin Santos, Jesus Milagrito Avalos Cabrera, Ivan Canchos Gutierrez, Hector Higo Leon Yoshido, Ronald Zumaran, Guillermo Malpartida, José Portugal, Gabriel Omar Heredia Orbegoso, Xandra Yanina Rodriguez Tucto, Ronald Perez Maita, Rocio Quispe Soto, Helbert Esquivel Gallegos, José Cruz, Enrique Paz, Willy Diaz, Oscar Gomez, Rainier Ovalle Olmos, Rosari Quispe Sierra, Peter Malaga, Yazcitk Sandoval, Manuel Alberto Laca Barrera, Fernando Pachas Alvarado, Teobaldo Quintana, Julio Yáñez, Luis Herrera, Olga Milagros, Mestanza Arica, Piotr Czempik, Milosz Jankowski, Konstanty Szuldrzynski, Jaroslaw Garlicki, Wojciech Serednicki, Jadwiga Wojtas, Jakub Smiechowicz, Nuno Catorze, Tiago Pereira, Rui Gomes, Vera Pereira, Cristina Coxo, Luis Bento, Sara Ventura, Vitor Mendes, Pedro Povoa, Maksym Dykyy, Juan Hidalgo, Maria Teresa Oliveira, Ana Vaz, Heloisa Castro, Maria João Ferreira da Silva, Tiago Leonor, Elsa Sousa, João Carvalho, Guilherme Domingos, Ana Raquel Lima, Igor Milet, Luis Patão, Carla Santos, Andrey Malyarchikov, Konstantin Shapovalov, Andrey Gazenkampf, Alexey Gritsan, Marina Petrova, Maria Vatsik, Pavel Dunts, Oleg Li, Maie Salem, Ghamdan Al Sadeh, Mohamed Mustafa, Yaseen Arabi, Sultan Alamri, Mohamed Rabee, Ahmed Rabie, Mostafa Rajab, Mohamed Khalaf Ebraheim Mervat, Ismael Marey, Adi Hadzibegovic, Bojan Jovanovic, Branislava Stefanovic, Rihard Knafelj, Marko Noc, Tai SunPark, Young-Jae Cho, Su Hwan Lee, Young Ju Lee, Kyung Sook Hong, Jinwoo Lee, Kyeongman Jeon, Youjin Chang, Lee Jongmin, Kim Seok Chan, Bo Young Lee, Joo Han Song, Jin Won Huh, Lee Hwa Young, Seok Jeong Lee, Won-Yeon Lee, Beatriz Llorente, Maria-Consuelo Pintado, Irene Fernandez, Alejandro Ubeda, María del Carmen Campos Moreno, Cristina Martin Dal Gesso, Aroa Gomez, Pilar Ricart, Joaquin Amador, Maria Teresa Jurado, Purificación Perez-Teran, Antonia Vazquez-Sanchez, Adela Benitez-Cano, Jesus Carazo, Francisco J. Parrilla, César Laborda, Oriol Roca, Jose Manuel Allegue, Agueda Ojados, M Carmen Hornos, Mariana Portilla, Federico Gordo, Marcela Homez, Alberto Belenguer Muncharaz, Manuel Castillo Quintero, Maria Morales, Demetrio Carriedo, Covadonga Rodriguez, Silvia Avila Fuentes, Natalia Resano Sarmiento, Pablo Garcia Olivares, Alexis Jaspe Codecido, Raúl de Pablo, Luis Alberto Jaramillo, Ignacio Saez, Susana Temprano, Isidro Prieto, Emilio Maseda, Patricia Salgado, Cesar Perez Calvo, Anxela Vidal, Enrique Cereijo, Enrique Platas, Juan Luis Galeas-Lopez, Manuel Herrera-Gutierrez, Manuel Perez, Eugenio Luis Palazon Sanchez, Maria Teresa Millan, Mireia Ferreruela, Catalina Forteza, Gemma Rialp, Javier Izura , Juna Tirapu, Conchita Martínez-Fidalgo, Elisabet Garcia, Imma Vallverdu, Candelaria de Haro, Felix Martin, Meisy Perez Cheng, Aitor Olmos, Roser Tomas, Diego De Mendoza, Arantxa Mas, Raquel Montiel, Dácil Parrilla, Alejandro Gonzalez-Castro, Maria Mora Aznar, Daniel Moreno Torres, Neus Guasch, Mònica Magret Iglesias, Jesus Emilio Barrueco-Francioni, Angela Algaba, Carlos Munoz de Cabo, Ferran Roche-Campo, Gerardo Aguilar, Carlos Ferrando, Maria Lorena Fernandez-Rodriguez, Estefania Prol-Silva, David Perez-Torres, Jesus Sanchez-Ballesteros, Borja Fernandez, Ana Villagra, Antonio Luis Ruiz-Aguilar, Marta Asín-Corrochano, Begoña Zalba-Etayo, Preveen Banwarie, Bernardo Panka, Narain Boedjawan, Yvette Chou-Lie, Dieneke Kienhorst, Navin Ramdhani, Dick Nahar, Alisha Van Axel, Björn Ahlström, Anna Mattsson, Martin Spångfors, Johanna Henriksson, Dan Lind, Harald Zetterquist, Helena Mansson, Line Samuelsson, Gaetano Perchiazzi, Magnus von Seth, Elena Nikolic, Johan Berkius, Philippe Eckert, Lise Piquilloud, Napplika Kongpolprom, Nuttapol Rittayamai, Krittika Teerapuncharoen, Yuda Sutherasan, Pongdhep Theerawit, Tananchai Petnak, Viratch Tangsujaritvijit, Detajin Junhasavasdikul, Cherdkiat Karnjanarachata, Sunthiti Morakul, Poungrat Thungtitigul, Konlawij Trongtrakul, Yutthana Apichatbutr, Nadwipa Yuangtrakul, Pattarin Pirompanich, Narongkorn Saiphoklang, Mohamed Besbes, Amira Jamoussi, Asma Ben Souissi, Mhamed Sami Mebazaa, Souheil Elatrous, Nejla Tilouch, Didem Sozutek, Ozlem Ozkan Kuscu, Ezgi Özyılmaz, Avşar Zerman, Sema Sari, Sema Turan, Semih Aydemir, Hilal Sazak, Gulbin Aygencel, Melda Turkoglu, Fatma Yildirim, Melike Cengiz, Ayca Gumus, Feza Bacakoglu, Pervin Korkmaz Ekren, Nermin Kelebek Girgin, Ayşe Nur Soyturk, Türkay Akbas, Serdar Efe, Volkan Inal, Gülseren Elay, Huseyin Arikan, Sait Karakurt, Ismail Cinel, Fethi Gül, Suha Bozbay, Oktay Demirkiran, Yalim Dikmen, Elif Erdogan, Perihan Ergin Ozcan, Figen Esen, Nalan Adiguzel, Ozlem Yazicioglu Mocin, Mustafa Akker, Zafer Çukurova, Yasemin Seker Tekdos, Cenk Kirakli, Iskender Kara, Faruk Seçkin Yücesoy, Hilmi Demirkiran, Arzu Esen Tekeli, Pradeep Shanmugasundaram, Simon Chau, Sughrat Siddiqui, Tim Cook, Ian Kerslake, Sarah Snape, Ana Vochin, Gavin Perkins, Elliot Yates, Owen Boyd, Laura Ortiz-Ruiz De Gordoa, Caroline Kane, Matt Thomas, Jeremy Bewley, Lisa Grimmer, Paul Smith, Kaushik Bhowmick, Sally Humphreys, Tim Smith, Antoinette Wilson, Sarah Beavis, Nick Spittle, Phil Chilton, Clare Hammel, Sundar Raj Ashok, Arif Moghal, David Wrathall, Christopher Wright, David Slessor, Dagmar Holmquist, Rajnish Saha, Lorraine Stephenson, Tamaas Leiner , Andrew Hermon, Ceri Lynch, Simon Whiteley, Elizabeth Wilby, Ingeborg Welters, Karen Williams, Rohit Saha, Grisma Patel, Elisa Kam, Amy Collins, Ahmad Zaki, Reza Khorasanee, Elliot Bertram-Ralph, Daniel Horner, Jayaprakash Patil, Christos Chaintoutis, Keith Hugill, Isabel Gonzalez, Jane Adderley, Alex Martin, Richard Pugh, Venkat Sundaram, Anil Hormis, Mark Smith, Govindan Balaraj, Riccardo Scano, Sunil Jamadarkhana, Rakesh Bhandary, Michele Clark, Patricia Doble, Richard Innes, Thomas Clark, Daniel Paul, Karen Burt, Mike Spivey, Alastair Rose, Samantha Hagan, John Trinder, Agnieszka Kubisz-Pudelko, Jarone Lee, Gabriel Rodriguez, Elias Baedorf Kassis, Valerie Banner-Goodspeed, Renaud Gueret, Aiman Tulaimat, Dina Gomaa, Betty Tsuei, Abhijit Duggal, Ashish K. Khanna, Joshua A. Englert, Michael Wert, Christian Tomaszewski, Gabriel Wardi, Jeffrey Barry, Christine M. Bojanowski, Nancy Glober, Christopher Tainter, Elizabeth Stevenson, Rahul Nanchal, Jonathon Truwit, Colin Grissom, Michael Lanspa, Andrew McKown, Todd Rice, Shigeki Saito, Akram Khan, Stephanie Nonas, Philippe Bauer, Nathan Smischney, Richard Oeckler, Ashish Rai, Kevin Eng, Sanjeev Tyagi, David Dries, Elizabeth Ramey, Angela Rogers, Jack Short, Gaston Aguirre, Alberto Deicas, Rodrigo Beltramelli, Arturo Briva,
